# Equine Welfare in Practice: A Collaborative Outreach and Education Program with Michigan State University, Universidad Nacional Autónoma de México, and Universidad Veracruzana

**DOI:** 10.3390/ani9040164

**Published:** 2019-04-13

**Authors:** Harold C. Schott, Alejandro Estrada-Coates, Miriam Alva-Trujillo, Annette D. Petersen, Marc A. Kinsley, Melissa M. Esser, Jose Casillas, Elena Garcia-Seco, Mauro Madariaga-Najera, José Antonio Fernando Martínez, Arturo Herrera-León, Mariano Hernández-Gil

**Affiliations:** 1 Department of Large Animal Clinical Sciences, College of Veterinary Medicine, Michigan State University, 736 Wilson Road, East Lansing, MI 48824, USA; peter240@msu.edu (A.D.P.); kinsley1@msu.edu (M.A.K.); essermel@msu.edu (M.M.E.); casill15@msu.edu (J.C.); 2 Facultad de Medicina Veterinaria y Zootecnia, Universidad Veracruzana, Miguel Ángel de Quevedo s/n, esquina Yáñez, Colonia Unidad Veracruzana, Veracruz, Veracruz C.P. 91710, Mexico; aestrada@uv.mx (A.E.-C.); mialva@uv.mx (M.A.-T.); 3 Facultad de Medicina Veterinaria y Zootecnia, Universidad Nacional Autónoma de México, Ciudad Universitaria, D.F. México C.P. 04501, Mexico; elenitags.eg@gmail.com (E.G.-S.); marianohg.mexico@gmail.com (M.H.-G.); 4 Facultad de Medicina Veterinaria y Zootecnia, Segundo edificio, Planta Baja, Universidad Nacional Autónoma de México, Ciudad Universitaria, D.F. México C.P. 04501, Mexico; madariaganm@hotmail.com (M.M.-N.); fm1577@yahoo.com.mx (J.A.F.M.); arturher9@hotmail.com (A.H.-L.)

**Keywords:** donkey, mule, horse, equid, working, preventive medicine, farrier, dentistry, castration, wound

## Abstract

**Simple Summary:**

There is great need for veterinary care for working equids worldwide. Further, students in the United States (US) need more primary care experience with equids in their veterinary curricula. To address these needs we developed a collaborative “Equine Welfare in Practice” outreach and education project to provide veterinary care to working equids in Mexico, while at the same time providing an opportunity for veterinary students to gain “hands-on” experience with equids. Veterinarians from Michigan State University, Universidad Nacional Autónoma de México, and Universidad Veracruzana developed a two-week community based project in which US and Mexican veterinary students work as teams, supervised by veterinarians, to provide care to working equids in rural Mexican communities. From 2017 through 2019, 24 US students and 25 Mexican students, interns and residents examined, vaccinated and dewormed more than 2200 equids and performed more than 80 castrations, 100 rectal palpations for pregnancy diagnosis, 220 dental floats and 320 hoof trims. The project is largely supported by private donors with supplies provided by several pharmaceutical companies. Overall, the project has exceeded all expectations and future directions include implementation of community based engaged research and exchange programs for post-graduate veterinary training.

**Abstract:**

There is great need for veterinary care for working equids worldwide. Addressing this need provides an opportunity for veterinary students to gain primary care experience. An annual two week collaborative outreach and educational program with Michigan State University (MSU), the Universidad Nacional Autónoma de México (UNAM) and the Universidad Veracruzana (UV) was developed to provide care for working equids in rural Mexican communities. From 2017 to 2019 24 US veterinary students and 25 Mexican veterinary students, interns and residents examined, vaccinated and dewormed more than 2200 equids and performed more than 80 castrations, 100 rectal palpations for pregnancy diagnosis, 220 dental floats and 320 hoof trims. They also treated many wounds, sarcoids, vampire bat bites and tick infestations and also saw unusual cases including tetanus, eye injuries, nuchal bursitis, cervical vertebral malformation and suspected vesicular stomatitis. Development of the collaborative MSU-UNAM-UV Equine Welfare in Practice Clerkship required vision, learning, relationship building, creativity, fund-raising and perseverance to develop and agree on mutually beneficial objectives for all participants. The project is largely financed through private donations and supplies provided by pharmaceutical companies. The outcome has been a highly successful program that could be used as a model by other Colleges of Veterinary Medicine world-wide.

## 1. Introduction

Nearly 90% of the world’s equid population resides in third world countries where donkeys, mules and horses are used for work to improve the socioeconomic status of impoverished families. It is estimated that there are more than 100 million working equids supporting more than 600 million people worldwide [1,2,3]. Despite growth and expanded efforts of equine welfare organizations across the globe, the vast majority of these working equids still receive no care by veterinarians, qualified farriers, animal scientists or nutritionists. Unfortunately, many owners of working equids lack the knowledge of how to fully care for their equids, leading to animal welfare issues [4,5,6]. An even greater concern is the lack of recognition, or perhaps acknowledgement, by the developed world of the magnitude of the problem. 

Over the past couple of decades welfare-minded equine practitioners in North America have developed independent projects to provide veterinary care for working equids in the Caribbean Islands, Mexico, Latin America and South America. These individuals volunteer their time and equipment and sometime receive support in the form of product (e.g., vaccines and anthelementic medications) from pharmaceutical corporations. About a decade ago, several of these veterinarians joined forces to compare their experiences during the annual convention of The American Association of Equine Practitioners (AAEP). This group subsequently launched the AAEP Equitarian Initiative, with the term Equitarian evolving as a merging of equid and humanitarian. The stated mission of the AAEP Equitarian Initiative is: “To prepare volunteer veterinarians worldwide to deliver health care and education to improve the health, nutrition, productivity, and welfare of horses, donkeys and mules, and to empower their care providers for sustainable change” [1]. Rather than being a program organized by the AAEP’s leadership, the Equitarian Initiative has truly been a grass roots effort developed by individual equine practitioners with a passion for equine welfare.

In addition to the AAEP Equitarian Initiative, there are other international non-profit organizations, primarily based in Great Britain, that share the mission to provide veterinary care and owner education for working equids worldwide. In fact, many of these international organizations have been operating for more than 50 years and are considerably larger than the AAEP Equitarian Initiative. These include, and are not limited to, World Horse Welfare (www.worldhorsewelfare.org), The Donkey Sanctuary (www.thedonkeysanctuary.org.uk), the Brooke (www.brooke.org), the Society for the Protection of Animals Abroad (www.spana.org), the American Fondouk (www.foundouk.org) and others.

Over a similar time period veterinary education in the developed world has evolved from a focus on teaching basic knowledge and skills required for practitioners to succeed in general practice to increased exposure to advanced medical and surgical techniques common to specialty referral practice. Students attending veterinary colleges in developed countries today receive limited exposure to (and practice on) common medical and surgical problems, leading to a concern that they may be less competent, “practice ready” veterinarians at the time of graduation. This change in veterinary education is well recognized by accrediting veterinary medical organizations including the American Veterinary Medical Association, the Canadian Veterinary Medical Association and the Federation of Veterinarians in Europe [7]. As a consequence, these accrediting organizations have placed increased pressure on US Colleges of Veterinary Medicine to refocus veterinary education to ensure new graduates possess sufficient, day-one competency in general practice knowledge and skills.

When the vast need of working equids for basic care in developing countries is considered alongside the need for educators to provide increased primary care experience for veterinary students, a logical solution was to develop a program of implementing veterinary care for working equids, largely provided by veterinary students under supervision by both educators and experienced equine veterinarians. The purpose of this article is to describe development and implementation of a collaborative project between American and Mexican Veterinary Colleges to accomplish these goals.

## 2. Materials and Methods 

### 2.1. Historical Development of the Collaboration

In 2010 Professor Derek Knottenbelt from the University of Liverpool was invited to Michigan State University’s College of Veterinary Medicine (MSU CVM) as a Freeman Scholar. This honor included delivery of lectures on various topics and one that Dr Knottenbelt shared with the MSU CVM community covered both the need as well as his personal passion and experiences providing veterinary care for working equids around the globe. Following Dr. Knottenbelt’s visit, several faculty members at MSU CVM became interested in developing a program that would combine the need for care of working equids with the need to increase primary care clinical experience for veterinary students in the clinical phase of the curriculum.

Their first step was to attend a special Equitarian Session at the annual AAEP convention and to become involved with the Equitarian Initiative, followed by participation in the 2013 and 2016 AAEP Equitarian Workshops in Tlaxcala, Mexico and Costa Rica’s Osa Peninsula, respectively. The Equitarian Workshops were an annual collaborative effort between the Equitarian Initiative, the AAEP, The Donkey Sanctuary and World Horse Welfare during which a small group of equine veterinarians and animal scientists provide focused instruction about developing realistic equine veterinary care projects in rural settings. Further, the practical aspects of developing international equine welfare projects, ranging from building effective teams to developing lists of supplies and equipment needed, along with clearing Customs, were also discussed. Two days of these “how to” lectures and discussions were then followed by several days of real world equid care, where the challenges of team organization and working under unpleasant conditions (heat, humidity, and biting insects) were experienced first-hand.

### 2.2. Building the Team and Organizing the Project

An important take home message from the Equitarian Workshops was the importance of developing relationships with veterinarians and, possibly, government officials in the host country who would welcome a team, composed largely of US veterinarians and students, to ensure access to communities with working equids in need of care as well as to provide safe and reliable transport and lodging during the project. Between the 2013 and 2016 workshops, MSU veterinarians considered several potential work sites around the world, but fairly quickly returned their focus to Mexico both due to proximity and its large population of working equids [3,4]. In addition, MSU’s College of Osteopathic Medicine had partnered with Universidad Autónoma de Yucatán (UADY) to develop a satellite medical clinic in Mérida, Mexico. Ideally, MSU veterinarians would have combined forces with UADY and MSU’s Mérida clinic to use as a base site from which to develop and implement a working equid project in the Yucatan area surrounding Mérida. However, MSU veterinarians had no contacts at UADY and consultation between MSU veterinarians and their primary contact made at the 2013 Tlaxcala Equitarian Workshop (MVZ Hernández Gil, an equine veterinarian and faculty member at Universidad Nacional Autónoma de México (UNAM)) revealed that the population of working equids in the Mérida region was low and would unlikely be able to sustain a working equid project.

In addition to identifying a region in Mexico where a project could be pursued, MSU veterinarians were only interested if the project could also provide an opportunity for MSU veterinary students to work side-by-side with Mexican veterinarians and veterinary students. In addition to furthering the cultural experience for MSU students of working in a foreign country, sharing the experience with Mexican veterinary students would foster understanding of the similarities and differences in veterinary education and career opportunities in their two countries. 

In the fall of 2016, Drs Schott and Petersen attended the 38th Congress of the Asociación Mexicana de Médicos Veterinarios Especialistas en Equinos (AMMVEE, the Mexican equivalent of the annual AAEP Convention) in Queretaro, Mexico, where they renewed the relationship with MVZ Hernández Gil and met additional Mexican veterinarians, including several veterinarians employed by the Mexican branch of The Donkey Sanctuary (TDS). In addition to the Facultad de Medicina Veterinaria y Zootecnia (FMVZ) and a veterinary teaching hospital, the central UNAM campus in Mexico City also houses the main office for TDS in Mexico. UNAM also has seven satellite research stations or “ranches” around the country that have dormitory facilities for temporary housing of veterinary students and other visitors that work with various production animal species at these stations. Several of the ranches also have a TDS office, typically staffed by a single Donkey Sanctuary veterinarian. This veterinarian routinely visits surrounding rural communities with UNAM and other college’s veterinary students completing their social service programs during which they provide education and service to develop sustainable community animal health systems.

During the AMMVEE Congress, Dr Schott visited the Queretaro UNAM research station as a potential site for development of a collaborative project. Following the AMMVEE Congress, Drs Schott and Petersen drove with MVZ Hernández Gil to Veracruz state on the eastern seaboard and visited the UNAM facilities at the Centro de Enseñanza, Investigación, y Extensión en Ganadería Tropical (CEIEGT, or Veracruz UNAM Ranch). After spending the night at the CEIEGT dormitory, this group visited a few local communities that were reliant on equids for various types of work and transportation and agreed that Veracruz appeared to be a better region than Queretaro for development of the project. On the return drive to Mexico City, a time period (two week semester break for UNAM in late January) was identified, a list of objectives for the clerkship was developed (Table 1), and a decision to start in 2017 was made. MSU veterinarians were responsible for raising funds to support travel, lodging and meals for all participants in the clerkship (both US and Mexican), obtaining consumable supplies (vaccines and sedatives), bringing equipment and initiating a Memorandum of Agreement between MSU and UNAM. UNAM faculty were responsible for organizing vans and drivers for transport, identifying communities to be visited, regional lodging, and providing additional medications, equipment and consumable supplies. 

### 2.3. Development of the MSU Course

In the fall of 2016, a new three week, three credit clinical clerkship for 3rd and 4th year veterinary students, with a maximum enrollment of eight students, was developed at MSU (LCS 613b Equine Welfare in Practice Clerkship). Enrollment in the clerkship required instructor approval. The clerkship included a two week experience in Mexico (an initial two days of lecture and laboratories followed by eight days of work in rural communities), followed by a final week back at MSU where the experience would be discussed and a report and presentation prepared and delivered to the MSU CVM community. At the same time, UNAM faculty also identified a group of veterinary students and equine interns and residents to participate in the project. Jointly, MSU and UNAM organized a series of introductory lectures (scope of the project, role of working equids in rural communities, role of TDS in providing care to working equids and medical and surgical problems expected to be encountered) and field lab exercises (physical examination; differences in behavior and handling of horses, donkeys, and mules; lameness evaluation; farrier work; and dental examination and floating procedures) that would comprise the initial two days of the project. These preparation days were followed by work days in rural communities, with a rest and travel day during the intervening weekend. At the end of the clerkship, the Michigan participants returned to Mexico City where they received a tour of the UNAM FMVZ and veterinary teaching hospital, as well as a tour of the greater UNAM campus. On the final day the group spent the morning discussing and reflecting on the overall experience and provided suggestions for improvement in subsequent years. This was followed by an afternoon visit to the Zócalo or central square in downtown Mexico City and an early morning return flight to Michigan the following day.

### 2.4. Daily Community Work

For each day of community work, the team usually arrived at the working area (a community area such as a soccer field or a ganaderia (cattle yard or stockyard)) to find animals requiring care. Supplies and equipment would be unloaded from the vans and safe work areas for general anesthesia for surgical procedures, farrier work (preferring shade under a tree) and dentistry (often using a tree branch or a soccer goal frame to elevate the head with a rope) were identified. An admission/triage area would be set up and teams of a US and Mexican veterinary student/intern would perform examinations and administer vaccines (a combined tetanus toxoid and multivalent vaccine for equine encephalitides and a rabies vaccine) and an anthelmintic (oral ivermectin solution designed for cattle). Under the supervision of MSU, UNAM, UV and TDS veterinarians, student teams would also make recommendations for further care including farrier and/or dental procedures, wound care and surgery (castration, sarcoid removal and others). Owners would be directed with their animal to the appropriate station for further care.

A simple Excel based form, developed by UNAM and TDS veterinarians, was used to record animals cared for and procedures performed each day. The date and community name were recorded along with the name of each animal and its owner (many working equids remain unnamed). Signalment, including equid type (donkey, horse, or mule), sex, estimated age (known by owner or estimated by examiner) and girth circumference and torso length (from point of shoulder to point of the ipsilateral tuber ischium) measurements were recorded in a single row of the printed Excel form. Circumference and torso length were used to estimate body weight, using established formulas [8]. Additional information recorded was based on the hand model developed by TDS and included assessment of behavior, body condition, wounds, gait and other possible problems [9]. At the end of each day, data recorded on the admission sheets were entered into a laptop computer master Excel file. In addition, a blank sheet of paper was used at each work station to record all surgical, farrier and dental procedures performed. A challenging aspect of this recording system was maintaining accurate counts of all procedures (surgeries, hoof trims, dental floats and others) that were performed over the course of the day as the sheets at individual stations were not consistently filled out. For each day of the project, a team of students was also charged with writing a blog summary of the day’s events along with their personal reflections on their experience. The blogs were collated into a single summary document at the end of the project and sent to donors as well as placed on the web for any interested person to review, (Supplementary Materials).

### 2.5. Evolution of the Project from 2017 to 2019

The initial 2017 project was primarily organized by MSU (Drs Schott and Petersen) and UNAM (MVZs Hernándes-Gil and Garcia-Seco) veterinarians, along with veterinarians and staff with TDS. The Michigan contingent flew into Mexico City where they were met by their Mexican colleagues and driven to the UNAM Veracruz Ranch, in the northern part of Veracruz state. Lodging for the initial week was at the UNAM Veracruz Ranch, followed by a weekend in Veracruz city where the group stayed at a hotel and had time to recover and visit the San Juan de Ulúa Fort and the Veracruz Aquarium. For the initial week, Dr Jeff Bunn, a private equine practitioner from Lowell, MI, also joined the team. During the second week the team travelled west toward Mexico City staying in hotels in Xalapa and Apicazo for two nights each, while working in communities near these cities. The project concluded with tours of UNAM and Mexico City.

In 2018, MVZs Estada-Coates and Alva-Trujillo, veterinarians at Universidad Veracruzana (UV), became the primary organizers of the collaborative project while UNAM colleagues (MVZs Hernándes-Gil and Garcia-Seco) were only able to join the team for two days of community work. For 2018, the Michigan participants flew directly into Veracruz city and the initial two days of lecture and laboratory exercises were held at the new UV Equine Hospital. On the third day the team drove about 3 hours south to the Estación de Biología Tropical Los Tuxtlas, a UNAM Biological Research Station in the rainforest of the Los Tuxtlas region. We stayed in the dormitory buildings at Los Tuxtlas for one week and breakfast and dinner were served daily in the cafeteria, saving both time and expense of having meals at restaurants. Most of the communities visited in southern Veracruz state were near the coastline and, as may be expected in the rainforest, rain showers were frequent. Medical records and blogs were completed as in 2017 with the addition of an interview of a leader in each of the communities. The purpose of the interviews was to learn about the numbers of equids in each community and what type of work the equids performed. Towards the end of the second week we returned to Veracruz city and worked in one local community before driving to Mexico City for the UNAM and Zócalo tours as in the previous year.

For 2019, MVZs Estada-Coates and Alva-Trujillo at UV were again the primary organizers for the project and a Memorandum of Agreement was also initiated between MSU and UV. This year, four MSU veterinarians (three faculty and a surgical resident) accompanied the eight veterinary students and they were also joined by Dr Matthew Davis, an equine practitioner from Cement City, MI, for the initial week and Dr Jeff Bunn for the second week. Dr Lauren Fischer, a recent MSU graduate practicing in Cleveland, OH, also joined the group for the entire two weeks and she was supported by a grant procured by MSU from the Michigan Animal Health Foundation (MAHF) of the Michigan Veterinary Medical Association. Strong winds from an El Norte storm prevented the evening flight from Mexico City from landing in Veracruz so the Michigan participants returned for a short overnight stay in the Mexico City Airport terminal. After safely landing in Veracruz the following morning, the travelers recovered and had the afternoon to tour Veracruz. The orientation program at the UV Equine Hospital was shortened to a half day and the group again headed south to the UNAM Los Tuxtlas Biological Station in the afternoon where they stayed for the next eight days. Shortening the orientation allowed increasing the number of communities visited to 10 this year. Medical records, community interviews and blogs were completed as in the previous year. For 2019, several morning rounds sessions were added after breakfast and before departing in the vans to review more detailed physical examination of specific body systems, dental anatomy and procedures and castration complications. Towards the end of the second week the team returned to Veracruz where they stayed in a hotel and worked in three local communities. On the final weekend, the group again drove to Mexico City for the UNAM and Zócalo tours as in the previous years.

### 2.6. Safety during the Project

Safety of veterinary students was a paramount consideration during development of this collaboration. First, all MSU students that participated were adults, at least 21 years of age, and they had to show that their passports were both current and valid for a minimum of 6 months following the return flight date. Second, MSU students were registered as travelers to Mexico by the MSU International Programs Office, allowing text and e-mail notifications and updates of potential travel and safety concerns (e.g., a magnitude 6.6 earthquake occurred on 1 February 2019 in the southern state of Chaipas during our final day of community work in 2019). Further, all MSU veterinarians and students were issued international travel insurance ($100 USD fee per traveler), with coverage for emergency evacuation if needed should an accident occur. Third, all ground transportation was performed in UNAM, TDS and UV vans and trucks, driven by a Mexican driver. Occasionally, licensed taxis were also used for transport of smaller groups. Fourth, although cellular telephone service was inconsistent in rural communities, a group text was set up for group communications. Fifth, for out of hours activities (e.g., morning exercise or evening walks) students were instructed to travel in groups of at least two students, preferably in larger groups, and to let others know where they would be going and when they were expected to return. Finally, first aid supplies were readily available as were medications for possible gastrointestinal disease associated with travel to a foreign country.

## 3. Results

### 3.1. Communities Visited, Equids Examined and Procedures Performed

In 2017, eight communities were visited and care was provided to 819 equids. In 2018, nine communities (no repeat communities from 2017) were visited and care was provided to 532 equids. In 2019, 10 communities were visited (one repeat community from 2017 and two repeat communities from 2018) and care was provided to 894 equids. Further details about the types of working equids cared for and the procedures performed are detailed in Table 2. In addition to these procedures, many harness wounds and injuries were treated and a number of sarcoids were surgically removed. Interesting maladies including vampire bat bites, tick infestations, chronic airway disease, colic and various eye injuries were also seen. Further, more unusual cases including anemia (likely due to piroplasmosis), nuchal bursitis, cervical vertebral malformation, suspected vesicular stomatitis and tetanus were observed.

### 3.2. Student Participation, Performance and Feedback

During the community visits US/Mexican student pairs were loosely assigned to various procedure stations following the admission/triage process. The goal was to ensure that all students had access to and performed all procedures with special attention paid to rotation through the surgical station for equal sharing of castrations. A lack of a rigid assignment schedule allowed students to spend greater amounts of time at certain stations where they wanted more experience (farrier or dentistry work). Supervising veterinarians also rotated through some of the stations, although there was limited rotation for anesthesia and surgery. This allowed the students to work one-on-one with several instructors to learn various approaches to restraint, examination, and farrier and dental procedures. The students worked diligently during the clerkship and by the end of each day students were fatigued. Often the community would reward the group with a warm meal, in appreciation for the services provided. 

As the number of procedures completed testifies, there was plenty of work to do and all students in the clerkship gained considerable experience and confidence with primary care medicine. Although formal assessment of their improvement was not performed, increased confidence with equid handling, physical examination and administration of vaccines and anthelmintics was clearly evident. As expected, students were more timid at the outset of community work but two factors contributed to rapid improvement: (1) realization that a large number of equids needed to be cared for each day; and (2) personal recognition that their abilities improved rather quickly when they jumped in to do the work. It was fascinating to watch students develop an understanding of how effective animal interaction and restraint may require different approaches. For example, when they first saw a weanling choked down and collapse to the ground after being roped around the neck, they were startled. However, within a few days they were waiting with vaccines and the anthelmintic and learned how to kneel on the upper neck while quickly and safely administering treatments and loosening the rope. Students were given progressively more responsibility for decision making and completion of procedures throughout the clerkship. As an example, for their initial castration they were expected to calculate initial doses of sedation and anesthetic drugs and then were guided through castration of one testicle. As their experience increased, they became responsible for administering pre- and anesthetic drugs and then holding the horse by the halter to guide it to the ground as anesthesia was induced. As the anesthetist, they were given the decision making power of whether or not more drugs may be needed and they also guided the equid through the recovery process. When their role was as the surgeon, over time they were expected to complete the procedure with less and less instruction, although a non-gloved surgeon was observing and available to answer questions. A similar progressive increase in independence was provided for performance of farrier and dental procedures, although supervision was always close by. On return to MSU at the end of the clerkship, the students have become the best advocates of the clinical experience. After they talk about their experiences with classmates, the subsequent year’s roster fills quickly.

The students are asked to complete a faculty (veterinarian) and clerkship evaluation form during the final week of the clerkship after their return to Michigan. The comments have been unanimously favorable about the opportunity for hands-on practice. Further, the students have been very positive about the cultural experience, including working with their Mexican peers and seeing the UV Equine Hospital and the UNAM FMVZ and veterinary teaching hospital. There have been no language skill prerequisites for the clerkship; however, the students with some ability with Spanish did seem to have a more fulfilling experience. Essentially all of the students have commented that they wish they would have tried more to learn some Spanish before the clerkship. At MSU, students are encouraged to pursue this goal once they register for the clerkship; unfortunately, a short course for learning Spanish for students to complete prior to the clerkship is not readily available at MSU. 

### 3.3. Resources Procured

The collaborative project started as an idea, without any specific financial support. The goal was to use existing resources to the greatest extent possible. Vans and equipment from UNAM, UV and TDS were generously shared without usage fees charged. Additional equipment was transported to Mexico from MSU, including dentistry equipment loaned to the project from a private MI veterinarian. Vaccines were donated by Boehringer-Ingelheim, Vetmedica and Merck and detomidine was donated by Zoetis: these supplies were greatly appreciated. The clerkship also received limited financial support from the MSU CVM International Programs Office. However, the majority of financial support was and continues to be obtained through private donations from clients of MSU’s Large Animal Hospital. Further, in 2019 a grant was funded by the MAHF to provide support for a recent graduate to accompany the team, as a recent graduate would likely have loans that would preclude their ability to support travel and time away from their practice positions. In 2017, MSU, UNAM and TDS shared the costs of gasoline, medications, and meals and the group was fortunate that UNAM charged only a token amount for lodging in their dormitories. Over time, the goal was for MSU to be able to provide the funding for all expenses in Mexico while UNAM, UV and TDS would continue to supply vans, equipment, and personnel at no cost to the project. For 2017 and 2018, MSU students were only required to pay the cost of their airfare but fundraising success allowed this cost to be covered by the clerkship in 2019. All in all, expendables by MSU were about $7500 in 2017, $9000 in 2018, and $12,000 in 2019. The increase is largely attributed to MSU raising enough funds to be able to pay for all lodging, meals, gasoline and medications for all participants by the 2019 project. It warrants emphasis that a cost of $12,000 for students to work with nearly 900 equids and have a tremendous cultural experience represents a relatively low cost for veterinary education.

## 4. Discussion

Successful development of the MSU-UNAM-UV Equine Welfare in Practice Clerkship required vision, learning, relationship building, creativity, fund-raising and perseverance. From the start the vision was a project focused on veterinary student training through immersive, hands-on experiential learning. Although MSU veterinarians also considered developing a primary care student experience in the US by joining current projects on Indian reservations or by offering veterinary services to Michigan horse owners under financial distress (job loss or other unexpected loss of support for caring for their horses), they also wanted the project to be an eye-opening experience for veterinary students to understand the importance of working equids around the globe. This required reaching beyond the borders of the US, and Mexico was a logical choice due to the large number of working equids and proximity to the US. Another important part of the vision was to develop a program in which US veterinary students could work closely with Mexican veterinary students, to both experience another culture as well as to understand the similarities and differences in veterinary training they receive. This required that the project be a collaborative effort between US and Mexican veterinary schools.

Next, MSU veterinarians had to learn about resources and supplies needed for the project, as well as logistics required for transporting both a group of US students and medical equipment and supplies into Mexico. Fortunately, attending AAEP Equitarian Workshops provided excellent training and allowed introductions to future collaborators. Although the donated supplies from pharmaceutical companies were quite useful, MSU veterinarians had to learn which supplies could be cleared through Customs in Mexico. Although letters of invitation from UNAM and UV collaborators shared with Customs Officials were helpful, MSU veterinarians did not have any documents from the Mexican government approving a list of supplies that we could bring into Mexico. Mexican Customs officials had few concerns about dental, farrier and surgery equipment; however, syringes and needles should not be transported into Mexico (1000 were confiscated in 2017). Further, because not all bags/backpacks are examined at Customs, distributing supplies between all travelers (e.g., bottles of detomidine and packs of suture material) allowed a greater chance that supplies would be allowed to pass through Customs, as officials’ search methods and concerns about medications and supplies varied from year to year. In 2017, flight delays also resulted in failure of luggage arriving into Mexico City for three MSU students and they were without a change of clothes for the initial three days of the clerkship. Subsequent to this experience, travelers from the US are encouraged to carry as much clothing and other necessary supplies in a backpack or other small bag that can travel as carry-on bags (also saves on luggage fees). Although these details may not all seem worthy of inclusion in this manuscript, they are described in order to assist others that may want to pursue development of similar projects in the future. 

The importance of relationship building cannot be overemphasized. MSU veterinarians travelled to Mexico three times before the initial 2017 clerkship to meet and work with UNAM and UV faculty to develop the collaborative project. These face-to-face visits were essential to develop common objectives and goals, to see where MSU veterinarians would be bringing MSU students (important to alleviate security concerns about transport and lodging in Mexico), and to discuss and decide on what equipment and supplies would be needed. Further, development of relationships allowed the MSU, UNAM and UV veterinarians to clearly understand expectations of each other during the actual project.

Although there were several models from the Equitarian Initiative Workshops and other successful equitarian projects to follow, creativity and brain-storming were important aspects of developing our program. This started with gathering information about what resources each partner could bring to the table. The UNAM, UV and Donkey Sanctuary partners were able to provide veterinarians, vans in current use for equid veterinary care in rural communities and additional vehicles and drivers for transporting the group. The Mexican partners were also able to organize safe and affordable lodging. Most importantly, the Mexican partners had developed relationships with community leaders that were critically important in both identifying communities reliant on working equids and for which veterinary care was unavailable (the team did not want to compete with local veterinarians). Organizing the communities typically required a visit to each community by a UNAM, UV, or TDS partner within the month before the clerkship started. This represented a substantial time investment by the Mexican partners and the clerkship could not have been successful without these efforts. The MSU veterinarians were responsible for bringing additional equipment, obtaining donated medical supplies and raising funds for the majority of the expenses incurred (van drivers, lodging, gasoline, meals, medications, etc.) during the clerkship. The MSU group was also responsible for collating data on equids examined and procedures performed, as well as for preparing summative reports following the clerkship.

Funds for the clerkship were raised from several sources. For 2017, all funding was raised from donors to MSU, including faculty participants in the clerkship and clients of the MSU Veterinary Medical Center. In 2018, private donations remained the primary source of funding and the MSU CVM International Programs Office provided additional funding. In 2018, two grant proposals were also submitted to the AAEP and the MAHF, one was funded by the MAHF for $5000 to support travel and participation by a recent graduate of MSU CVM, as participation by a recent graduate would likely be difficult due to both work and debt repayment obligations. In 2019, funding included private donors, additional funds from the MSU CVM International Programs Office and the award from MAHF. To ensure ongoing funding from private donors, all donors receive a thank you card from the students as well as a copy of the blog prepared by the students. As donors have seen the positive impact of their donations on veterinary student development, the majority have continued to provide further support on an annual basis.

Lastly, development of the MSU-UNAM-UV Equine Welfare in Practice Clerkship required perseverance. After returning from the 2013 Equitarian Initiative Workshop in Mexico, the MSU veterinarians that participated presented their idea of developing an MSU CVM clerkship to the upper administration of MSU CVM. We were met with lukewarm enthusiasm, at best, and received little support or encouragement to proceed forward. Nevertheless, we remained positive and enthusiastic, largely due to motivation originally instilled in us during Prof Knottenbelts’s 2010 visit to MSU. Over the next couple of years, MSU CVM upper administration changes resulted in a new Dean and Large Animal Clinical Sciences Department Chair that were more supportive of the concept and approved the new clerkship offering. Once the 2017 clerkship was completed and the positive outcome was apparent, the MSU CVM International Programs Office became involved with both financial and administrative support.

Although the current clerkship has largely been focused on providing primary care experience for MSU and Mexican veterinary students, with additional benefits to working equids and their owners, a goal is to expand the impact of the project in future years. Specifically, further investigation of medical problems that affect working equids in these rural Mexican communities will be pursued. Previous studies by UNAM veterinarians and students have documented internal parasite burdens [10] and prevalence of dental disorders in working equids [11]. Similarly, work by UV veterinarians and students has found high infection rates in Veracruz communities with Equine Infectious Anemia virus (33.5%, increasing with increasing age [12]) and agents causing piroplasmosis (74.4% positive for *Theleria equi*, 57.4% positive for *Babesia caballi*, with 47.5% of working equids having mixed infections with both hemoprotozoa [13]). During the 2019 clerkship blood samples were collected from several 100 equids for testing for antibody titers to *Leptospira* spp., a potential zoonotic disease. Areas for future study could include characterization of tick infestations and infectious diseases they may transmit, diseases carried by vampire bats and possible control programs to limit vampire bat bites, and antibody titers in response to vaccinations. Under the One Health umbrella, these investigations could extend to investing additional zoonotic diseases including rabies, brucellosis and cutaneous tuberculosis [14]. To complete these studies both funding and students will be needed. Fortunately, the social service program requirement during the final years of Mexican veterinary students’ course of study could provide students to assist with these projects. This veterinary education model requiring a social service component may be worthy of consideration by US veterinary schools, although the difference in tuition models between veterinary education in both countries would need to be taken into consideration. 

Further, evidence-based assessment of the impact of veterinary care of working equids and owner education on equid and community welfare would be another One Health goal [15]. As an example, veterinary students currently receive minimal education about harnesses, saddles and bridles that may be used on either working equids or performance horses. Development of community-based research projects, utilizing novel design of these materials, involving both veterinarians practicing in rural communities and veterinary students could document whether or not changes in harness and saddle design, using simple assessment tools, may have a favorable impact on equid welfare. Improved welfare could allow an increased amount of work to be performed by these animals, thereby increasing productivity of individual families and entire communities.

To provide further educational opportunities, exchange programs between US and Mexican students, interns, and residents for periods of study at MSU, UNAM, UV, and clinical experience with private equine practitioners in Michigan and Mexico may also be developed. However, because limited language skills remain a major impediment to learning in a foreign country, students should first develop bilingual language competency and efforts are needed to provide this opportunity in both countries. Finally, a long-term goal is the hope that exposing veterinary students to the vast needs of working equids and their owners will instill a passion for further volunteerism, in order to ensure provision of veterinary care to these animals by the next generation of veterinarians being trained in both countries.

## 5. Conclusions

Development of the collaborative MSU-UNAM-UV Equine Welfare in Practice Clerkship required vision, learning, relationship building, creativity, fund-raising and perseverance to develop and agree on mutually beneficial objectives for all participants. The project is largely financed through private donations and supplies provided by pharmaceutical companies. The outcome has been a highly successful program that could be used as a model by Colleges of Veterinary Medicine world-wide.

## Figures and Tables

**Table 1:** Faculty and student objectives for the Equine Welfare in Practice Clerkship.

**Veterinarian (Educator) Objectives:** To develop
1. a joint educational program for MSU, UNAM and UV students in the clinical phase of veterinary training, as well as trainees in internship and residency programs, that will allow them to acquire an understanding of the need for welfare of working equids, along with development of clinical skills and to provide management recommendations and medical and surgical care to working equids;
2. collaborative, hypothesis-driven research studies, using a One Health perspective, designed to further knowledge of best practices in provision of care of working equids to improve economic status and family welfare; and
3. faculty and student, intern, and resident exchange programs between the institutions to further collaboration and, ultimately, knowledge for provision of appropriate management and health care welfare to working equids.
**Student Objectives:** To develop
1. an understanding of the vast need for care of working equids in Mexico (and the rest of the world);
2. an understanding of the importance of working equids to the welfare of families living in poverty;
3. an understanding of the importance of adequate nutrition to perform work and reproduction;
4. practical skills with preventive medical care, including disease prevention;
5. enhanced skills in history taking and clinical examination, in order to determine appropriate and economic approaches to clinical problems (i.e., clinical reasoning and problem solving);
6. practical skills with harness and saddle fitting, including wound care associated with harness and other injuries;
7. practical skills with routine dental care;
8. practical skills with routine hoof care;
9. increased skills with routine surgical procedures including castration and more advanced wound care requiring surgical debridement; and
10. a lifelong passion for and participation in supporting welfare of working equids.

**Table 2:** Numbers of equids for which care was provided and procedures performed from 2017 to 2019.

	2017	2018	2019	Totals
**Equids**				
Horses	335	445	482	1262
Burros	382	64	325	771
Mules	102	23	87	212
Totals	819	532	894	2245
**Procedures**				
Hoof trim	135	62	128	325
Dental float	74	46	102	222
Palpation for pregnancy	29	36	44	109
Castration	20	18	43	81

## Supplementary Materials

The 2017 through 2019 student blogs are provided as Supplementary Materials, which are available online at https://www.mdpi.com/2076-2615/9/4/164/s1.
