# Supplementary material for: Equine Welfare in Practice: A Collaborative Outreach and Education Program with Michigan State University, Universidad Nacional Autónoma de México, and Universidad Veracruzana
Source: Animals (Basel). 2019 Apr 13;9(4):164. doi: 10.3390/ani9040164 (PMC6523699; doi:10.3390/ani9040164)
Supplement: Supplementary file 1 [file animals-09-00164-s001.zip › animals-471606-supplementary/MSU-UNAM-UV Equine Welfare in Practice - blog - 2017.pdf]

Equine Welfare in Practice Blog – Day 1 - Sunday 1/22/17, by Hal Schott

21 hours to get to the Veracruz Ranch - it was not supposed to take this long to travel, that's one of the reasons we chose Mexico over Africa! The early hours of Sunday morning were dense with fog as I slowly started my way around East Lansing at 3 am to pick up Andrea, Lauren, Emily, and Jackie. Then we headed to the Flint Airport a bit slower than usual due to the fog and arrived about 4:45 am for our 6 am flight to Atlanta. Olivia and Alex met us at the airport and Sam and MacKenzie were already in Mexico City after completing another welfare clerkship focused on small animals on the west coast of Mexico (Manzute project). We boarded the plane and sat (and sat) at the end of the runway until they finally unloaded us again about 8:30 due to the fog that refused to lift. Finally, about 9:30 we reboarded the plane and headed to Atlanta. We had missed our original connection but made a mad dash to catch a 12:10 flight and arrived at the gate as boarding was ending. Most of our checked bags made it to Mexico City except for Alex, Lauren, and Emily's bags. While they were sorting out where to have their bags forwarded to, I sent Andrea, Jackie, and Olivia through customs (with some syringes and detomidine tucked into their luggage). I headed to customs inspection after reporting that we were bringing medical equipment into Mexico. The customs official had no problem with vaccines, gloves, or dental and farrier equipment - but 700 needles and syringes to administer the vaccines were confiscated. For some reason the officials had to count them one by one and they messed up the initial count and dumped them all back on the table for a recount, as 45 minutes slipped by. Finally, by about 4:45 pm we had all cleared customs and settled down for a late lunch/early dinner in the airport with our Universidad Nacional Autónomo de México (UNAM) and Donkey Sanctuary colleagues. Then we loaded up in a UNAM van and a couple of cars for the 5 hour trek to the Veracruz UNAM Ranch. But who could complain with nice weather, blue skies, the Iztaccíhuatl and Popocatépetl volcanos in the distance, and a nice sunset looking back towards Mexico City.

Legend has it that Iztaccíhuatl was a princess who fell in love with one of her father's warriors, Popocatépetl. The emperor sent Popocatépetl to war, promising Iztaccíhuatl

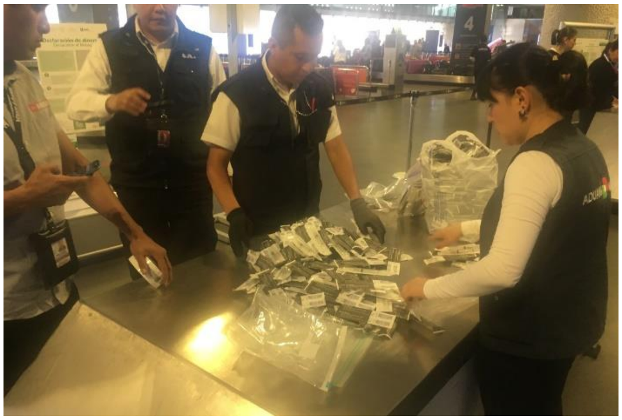

Day 1. Mexico City airport. Customs officials counting syringes one by one prior to confiscating them. Fortunately, all other vaccines, medications, and equipment was passed through Customs without difficulty.

as his wife when he returned (which Iztaccíhuatl's father presumed he would not). Iztaccíhuatl was falsely told Popocatépetl had died in battle, and believing the news, she died of grief. When Popocatépetl returned to find his love dead, he took her body to a spot outside Tenochtitlan and kneeled by her grave. The gods covered them with snow and changed them into mountains. We finally arrived at the Veracruz ranch and settled into our rooms quickly for bed. Despite lost luggage and a long day, there were no complaints as we were enjoying a warm evening and a star filled sky.

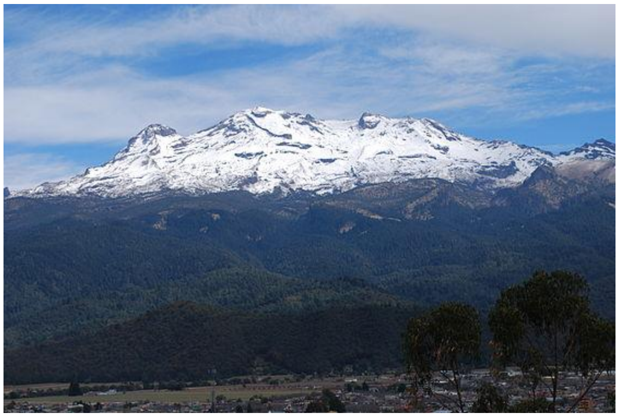

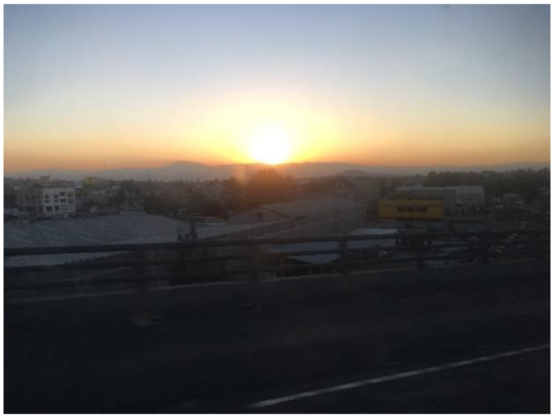

Day 1. Driving out of Mexico City with Iztaccíhuatl, the sleeping princess, on the eastern horizon and the the sunset over Mexico City behind us.

Day 2 – Monday 1-23-2017, by Sam Gamble

Good morning! We woke up this morning to the sounds of local critters (geckos and birds) of the **Centro de Enseñanza, Investigación, y Extensión en Ganadería Tropical (CEIEGT or Veracruz UNAM Ranch)** where we were staying for the initial part of our trip. The ranch was beautiful, with lush green pastures, palm trees, a river, and mountains in the distance. We loaded up into the Donkey Sanctuary vans to head to breakfast at a local restaurant. It was nice sitting down with the Donkey Sanctuary team and the UNAM students and interns and getting to know them. We learned that several students were in the community service portion of their final term, a mandatory part of their training during which they work on a variety of projects, largely in rural communities all over Mexico. Interestingly, some of the Donkey Sanctuary staff were not veterinarians but also included an anthropologist and a sociologist (Humberto and Jose Antonio), emphasizing the holistic philosophy of the Donkey Sanctuary in community service. Everyone at the breakfast table tried to learn a little more about one another, including their interests in school, where they were from, and their experience with equids. It was nice to learn many of the team member's names and get to know them a little bit while enjoying some amazing Mexican food.

Following breakfast we loaded back up into the vans and headed back to the Donkey Sanctuary headquarters and administrative office at the UNAM Ranch where we were

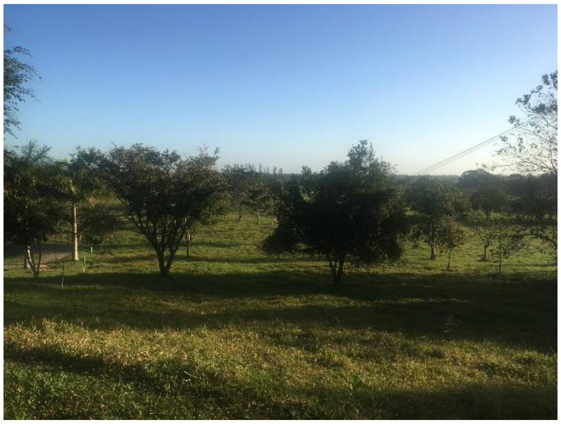 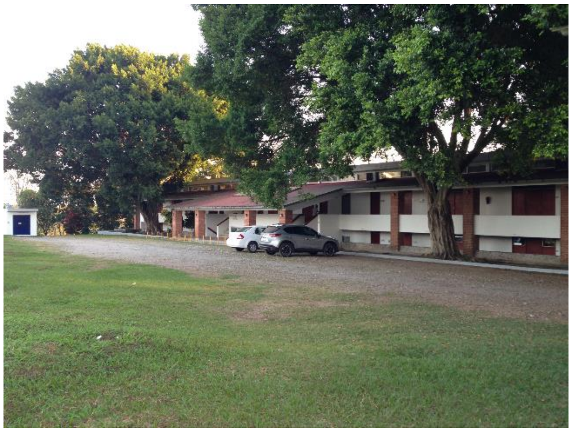

Day 2. Early morning at the Veracruz UNAM Ranch (left) and one of the dormitories (right).

introduced to “The General”, the primary veterinarian in charge at this location and Humberto, the anthropologist who also works in this location. We learned that in each Donkey Sanctuary location there is a team of veterinarians, sociologists, and anthropologists that work together in the communities to not only provide veterinary care, but to really learn from the people in the community about their culture, daily lives, needs, and animal welfare concerns. I think we all found this approach admirable and refreshing that veterinarians and social scientists were working together for the greater good of both the people and their animals within the community. The Donkey Sanctuary staff emphasized the importance of understanding the community in order to provide the best veterinary care possible. The staff also mentioned how they use the headquarters to host international students, interns, and veterinarians from around the world who come to work at the Donkey Sanctuary. The United Kingdom, Nicaragua, and various regions of Mexico were mentioned as examples of countries that have participated. Michigan can now be added to this list!

Following our visit to the Donkey Sanctuary headquarters, we had a tour of the Veracruz UNAM Ranch. It consisted of a number of lush pastures where various breeds of cross-bred Zebu and Fresian cattle were grazing as well as a few horses and sheep. There is also an aquaculture facility here where a UNAM patented tilapia variant is raised for commercial use. The tour also included a stop at a cattle sorting/processing facility that had been designed by Temple Grandin when she visited the ranch over 20 years previously. We ended up at the Education Building where we learned more about our MSU-UNAM Equine Welfare in Practice project from Donkey Sanctuary staff. Once in the classroom we distributed t-shirts that we had made for the project and took the time to go around the room and introduce ourselves. Many of the Donkey Sanctuary staff and the UNAM students could understand English, and some could also speak English fairly well, but we also had Jose Antonio translate which was incredibly useful for the whole team. Many of the UNAM students said they would like to learn more English and the MSU students also said that they would like to learn more Spanish so this project is a great opportunity for everyone. Introductions taught us more about one

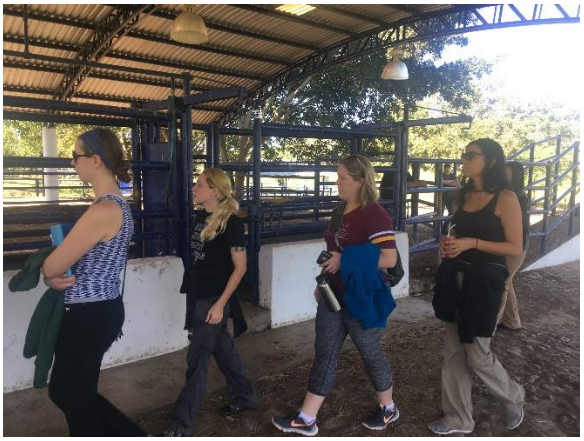

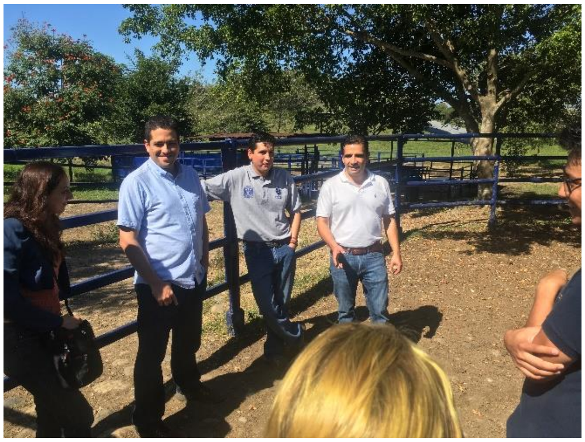

Day 2. Touring the UNAM Ranch: cattle handling facilities designed by Temple Grandin (left) and Donkey Sanctuary veterinarians Drs. Eduardo, Arturo, and Mariano (left to right) talking about the facilities at the Veracruz UNAM Ranch.

another including interests and roles with UNAM and the Donkey Sanctuary, and with a team of almost 30 name tags that were distributed helped as well.

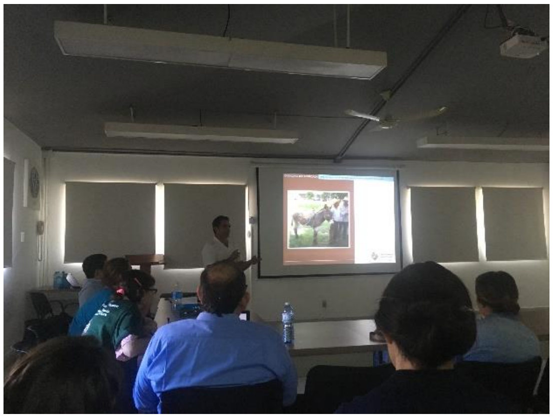

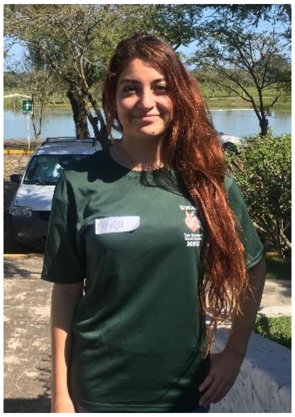

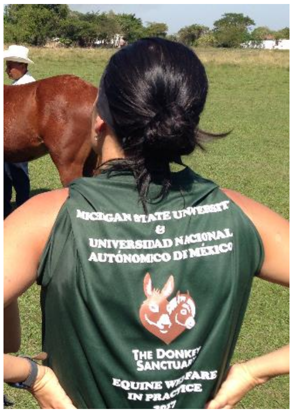

Day 2. Education always starts in the classroom with Dr. Mariano discussing the history and mission of the Donkey Sanctuary (left). Dr. Monica (UNAM intern, middle) and Sam (MSU student, right) model the t-shirts made for the Equine Welfare in Practice 2017 Clerkship

We began the education program by watching a short video in which children from rural communities were interviewed about the role of their working equids. Their answers included carrying loads (water, wood, produce, etc.), transportation, and companionship; one child called his donkey his "best friend." This video truly emphasized the importance of the human-equid relationship in many communities. The first lecture was given by Dr. Mariano Hernadez Gil, a veterinarian from UNAM who provided good information about the roles of working equids in communities. He made a great point about how easy it can be to have "false" perceptions. As an example, he showed a picture of a girl with a stick standing next to her donkey. He mentioned that people may think she used the stick to beat her donkey, but when she was asked why

she had the stick the girl replied that she needed it to keep away dogs that might attack her donkey. This was a great example about the importance of communicating with owners and not jumping to conclusions. Dr. Mariano also mentioned how important educating the community is and that the goal of the Donkey Sanctuary is not only to provide intermittent veterinary care but to also help the community learn how to best manage their working equids for optimal health and productivity.

The next lecture on welfare was given by Dr. Eduardo Santurtun Oliveros. He talked about how welfare has many components, including observation for behavior (including owner interactions), recognition and prevention of pain, and provision of veterinary care. Dr. Eduardo introduced us to a welfare model developed by the Donkey Sanctuary that is represented by a hand. The five fingers represent behavior (including interactions with the owner/handler), body condition, wounds, lameness, and general health (see Figure). Dr. Eduardo also emphasized the importance of understanding the economic status and needs of the animal owners within each community, to tailor education about equine welfare to each community served by the Donkey Sanctuary.

Day 2. The Donkey Sanctuary's "Hands-On" depiction of aspects of welfare that should be addressed when examining working equids.

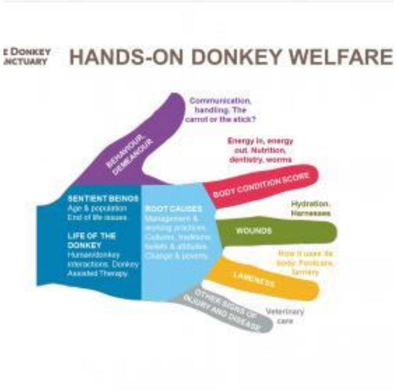

After the lectures, we headed to a local restaurant for a late lunch – carne asada (barbecued beef, pork, and chicken) was served family style with homemade tortillas – all part of the cultural experience of the clerkship!

Day 2. Homemade tortillas were standard fare for most of our meals.

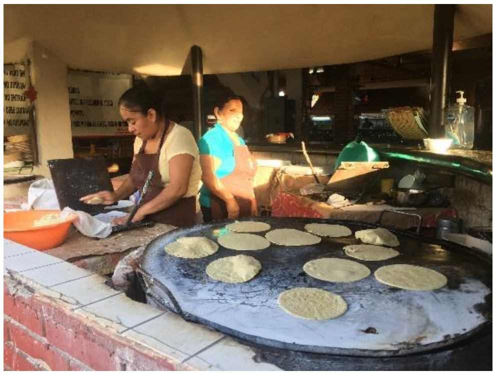

After our meal we headed to a local ranch owned by Reuben Vargus, a friend of Dr. Mariano's. We spent the late afternoon/early evening learning more from the Donkey Sanctuary staff – Mauro, the behavior expert, described the differences in behavior of

donkeys, horses, and mules. We all were somewhat familiar with horse behavior but donkeys and mules are different. Donkeys often refuse to move and ear holds are useless on these critters while mules react with lightning speed and can kick you almost anywhere you stand (just ask Jackie). Mauro then demonstrated how he assesses behavior and interacts with young horses by working with a couple of young horses in a round pen. A couple of cars had to be used to illuminate Mauro and the horses in the headlights and Jose Antonio's (the Donkey Sanctuary sociologist that had a dual role as our primary translator for the project) voice was getting a bit hoarse – both indicators that it was time to call it a day. We headed back to the UNAM ranch for the evening.

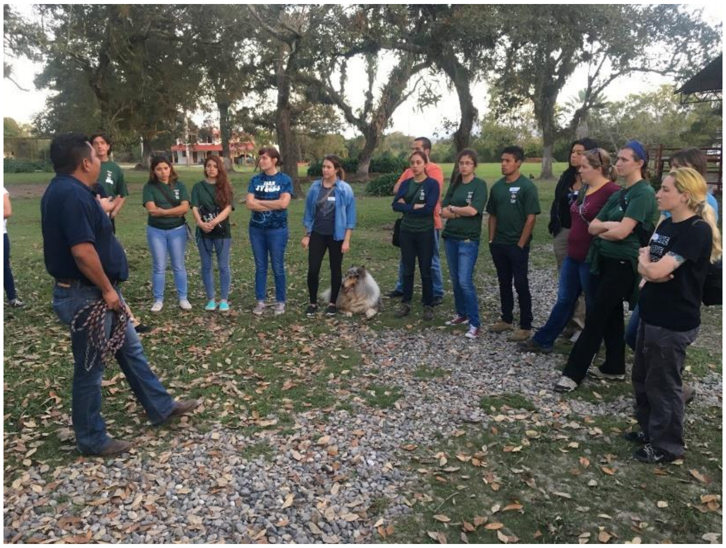

Day 2. Mauro, the Donkey Sanctuary's equine behavior expert talks to our group about the differences in behavior between horses, donkeys, and mules.

Day 3 – Tuesday 1-24-2017, by Jackie Dessoliers

After a group breakfast at what was going to be our breakfast buffet spot for the next few days, we headed back to the Vargus Ranch for a second day of education before we started work in the communities. Dr. Mariano started the day by talking about conformation with a friendly horse and differences between horses, donkeys, and mules. Because of differences in body length between these equid types, UNAM and Donkey Sanctuary staff have developed formulas for estimating body weight in the field using girth circumference and body (shoulder to tuber ischia) length – we decided to further validate these formulas during our community work (see later). Dr. Mariano also emphasized the importance of a complete physical exam, with a particular focus on evaluating potential pain in working equids.

After this demonstration, Luis Aguilar (the Donkey Sanctuary's harness and saddle fitting expert) talked about wounds and other problems that can develop with poor fitting saddles, harnesses, and bits. He emphasized that one saddle or harness does not fit all, especially with the different body conformations of horses, donkeys, and mules. He showed how saddles and harnesses are appropriately fitted to working equids and talked about the impact that different types of work may have (e.g., whether equids are carrying people or loads on their backs or pulling carts). Back pain and wounds over the

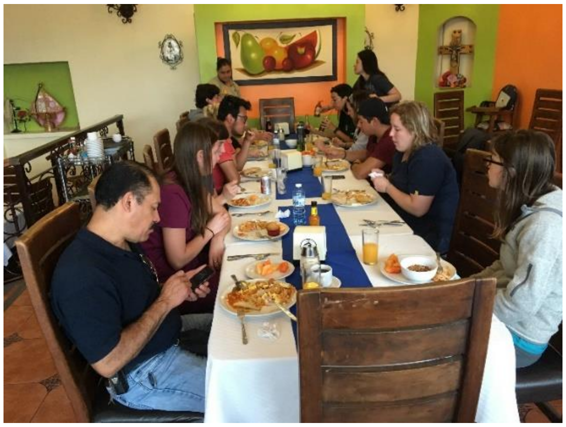

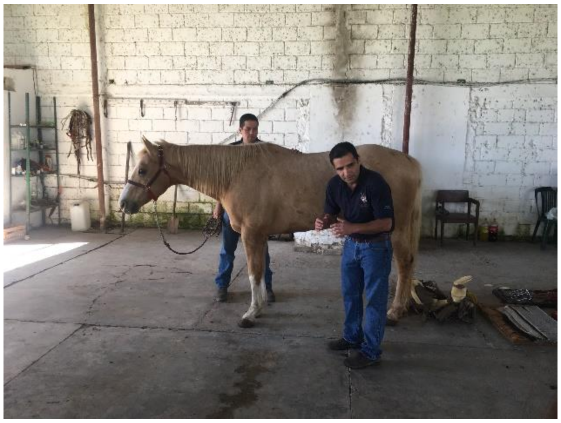

Day 3. Our breakfast spot near the UNAM Ranch (left) and Dr. Mariano talking about conformation of working equids (right).

withers and dorsal spinous processes along the back are common with improper saddle and harness fit while improper bit placement can cause ulcers in the mouth and lacerations of the commissures of the lips or on the tongue. Many working equids have patches of white hair along the withers or back indicative of prior wounds that have healed but these areas may still be painful when pressure is applied. To illustrate some of these problems, Luis made us perform an exercise where students acted as the loaded equids. He helped us understand the importance of distributing weight using a singletree when pulling a load or by dissipating pressure points by distributing loads over a larger surface area. He also told us that many owners misinterpret equids‘ signs of pain during work as “bad” behavior, resulting in a viscous cycle of more discipline (beating with a stick) and further injury. One of Luis’ primary duties is to educate equid owners in communities on how to properly fit saddles, harnesses, and bits to prevent injuries from happening.

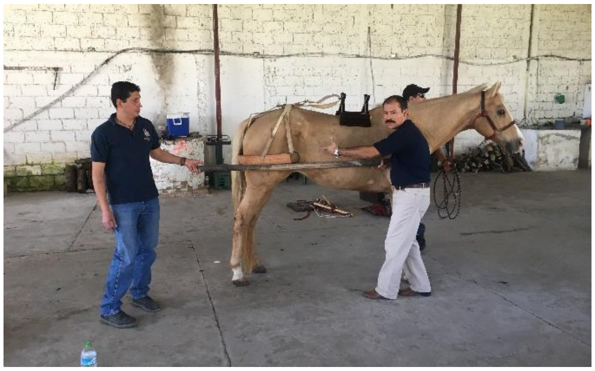

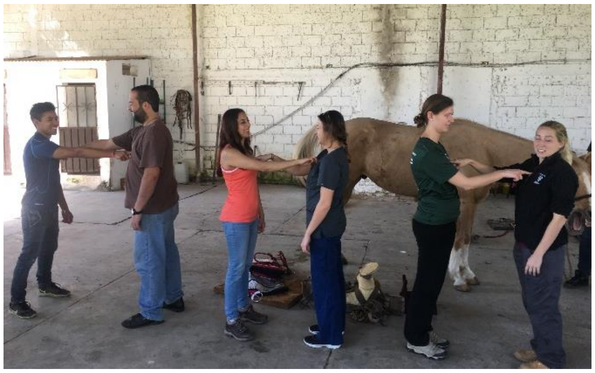

Day 3. Luis demonstrating various harnesses that may be used on working equids (left) and students doing an exercise to show differences in pressure distribution (right)

After Luis’ demonstration Dr. Elena Garcia-Seco, an equine surgeon at UNAM, reviewed assessment of lameness. This was a true “hands on” experience as a load of new saddle horses arrived at Reuben’s ranch in the afternoon. MSU and UNAM interns/students were paired up and each group had a horse to examine. Dr. Elena started the exercise by making them pick up front and hind limbs, palpate the structures

of each limb, apply hoof testers, and watch each horse jog for lameness. It warrants mention that these were not the most broke horses and a couple of times one got loose and galloped around the ranch - fortunately, the ranch was surrounded by a wall. Although we may not have recognized it at the time, this was a good introduction to what we were going to be seeing in some of the communities with working equids over the coming days. All the while we were busy with these exams, we were also distracted by dogs, peacocks, several pigs, and a laminitic horse that were freely roaming through Reuben’s ranch area that we were working in – another welcome to the uncontrollable environments we would be working in over the next days. After we completed these exams without injury (to the horses or students), we had a break and gathered around a couple of tables to devour some great Mexican food provided by Reuben as a thank you.

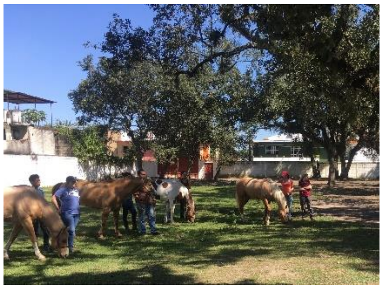
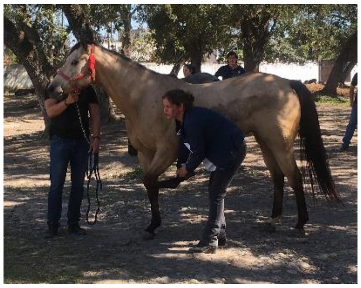
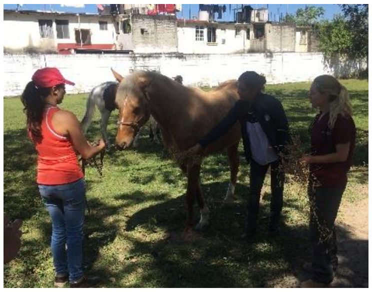

Day 3. Pairing up with our UNAM partners to do “pre-purchase exams” on Reuben’s horses (left) with hands on instruction about palpating limbs and evaluation lameness by Dr. Elena (middle and right)

The next discussion was a review of some infectious diseases that we might possibly encounter (hopefully not) led by Dr. Schott. We reviewed the diseases for which we had brought vaccines for: tetanus, EEE, VEE, WNV, and rabies. The encephalitides (EEE, VEE, WNV, and rabies) are present in Mexico but surveillance programs are not as well developed as in the US; consequently, true prevalence of these disease is not well documented. As our discussion wound down, Reuben had a friend present us with a horse that had collapsed during exercise about 6 months ago. The episode of collapse had occurred on a very hot and humid day and we worked through some differential diagnoses and we were most suspicious of anhidrosis. Luckily, this horse seemed to have recovered after being out of work for the past 6 months as it had a good sweating response while being ridden today. This was a great example of a tropical disorder that we would not likely see back home in Michigan.

In return for Reuben’s generosity in letting us use his ranch and horses as a teaching lab, we spent the rest of the afternoon and early evening hours vaccinating his horses and performing dental exams. We performed dentistry on several animals and as the day was ending, we watched Dr. Arturo trim and bandage the front feet of the laminitic horse that had been wandering the property. About that time we were getting hungry but also noted a few red spots on our exposed arms – we had been attacked by tiny mosquitos or chiggers (we were never sure) and would be very itchy over the next few

days. Oh well, just another surprise of the tropics. Before we headed to dinner, we had to pick up Dr. Jeff Bunn (Equine Medical, LLC in Lowell, MI) who had flown into Veracruz late that afternoon to join our group. Dr. Bunn was very concerned about whether or not there would be transport at the airport and, after several last minute changes and frantic texts and phone calls, a professor from the veterinary school in Veracruz met Dr. Bunn and then delivered him to Pepe in Veracruz city, a taxi driver to drive him 2-3 hours to meet up with us. Fortunately, it all worked out and we retrieved Dr. Bunn from Pepe at the Pemex gas station in Martinez about 9 pm. It was serendipity that Dr. Bunn joined our project as he had recently taken a couple of Spanish classes to help him communicate with grooms on several of the horse farms in his practice. When Dr. Schott sent an e-mail inviting Michigan practitioners to consider joining the project, Dr. Bunn jumped at the chance (perhaps without thinking it through completely). He was certainly happy to see us pull into the Pemex station that evening and then we all headed to a simple but great local restaurant for traditional Mexican food and cervezas.

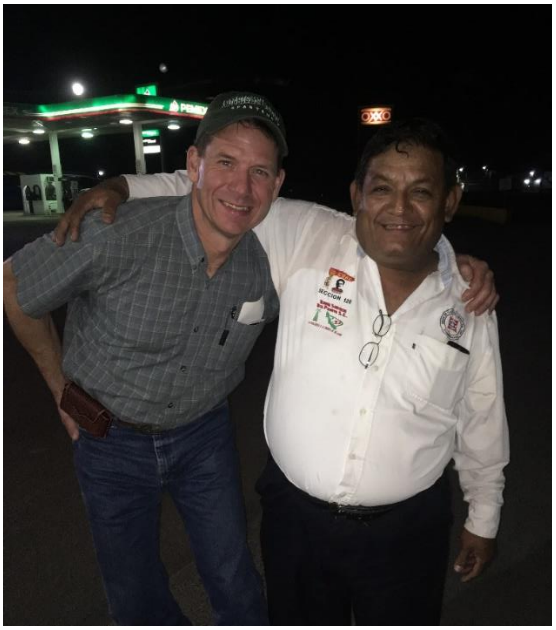

Day 2. Dr. Jeff Bunn, transported to the Pemex station in Martinez de la Torre by Pepe. Dr. Bunn was glad to see that we had not forgotten him!

Day 3 – Wednesday 1-25-2017, by Andrea Mussara

Today was the first day of field work. After breakfast we drove about 2 hours to the community of San Sebastián, Veracruz. When we arrived there were many equids waiting for us. After a short hands on demonstration of the evaluation process by Dr. Eduardo and the General, we divided into stations and started evaluating the animals. Sociologists/anthropologists with the Donkey Sanctuary were also on site to help the equid owners understand how improving the welfare and health of their animals can benefit the family economically. The first station involved evaluating the health and welfare of the animals and performing a physical exam, using the Donkey Sanctuary hand model. Based on the results of this initial evaluation, vaccinations and dewormer were administered and other procedures were recommended as necessary with

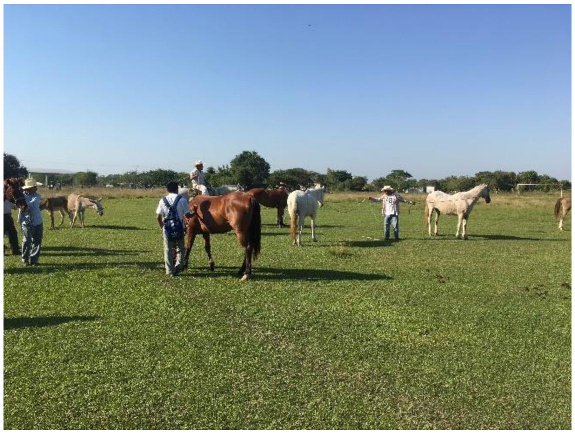 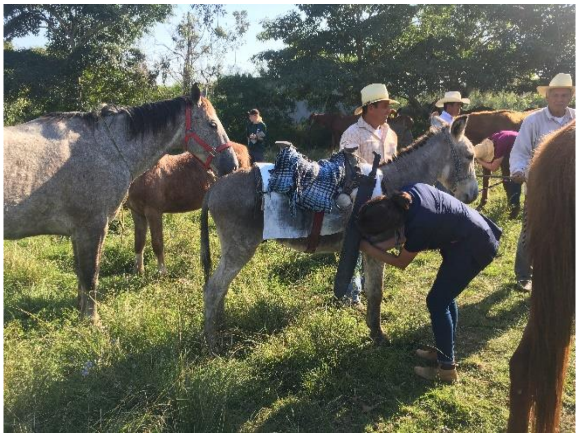

Day 3. On our arrival at the San Sebastian work site, many animals and their owners were already waiting for us. Notice in the right hand photo the unique saddle with two horns, used to attach jugs of water or milk for transport – a long machete is also a feature of almost every saddle or harness on these working equids.

animals sent to a dental care station, harness fitting station, farrier station, and/or castration station depending on the equids' needs. Each station was overseen by a veterinarian and had at least two veterinary students, one from Michigan State and one from UNAM.

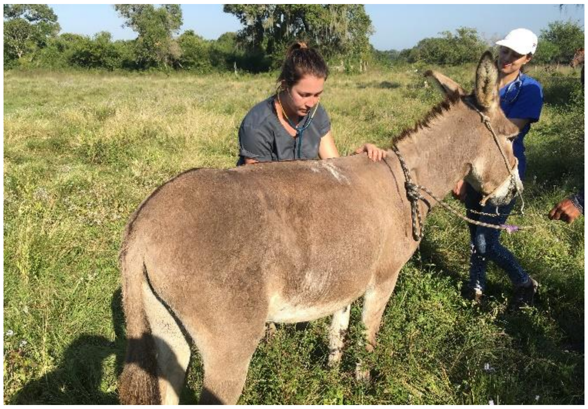 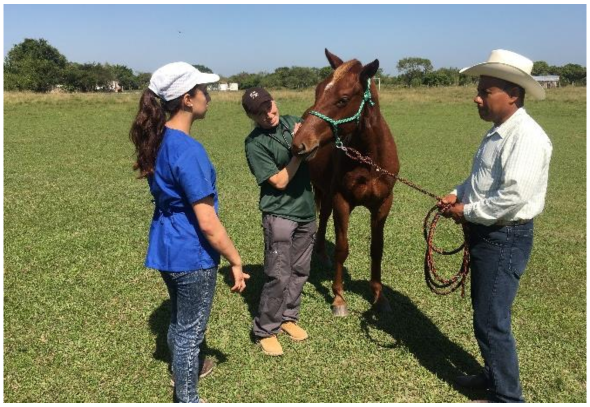

Day 3. Alex (left) and MacKenzie (right) examining horses at San Sebastian.

Most of the equids at San Sebastián that came to the clinic were horses with a smaller number of donkeys. The animals were generally in good body condition and most of the animals were used for transporting people and herding cattle. One of the most common problems we saw was external parasitism with ticks. Many animals also needed dental care and farrier work. The UNAM students were helpful with translation and explanation of local customs and traditions involving the animals. All in all, we evaluated about 100 animals in San Sebastián – under a bright blue sky which all Michiganders appreciated despite sweating quite a bit through the day.

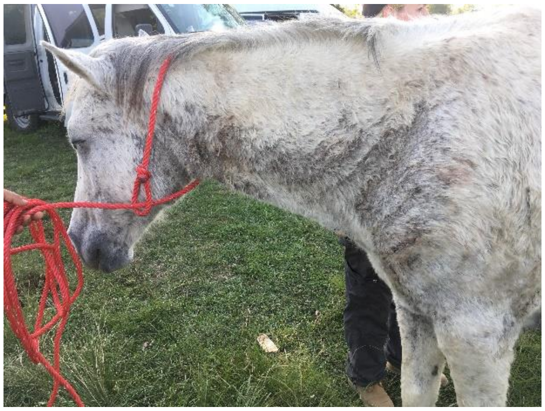 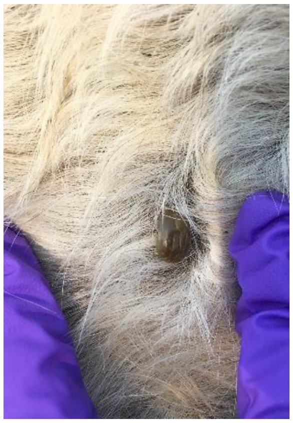 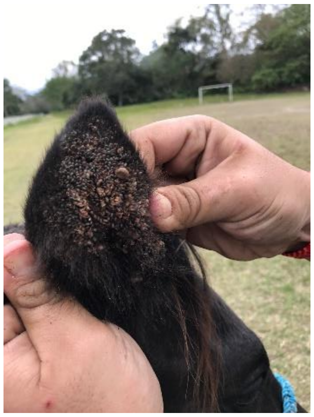

Day 3. Ticks are a big problem in some communities and in addition to causing dermatitis they can hide out in ears and transmit infectious diseases.

After we finished working as the sun was setting (about 6:30 pm), we (all 24 of us!) were invited to a local family's home for dinner. They prepared a meal with homemade tamales, a spicy chicken stew, and rice. Unfortunately, we couldn't stay for long because we also had a traditional barbacoa (barbecue) meal waiting for us back at Reuben's ranch. The barbacoa consisted of a lamb wrapped in banana leaves and slow roasted all day in a traditional brick oven over wood coals. A big pot with water and vegetables was paced on the coals under the lambs to catch all the juices to make a soup to accompany the lamb. It was a delicious meal and a true traditional experience

Day 3. Although we initially thought the lower limb dermatitis on this burro was due to standing in mud, we later learned that it was a burn injury sustained when the burro was in a field that was burned after harvest, a common practice in Mexican agriculture.

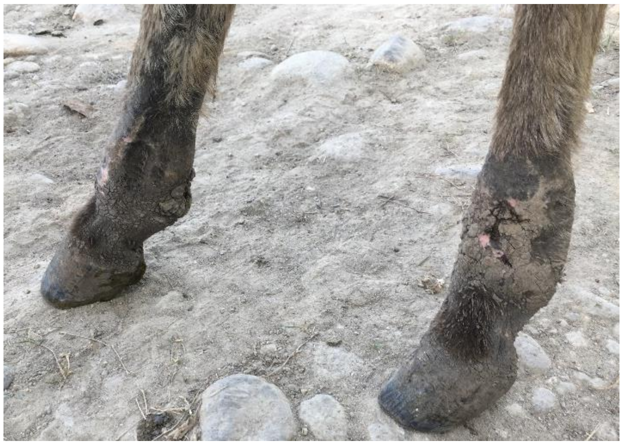

of rural Mexican culture. After dinner we ended the day by each person saying what they enjoyed most about the day and what we hoped to learn going forward during the project. It was after 1 am by the time we returned to the UNAM ranch and all were exhausted after our first day!

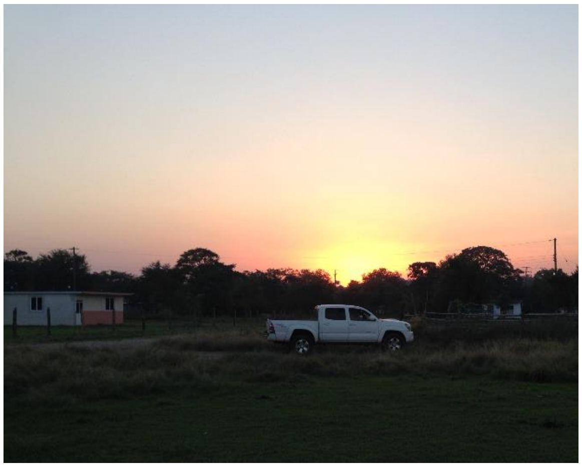 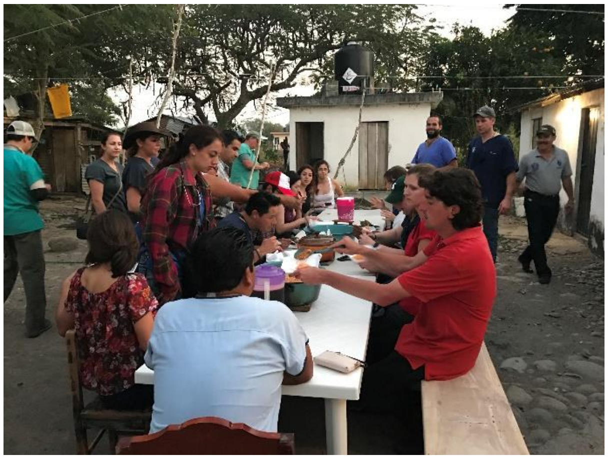

Day 3. Sunset at the end of the day (left) and a homemade Mexican snack of tamales and chicken at the home of a San Sebastian family.

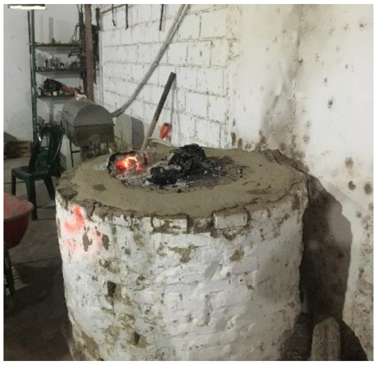 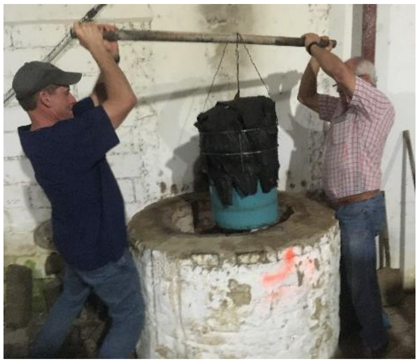 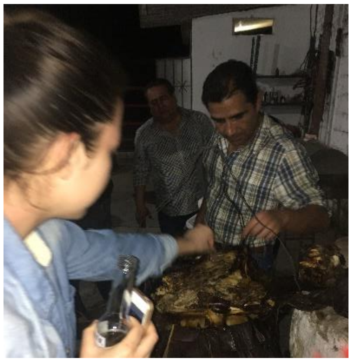

Day 3. A slow roasted lamb barbacoa was our traditional dinner feast after our initial day of community work.

Day 4 – Thursday 1-26-2017, by Lauren Kustasz

Today we woke up, left the ranch around 8 am, and ate breakfast at our usual breakfast spot. Then we left for a community named Laguna de Farfán. The drive was long, about 2.5 hours and towards the end quite bumpy on twisty-hilly roads. After driving for about 2 hours, we were making our way up a mountain when we reached a steep hill. It had rained a lot the night before so everything was wet and the road was a dirt road at this point so the ground was soft. The first van carrying the students from UNAM gunned it and tried to make it up the steep hill but had no luck as the wheels spun in the slick dirt. Dr. Arturo (one of the veterinarians at the Donkey Sanctuary), driving this van decided to try it without any passengers to lighten up the load. Even with no people and five of us trying to push the van, the attempt did not work and we were stuck. Since the sun was just beginning to shine on the road, we decided to wait a bit to let the dirt road dry and then try to make it up the hill. To fill in the lost time, Dr. Bunn and Dr. Arturo delivered an impromptu lesson about farrier work along the side of the road. It was a cool “classroom” lesson, with a beautiful backdrop of the green mountainous terrain and

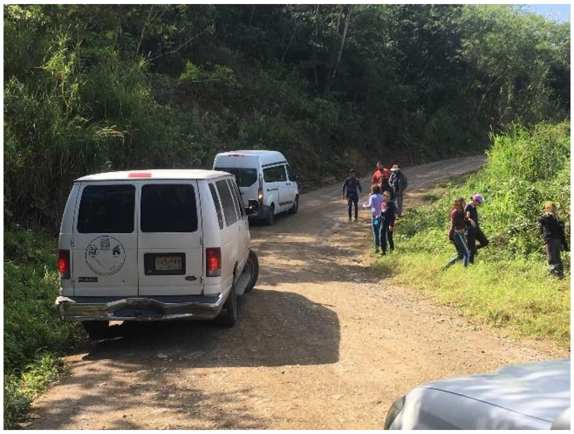

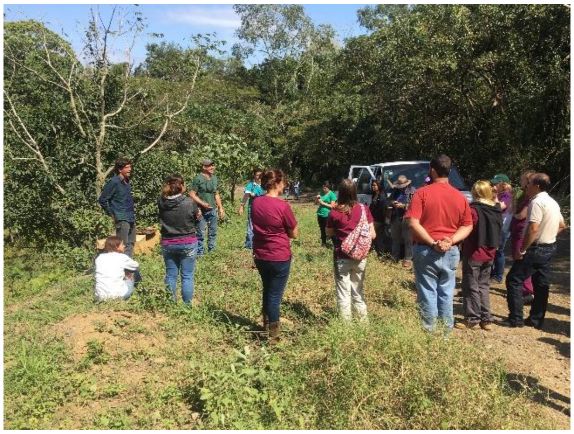

Day 4. A slippery road stopped our morning drive in the UNAM vans so we had an impromptu farrier lesson by Drs. Jeff and Arturo on the side of the road.

a few cows grazing below us. As Dr. Bunn and Dr. Arturo began showing us their instruments and talking about the importance of balancing feet, José (our wonderful interpreter), was standing nearby interpreting for us when he realized he had been standing on an anthill the entire time. It was probably one of the funniest moments of the trip thus far! José starting jumping around slapping at his legs and eventually had to run behind the van and completely undress to get all of the ants off his clothes. Luckily, our other awesome interpreter, Humberto was ready to jump in and continue the lesson for us as José ridded himself of the ants (we could not stop laughing). After this lesson the road had dried up enough for us to make another attempt to drive up the hill and this time we were victorious! In our van, we like to chant every time our driver (Luis) makes it past a rough spot in the road, so when we made it up this steep hill, our van was filled with our voices chanting LUIS! LUIS! LUIS!

When we finally arrived at the community (a couple of hours late), there were many horses, donkeys, and mules waiting for us. These equids did all types of work, mostly carrying loads and people, and were used in hilly terrain. Since this was our second day in the field, we had a better idea of how to organize things so we split up into groups of four to do physical exams, farrier work, dental work, and castrations. At the end of the day, we saw about 120 equids and did a handful of dentals, a dozen or so feet, and three castrations. The community members were very gracious for our services, although some were frustrated with the amount of time they had spent waiting (this was understandable considering that some of them traveled many hours to get to us). The students working with Dr. Bunn doing farrier work said that compared to the community the day before, these equids had much harder feet (this is a very good thing for working equids because it leads to less feet problems and less lameness). Most of the equids in this community were VERY well behaved and did not mind having us work on them. There were, of course, a couple that gave us troubles, including two nervous mules and a rambunctious foal, among others. Throughout the day, we got to listen to a hilarious parrot yelling “Hola” and singing La Cucaracha from a home nearby.

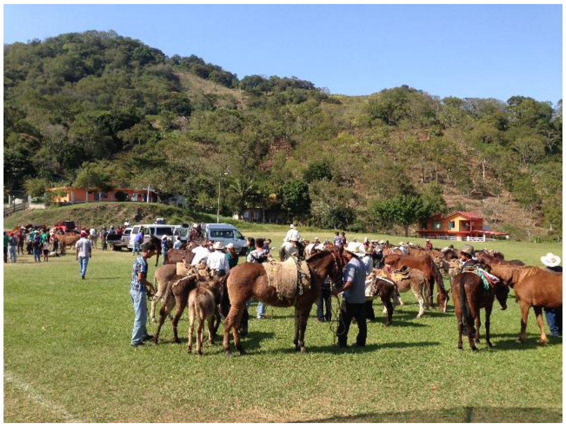
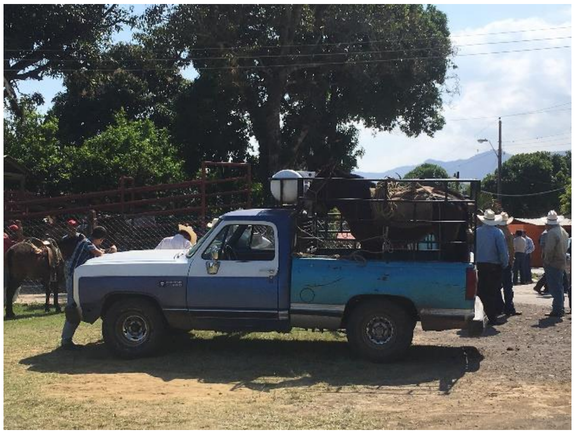
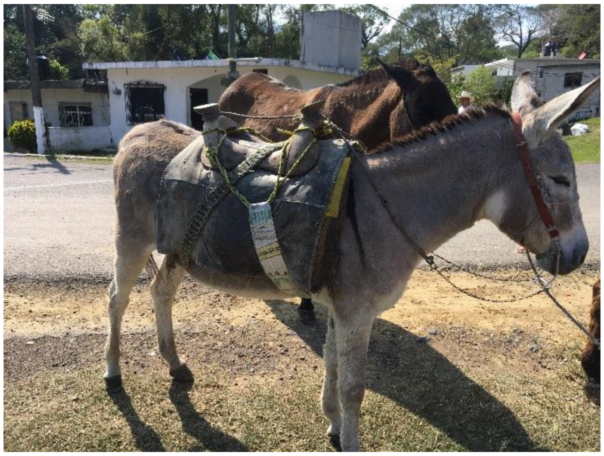
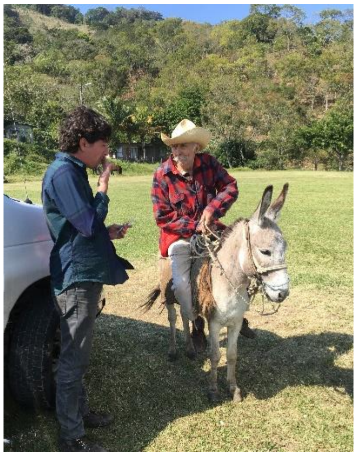

Day 4. We arrived at the community of La Laguna to a crowd of waiting equids (upper left). Some came in the back of a truck (upper right) while others walked up to 4 hours to get to the community (and a couple would walk back 4 hours in the evening after being anesthetized and castrated). A burro was saddled with the local double horned saddle for carrying jugs of water or milk (lower left) and José talks with an older gentleman that used his burro for transportation (lower right).

As a part of this externship, each of us had to choose a topic to study while we were here. Since I am very interested in ophthalmology, I decided to do my project on determining the types of eye problems that are common in this region of Mexico. I got to see my second ophtho case while at this community; it was a horse with a non-painful, but injured eye. Dr. Schott helped me do an examination of the eye and we found that its retina still functioned, however it did not have a reliable menace response (probably due to all of the corneal scarring). The owner told us that about a year ago the horse had been out on the field and when it came back its eye was injured. Since the eye was non-painful and the owner told us that it never seemed to bother the horse, we determined to leave it alone. If the eye had been painful, or was painful at certain times (like with anterior uveitis) we would have likely performed an enucleation.

The day ended late (around 8 pm) because we got such a late start and the General (one of the Donkey Sanctuary doctors) started the day with castrations and ended by

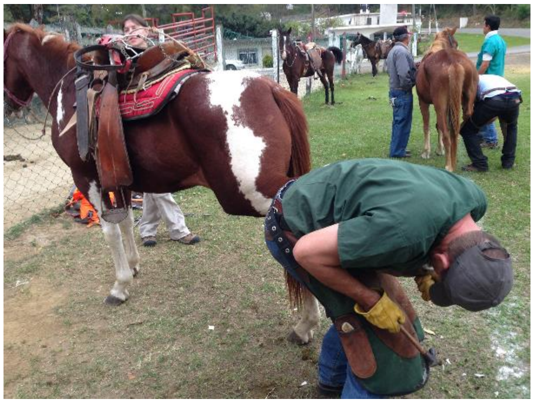
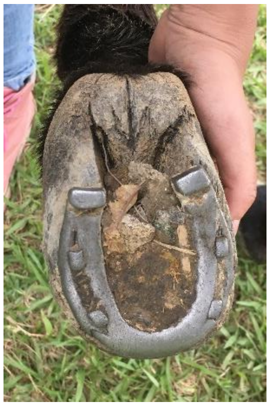
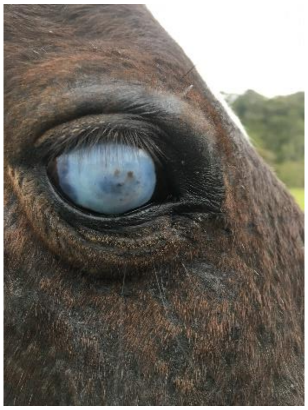
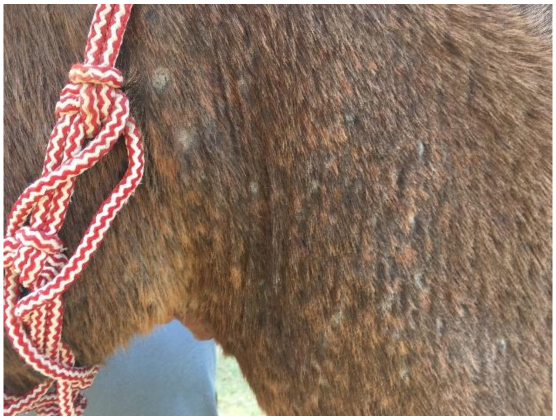
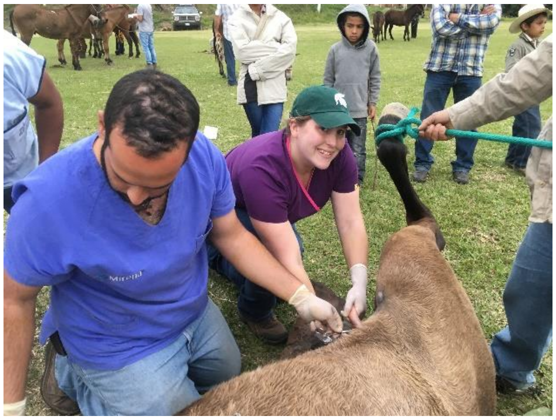

Day 4. Dr. Bunn trimming a horse (upper left) and a typical shoe (a bit small with large calks) for working in the hilly terrain (upper middle). An old eye injury resulted in a non-painful but opaque cornea (upper right). Fly bite dermatitis along the neck of a horses (lower left) and Bernie and Olivia performing a castration under the General's guidance.

doing dentals in the dark. While we were waiting to go eat dinner, a couple of us MSU students sat in the van with Roberto (a veterinarian with the Donkey Sanctuary), Humberto and Bernie (one of the students from UNAM). We traded some English and Spanish tongue-twisters and played charades. It was a lot of fun, and was a really good chance for us to bond with each other.

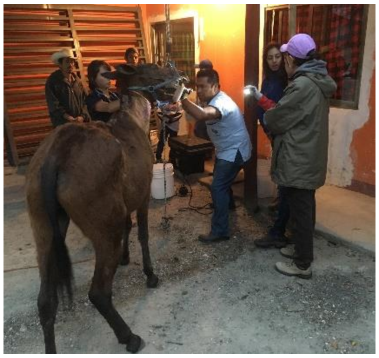
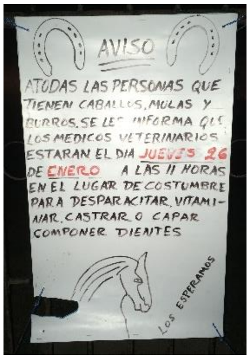

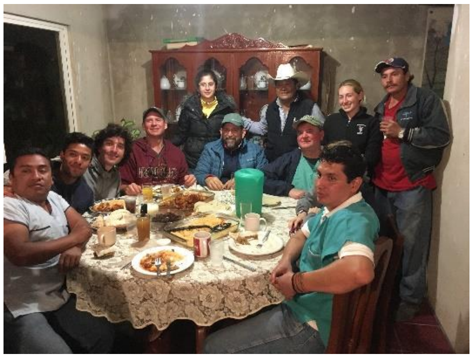

Day 4. Dentistry continued into the night (left) followed by a great dinner in a community member's house (right). The sign announcing our visit to the community (middle) had been on the community's member's front gate for the week prior to our visit.

We ended the day by eating dinner at a community member's home and watching a fútbol game in an adjacent covered asphalt court. The dinner was rather unique, because there were about 25 of us there to eat and they only had one dinner table with 8 chairs. So, we just took turns at the dinner table and conversed while others were eating. It is really cool to get to spend time in peoples´ homes and experience their culture. Everyone here is so nice and they make you feel like a part of their family. It is really neat and it warms your heart. We got back to the ranch around 1 am and we all went right to sleep after a long day of work and traveling.

Day 5 – Friday 1-27-2017, by Emily Thomas

Friday was another great day! It was our third day going into communities and treating horses and donkeys (and a puppy!). After a stop at our breakfast buffet restaurant we headed back up into the Veracruz hills - the community we visited was Colipa. It was a beautiful little town in a very hilly area. Here we mostly saw horses that were used for riding and some ranch work, and also a fair number of burros used for transport of goods and people. Interestingly, many of the horses were also used for an annual all day ride (and party) between several of the communities – over 3,000 horses participated in this ride each year.

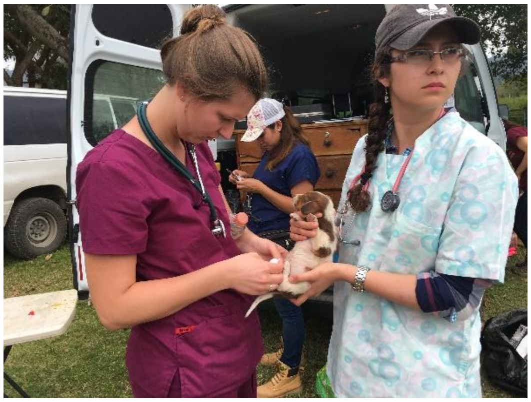

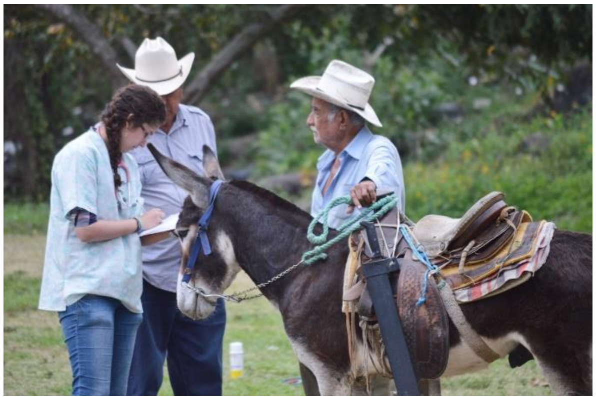

Day 5. Lauren (MSU student) and Flora (UNAM student) treat a small puppy that a young boy brought (left). Flora (UNAM) taking a history from a burro owner (right).

Our team broke into groups to begin doing physical exams, castrations, farrier work, dentistry, and educating members of the community on proper harness fit. Today was the largest number of castrations, keeping the General and Dr. Jeff busy and providing many of us an opportunity to assist with our first castration. Our groups were working fairly well by now and in addition to castrations, a number of us got quite involved in farrier work. Friday was a really meaningful day for me personally as I did my first castration. I also learned first-hand about field anesthesia as I went down with my horse - I broke my glasses but was otherwise unhurt. I also felt my first pregnancy on rectal palpation and I noticed a lot of improvement in how I approached nervous horses and donkeys. I am learning a lot about handling and behavior and I am much more

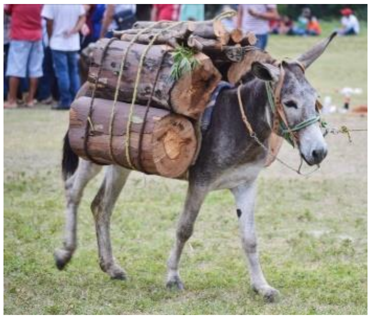 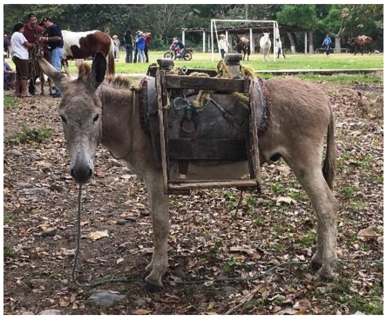 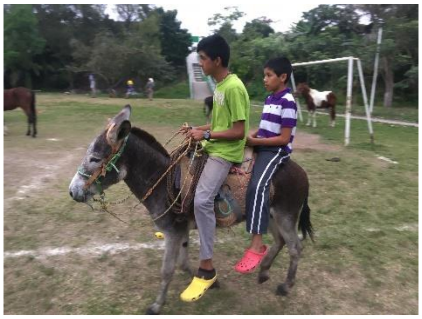

Day 5. Burros are amazing – they seem to be able to carry almost anything!

comfortable vaccinating horses that are not really happy to be handled, let alone stuck with needles.

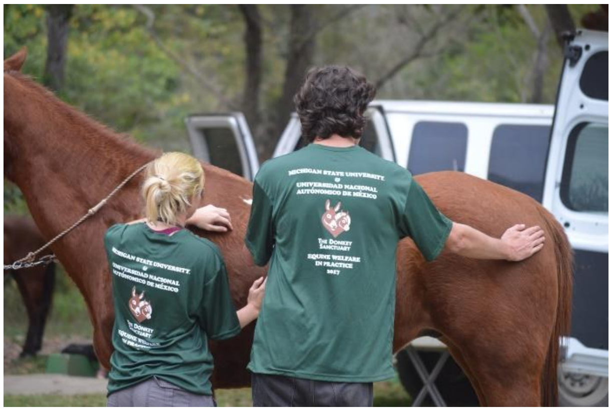 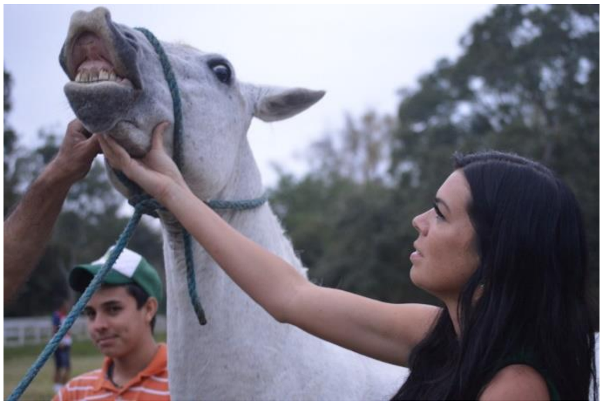

Day 5. Mackenzie (MSU student) and José (Donkey Sanctuary staff) performing a physical exam and showing off their MSU-UNAM project shirts (left). Sam (MSU student) with a horse after giving an oral dewormer.

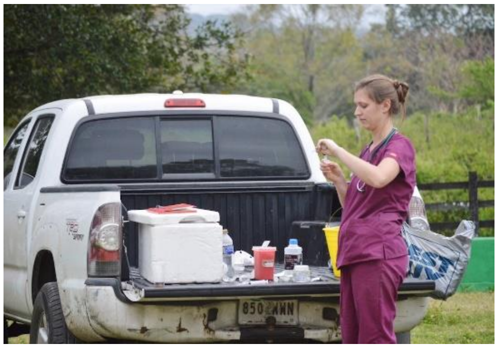 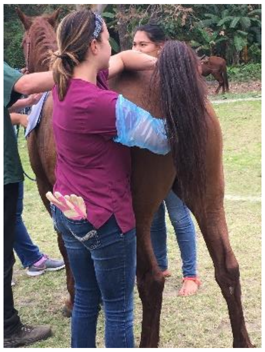 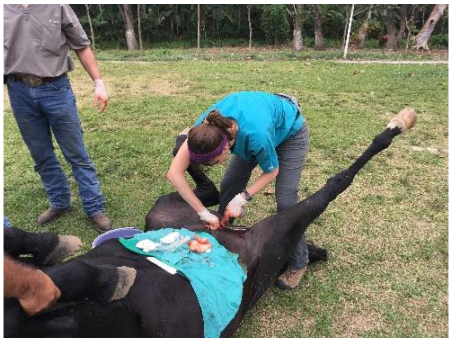

Day 5 – Lauren drawing up a vaccine (left); Alex palpating a mare (middle); and Emily castrating her first horse (right)!

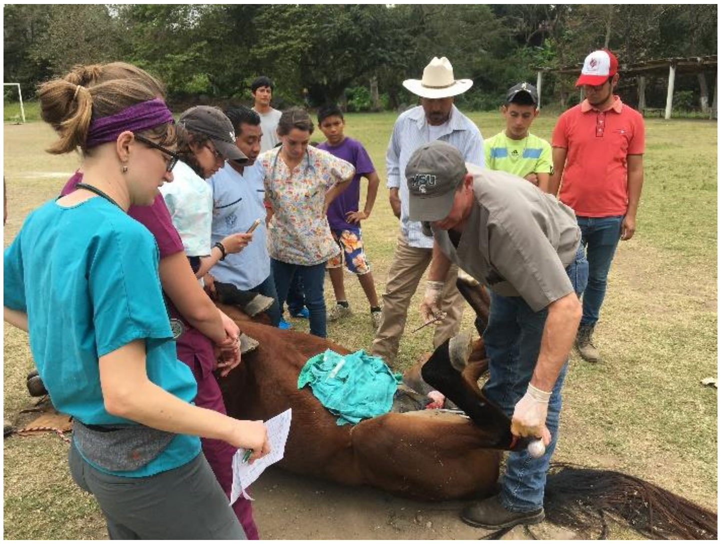
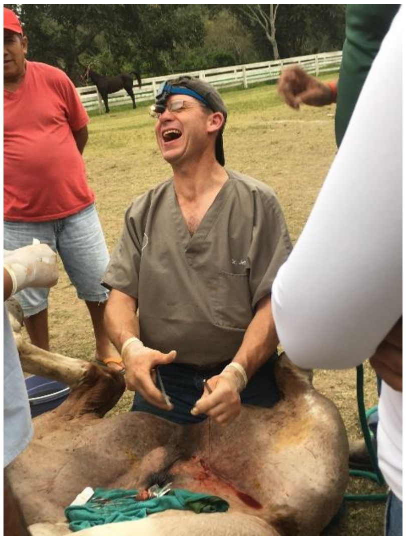

Day 5. Today was the day of many castrations, keeping both the General and Dr. Jeff busy for most of the day – Dr. Jeff was also happy as he was able to successfully complete a cryptorchid castration in the field.

As the day wore on, a group of community children started playing soccer – we were working on their soccer field – but we were too tired to join in. At the end of the day, we were once again fed by the community – they set up a long table in one of their homes and served us a great chicken soup with beans and tortillas. After dinner it was time for a group photo in the local town square before heading home for our last night at the Veracruz ranch.

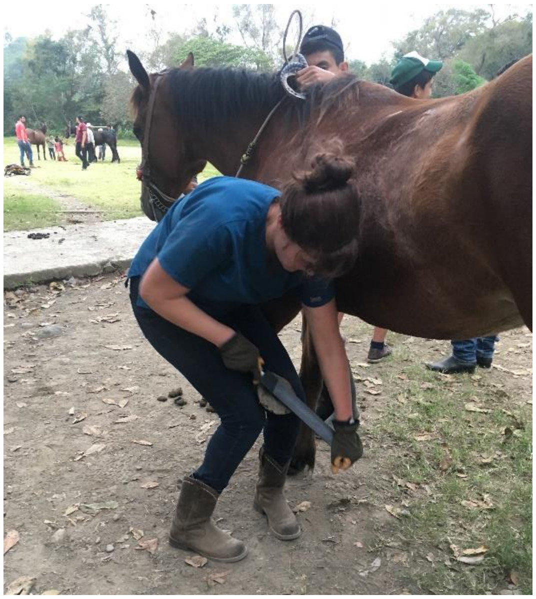
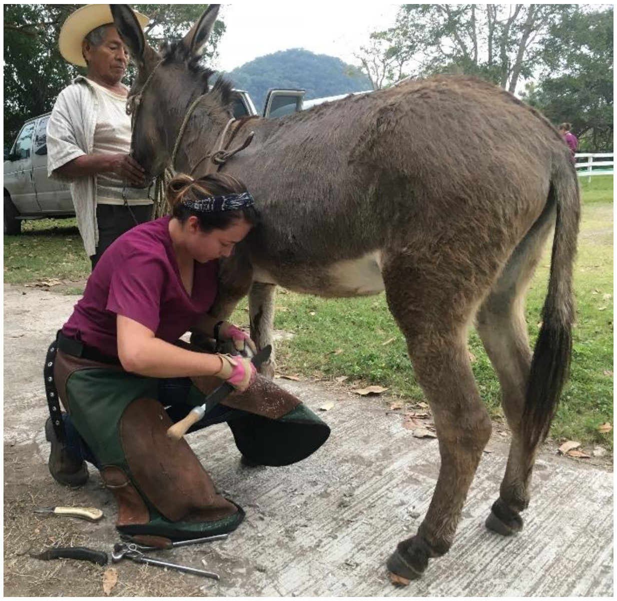

Day 5. Andrea (left) and Alex (right) improving their farrier skills.

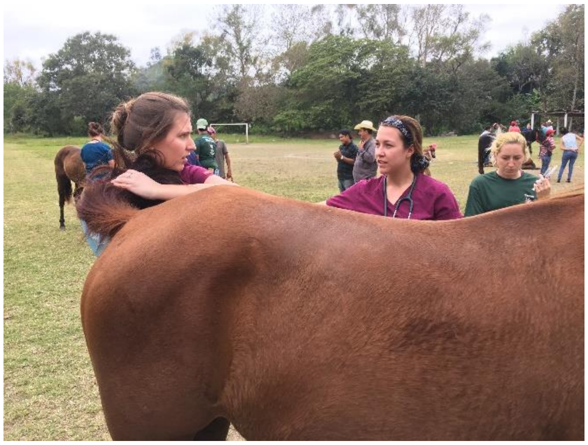 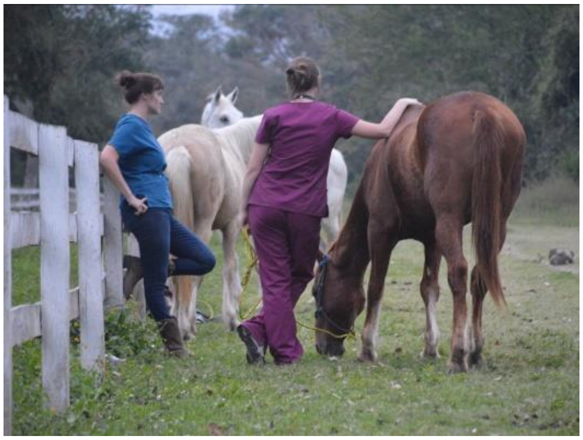

Day 5. Alex talking Lauren through palpation of her first pregnant mare (left) and Andrea and Lauren taking a short break at the end of the day (right).

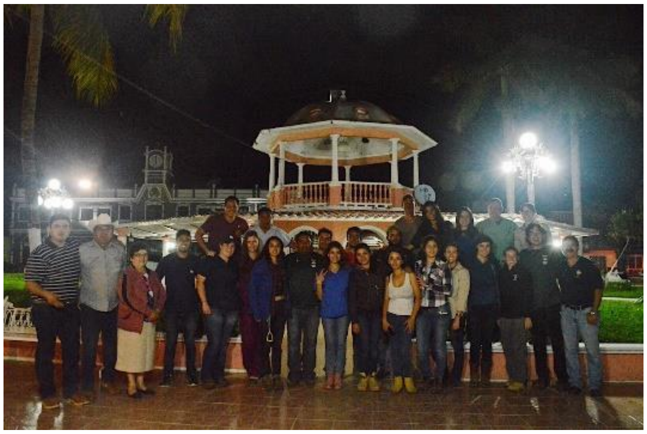 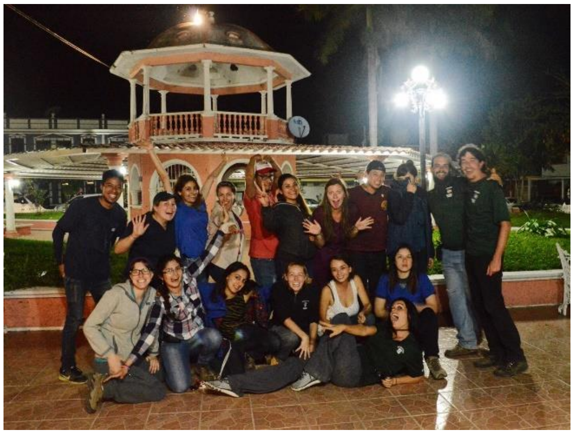

Day 5. A group photo in the town square after a delicious meal with a host family (at far left) from the community and MSU and UNAM students having some fun!

Days 6-7. Weekend 1-28&29-2017, by Alex Pollock

After a fulfilling first week of work, we were all ready for some down time. After a quick stop at the cheese shop at the Veracruz UNAM ranch, we bid farewell to the ranch and headed to breakfast as a group, MSU and UNAM students and the Donkey Sanctuary staff. After breakfast our Veracruz UNAM ranch hosts, Manuel and Ivette (UNAM ranch faculty) took us to Cascada El Encanto in Atzalan, a waterfall upstream from the river that flows through the UNAM ranch. Here we piled into 10 person rafts and paddled a short way upstream to a beautiful waterfall! Some of us were adventurous enough to jump into the water and swim right up to the falls. The water was refreshing and much warmer than whitewater rafting rivers in the US. After our visit to the waterfall, we drove a short way and stopped at a beautiful look out spot where we warmed up with coffee and Roberto, a recent UNAM graduate that is now a veterinarian for the Donkey

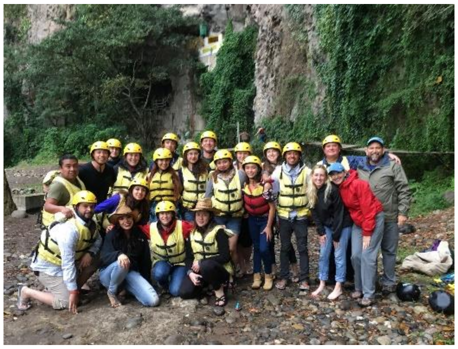 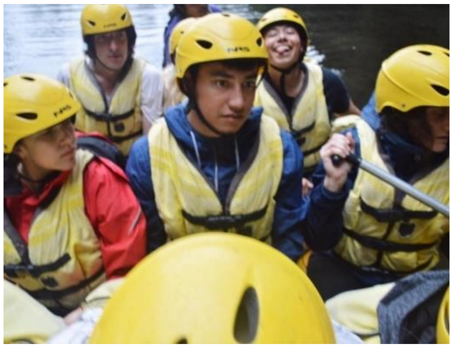 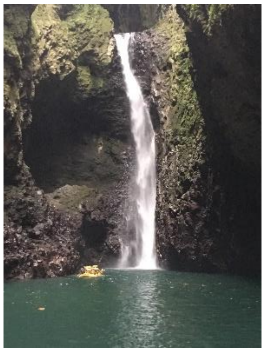

Day 6. Cascada El Encanto, a waterfall in Atzalan, was a late morning stop before our drive to Veracruz port.

Sanctuary, showed us the basics of salsa dancing. After that we started the 4 hour drive to the Port of Veracruz where we were looking forward to a day of relaxing on the beach. We arrived at our hotel around 9 pm, washed up quickly and went for a late dinner at Bennigan's.

Sunday was our designated "free day" and most of us took the opportunity to sleep in a bit longer. Unfortunately, the wind was blowing quite hard and the waves were high so a beach day was out of the question. As an alternative, some of us went to the Veracruz Aquarium, regarded to be the best in Latin America (it was very good with a shark tube and a dolphin show). In the afternoon we divided into two groups: one group explored the Port of Veracruz and did some shopping and the other group travelled to an ancient fort called San Juan de Ulúa. Dr. Schott even managed to catch the afternoon win by the MSU men's basketball team over the Wolverines – and we taught our Mexican colleagues the importance of “Go Green”! Around 6 pm we reassembled and went to a restaurant for a delicious seafood dinner in Veracruz city. We ate a range of dishes from sea snails to octopus to whole grilled fish, all caught in the Gulf of Mexico. After dinner the students worked our dinner as we "salsa'd" the night away while the remainder of the group enjoyed a tequila! Our dance lessons from Roberto definitely came in handy!

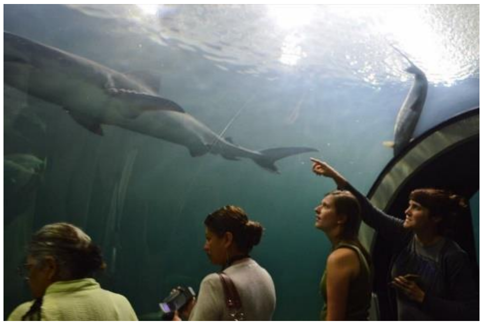 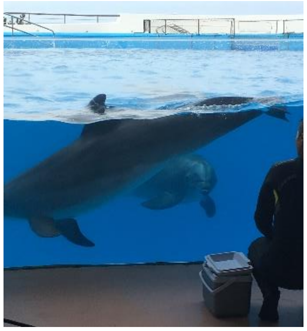 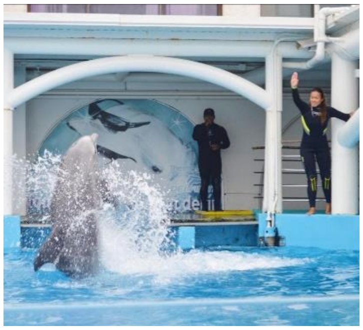

Day 7. A visit to the Veracruz aquarium.

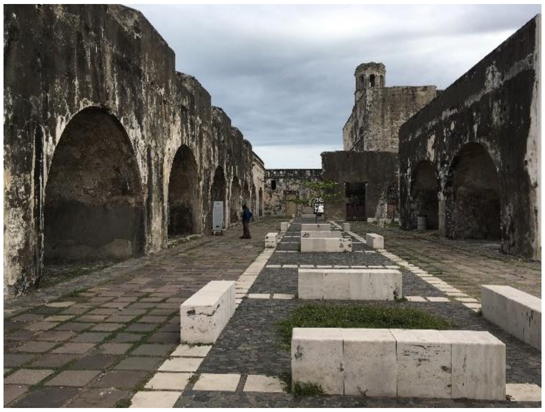 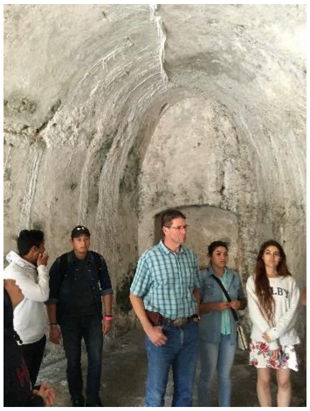 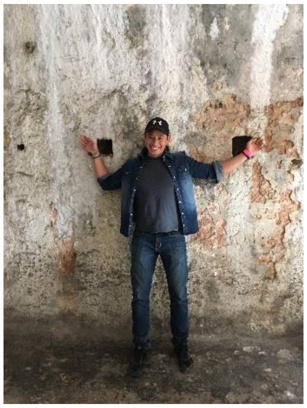

Day 7. San Juan de Ulúa fort and prison. In addition to a fortification for protection of the port, this facility was used to store goods awaiting transport by sea. The Moorish architecture (arches, left picture) shows the Spanish heritage and the fort was later used as a prison for political activists and criminals. Large rooms (middle) held 40-60 prisoners with more recalcitrant prisoners restrained in a standing position as water dripped on their foreheads (demonstrated by Roberto, right). The worst cells were termed Purgatory and Hell that were largely dark with small, long channels that only let in a trace of light.

Overall, the weekend was truly unforgettable. Communication between MSU and UNAM professors and students and Donkey Sanctuary staff was continuously flowing as new friendships were forged that will last long into our future careers as veterinarians.

Equine Welfare in Practice Blog – Week 2

Day 8. Monday 1-30-2017, by Hal Schott

Today we left the comfort of our Veracruz city hotel and drove the vans several hours to a very remote community called Santa María Tetetla. We pulled into a rough parking area adjacent to a bridge crossing a small river and were greeted by groups of braying burros – the day could be nicknamed "utter chaos". Fortunately, as we were unloading our vans and drawing up vaccines, José and Humberto started talking with all of the equid owners and developed lists and number of animals to create some semblance of order for the day. The burros in this community were largely used for hauling various types of produce to markets several hours away. Thinking about the very bumpy and slow progress we made over the last hour of the drive to this community, it was obvious why burros were ideally suited for this community.

We broke up into our teams and got to work – by the end of the day we had examined 208 burros, 44 horses, and 33 mules. Although most of the male burros were not castrated, there was neither time nor a good location for castrations at this work site. In addition to vaccinating and deworming most of the equids, we also performed four dentals, palpated four mares for pregnancy, and trimmed the feet of 25 animals. We again saw a number of ticks on the ears and around the anus of several animals and a new finding – vampire bat bites on the upper leg and lower neck of several animals.

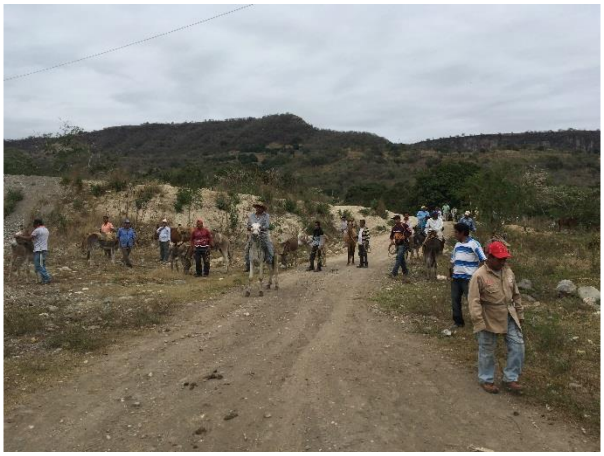

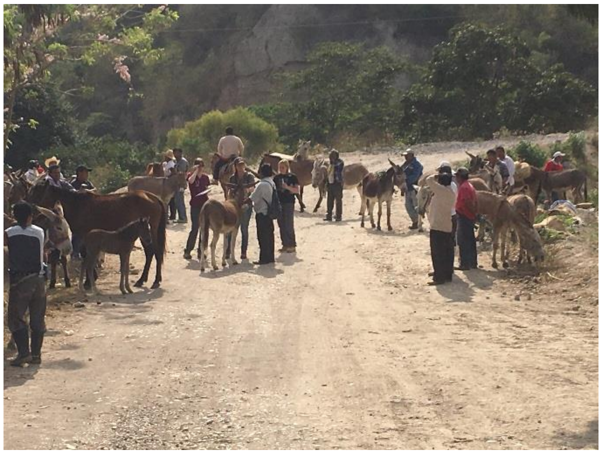

Day 8. Arriving in Santa Maria Tetetla we were greeted by many burros in all directions.

Overall, the equids in this community were not as well behaved as in the other communities where we had worked. It had been about 5 years since the Donkey Sanctuary staff had last visited this community and we were warned that the owners did not have the knowledge about how to more effectively work with their animals. Thus, we saw more ear twisting and sticks being used to discipline the animals, rather than the better interactions we had seen in other communities. This difference made it clear to us that education and repeat visits by the Donkey Sanctuary truly improve equid welfare in more ways than simple veterinary care.

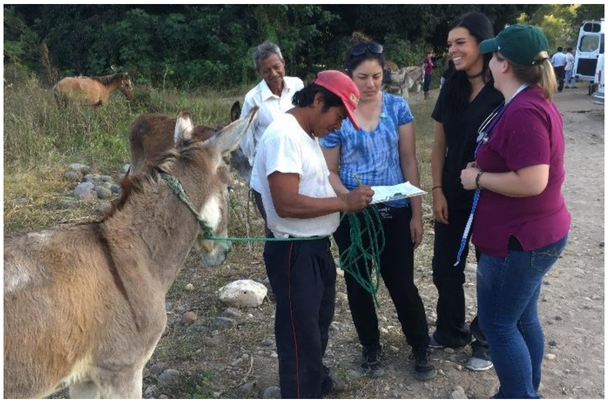
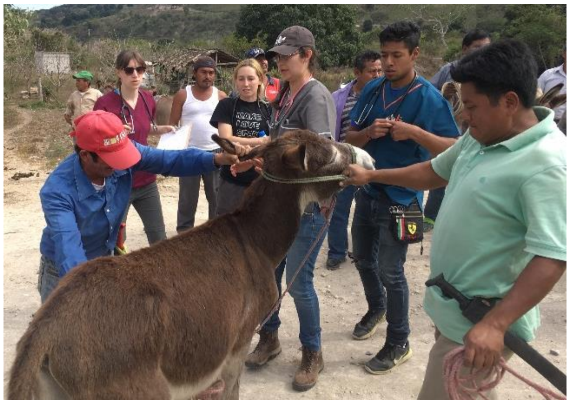

Day 8. MSU students Jackie, Sam, and Olivia talking with the owner of a calm burro (left) while Emily and Mackenzie look on warily while UNAM students Flora and Carlos prepare to vaccinate and deworm a less cooperative burro.

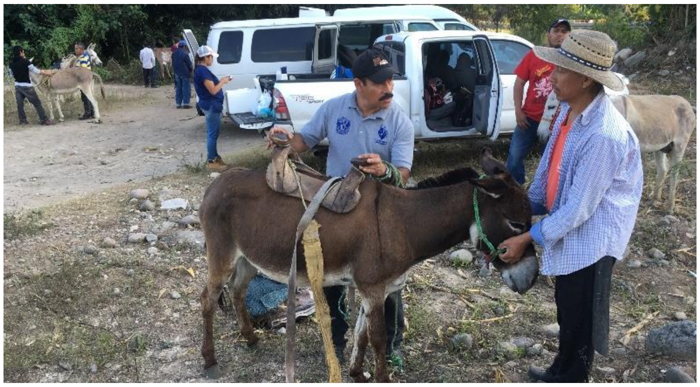
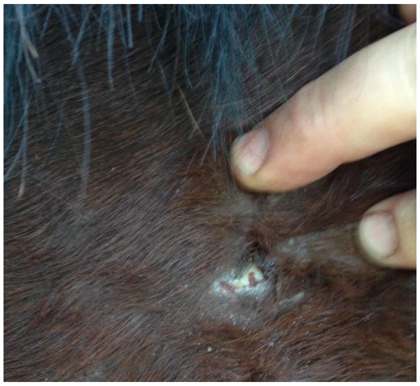

Day 8. Showing owners how to properly fit saddles is important for the prevention of wounds. Luis from the Donkey Sanctuary (left) is wonderful at helping people with the proper materials and balance to improve the working conditions of donkeys and horses. The wound on the neck of the horse (right) is a vampire bat bite wound.

Alex also examined a mare that was laying down and acting colicky – it was reported to have aborted a fetus the day before. The mare was dehydrated and Alex passed a stomach tube and we gave it a dose of Banamine and about a gallon of water from the local river, using a large pop bottle as a funnel. Rectal palpation revealed a lot of dry manure in the rectum but a vaginal exam was normal and within a few minutes the mare passed more manure and appeared to be on the road to recovery. We also saw a horse that was quite lethargic and in poor body condition. The oral membranes were quite pale and the mare had a systolic heart murmur, likely from anemia. Piroplasmosis was the likely diagnosis but we did not have any medications for treatment of this problem.

Fortunately, we all survived the day and both the MSU and UNAM students gained a lot of experience working with more difficult equids. The faculty and Donkey Sanctuary staff could see the students' confidence blossom as the day progressed and all were

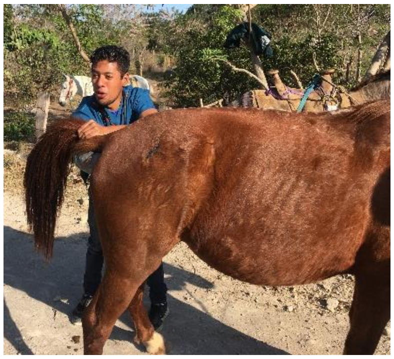
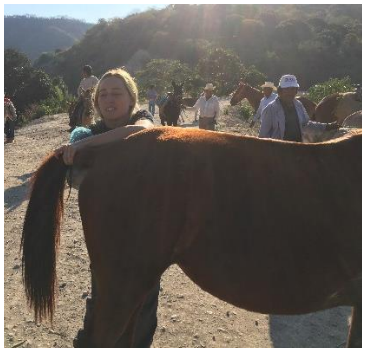
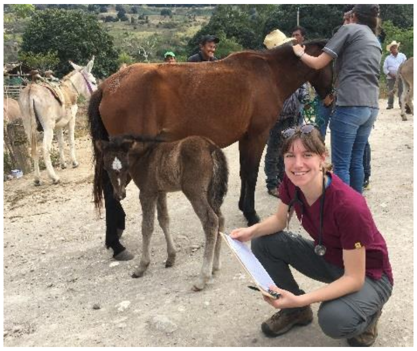

Day 8. Reproduction is an important part of the working equid “circle of life”. It happens in the old fashion way and there is no artificial insemination or embryo transfers. Thus, it is important for working equid owners to know whether or not females that may have been bred are pregnant, in order to prepare for a new foal. Carlos (left) and Mackenzie (middle) performed their first pregnancy exams at Santa Maria Tetetla while Emily (right) tries to bond with a 10 day old foal.

exhausted as a beautiful half moon started to rise in the sky. At the end of the day, we were invited to a community dinner where they shuffled together a number of tables in the street outside one of the community member’s houses. For desert we enjoyed bags of dry roasted peanuts, one of the products of the community. As we loaded up in the vans after dinner, it was also time to say goodbye to Dr. Bunn – he was driven back to Veracruz to catch a flight home early the next morning while we headed to our new hotel in the city of Xalapa.

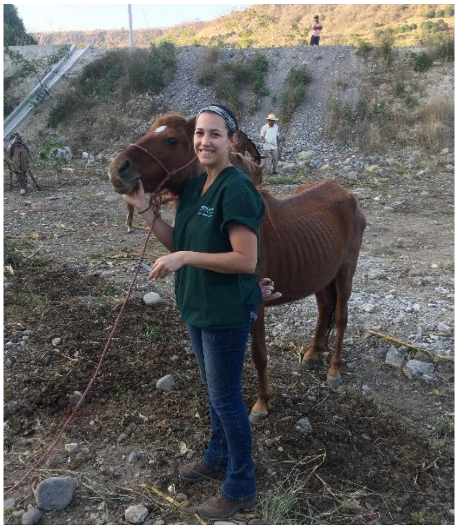
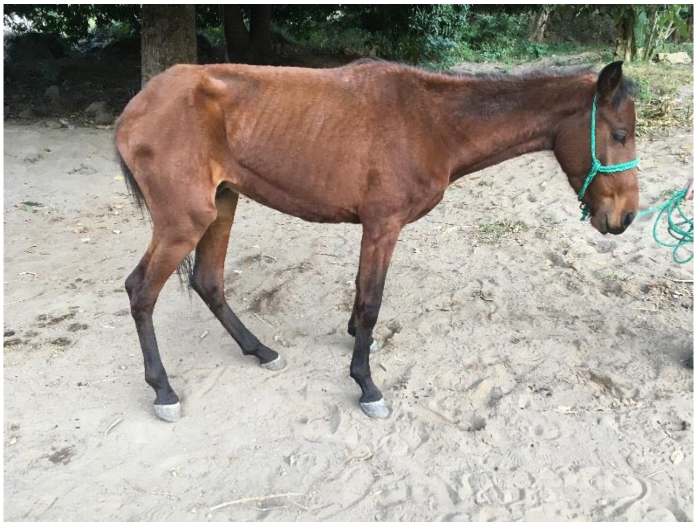

Day 8. Alex with a mare she treated for colic signs after the mare aborted a fetus the previous day (left) and another lethargic, thin horse with pale membranes suspected to have piroplasmosis (right).

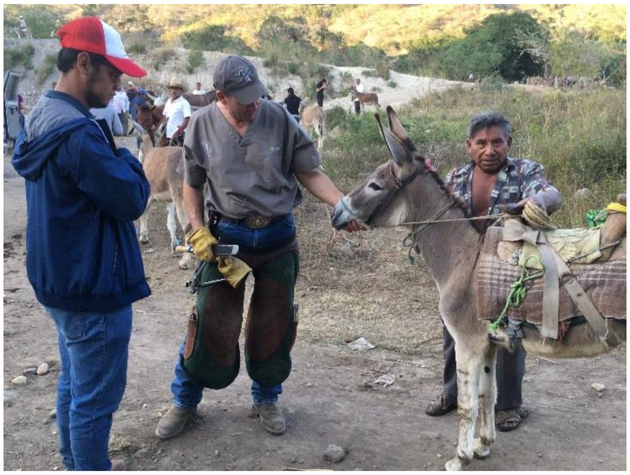

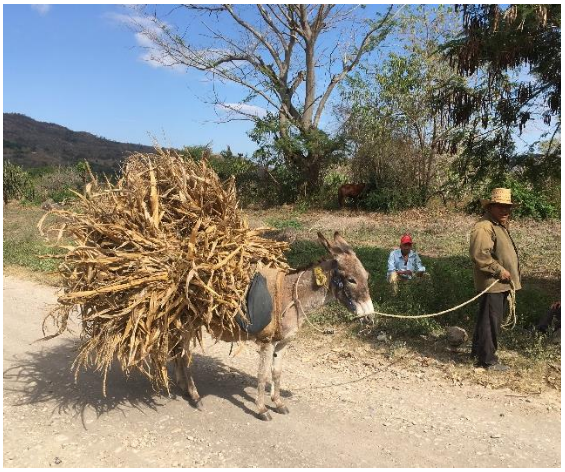

Day 8. This community was a long day of hard work. Dr. Jeff worked all day trimming feet but when we looked up and saw the burros working hard as well, it was no time to complain.

Day 9. Tuesday 1-31-2017, by Mackenzie Gallegos and Olivia Hornacek

We started the morning by walking through beautiful downtown Xalapa to get breakfast. There were many amazing buildings, including a cathedral, to look at along the way. The architecture and colors were gorgeous. At the restaurant, we watched a woman hand make tortillas and sopes. They were delicious just like the rest of the food. On the way back to the vans we could see the highest mountain peak in Mexico, the snow-capped volcano Pico de Orizaba (5,636 m or 18,491 feet high), in the distance.

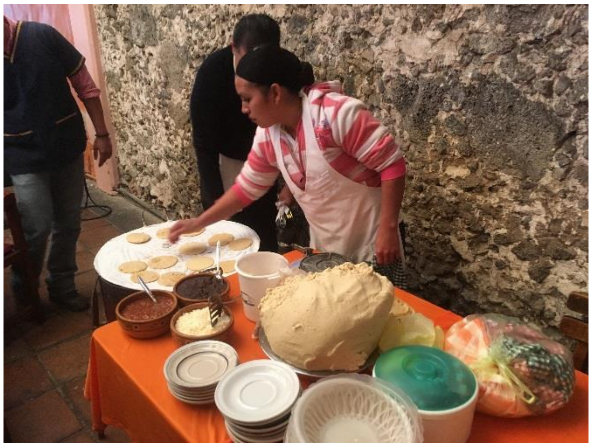

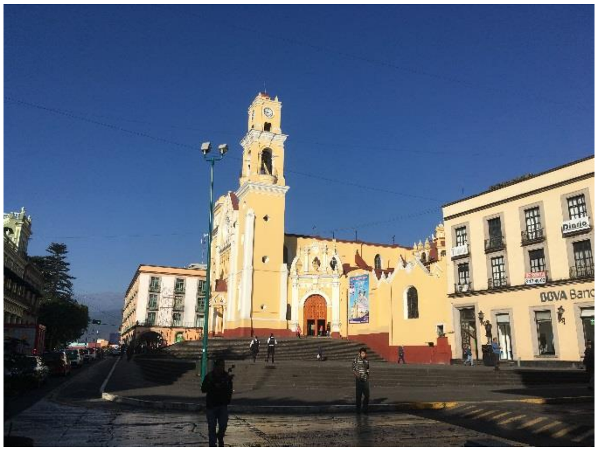

Day 9. No complaints about the food on this project – homemade tortillas and sopes for breakfast (left) and a walk next to the cathedral in Xalapa after breakfast (right).

We then crammed back into the vans for the hour and a half drive to the community of Miahuatlán. Members of this community primarily work in nearby cheese factories and many own small herds of cows that they milk by hand each day. The milk is then

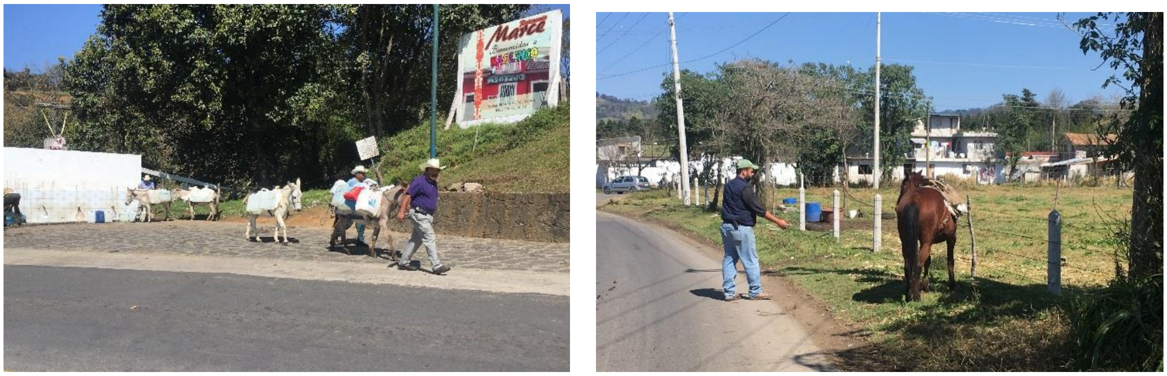

Day 9. On the way to Miahuatlán we saw donkeys hauling water from a community spring (left) and Bernie (one of the UNAM interns) rescued an escaped dairy transport vehicle – a horse waiting by the fence to haul milk to a local cheese factory (right).

transported by horse or donkey to the cheese factories where it is sold for 5.40 pesos/L (27 cents/L). On our drive in, we also saw many people loading their donkeys with large jugs filled with water from the community well. This water is collected daily and used in the household for drinking, cooking, and other purposes. Needless to say, the animals of this community are essential for both economic and livelihood purposes.

When we arrived at the work site, we were greeted by a small crowd of donkeys, horses, mules, and their owners. We split up into four teams to start our exams. José again made a “client list” and divided the names among the teams to make getting to everyone easier. Our teams were becoming well-organized and more efficient!

Today’s clinic was located in a small field next to the local Agriculture Association building. During this clinic, I (Mackenzie) worked at a physical exam station with MSU classmate Emily and UNAM students Flora and Carlos. We assessed the equids’ overall health, behavior, body condition, skin and feet and then we vaccinated and

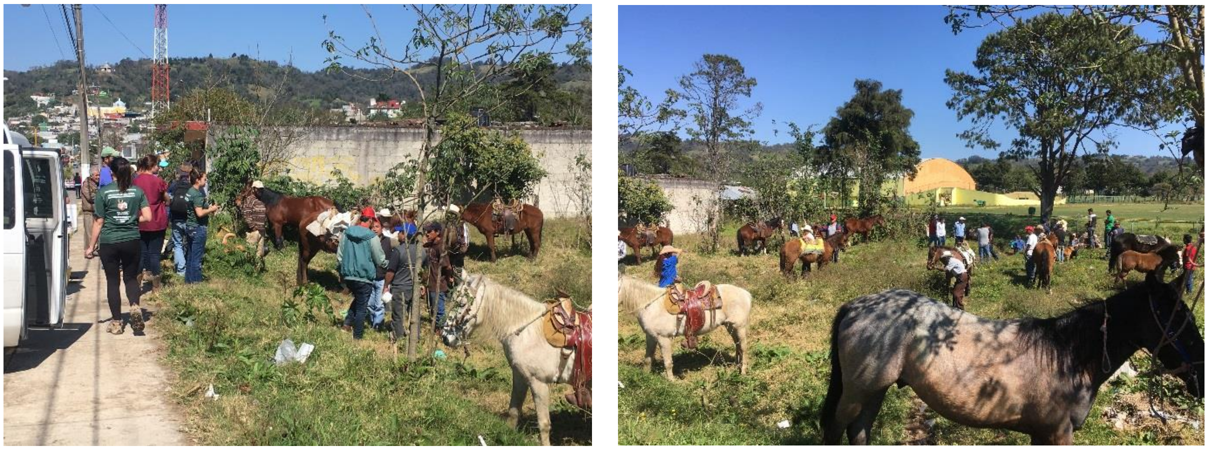

Day 9. Another crowd of mostly horses waiting for us at the Miahuatlán work site. There were no complaints about blue skies and warm weather on the last day of January!

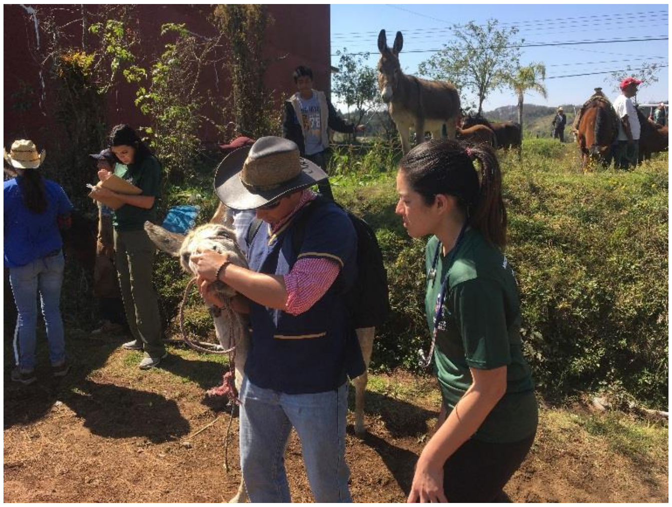 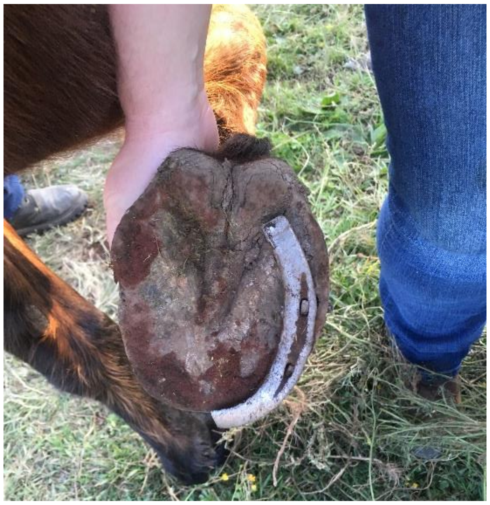

Day 9. Jackie and Dr. Arturo checking a burros mouth (left) and you never know what you might find when you lift up a foot (right).

dewormed the animals as primary preventative therapies. My UNAM colleagues Flora and Carlos were really impressive when dealing with more difficult equids. I learned a lot from just observing their methods of slowly approaching and quickly vaccinating those animals. My goal for the day was to become more comfortable aging horses by their teeth. I believe I accomplished this goal when I aged a horse and two of my classmates aged her the same!

The primary problems seen in this community were harness wounds from poorly fitted saddles and overloaded cargo. This makes sense because of the importance of the animals for economic purposes and the cargo being milk. We sent many horses to see Luis Aguilar who specializes in fitting saddles and harnesses.

Midway through the day, a group of cowboy hat clad men rode up on beautiful horses. It was a quite surreal experience watching these skilled riders navigate through the field of waiting donkeys and horses. It reminded me of Rio Bravo or another old western movie with the sheriff riding into town. These cowboys were part of the livestock and agriculture association and important people within the community. At the end of the day, I watched UNAM intern, Bernie, remove wolf teeth from a stallion. I also assisted “the General” in a field castration. It was the first time I had seen both procedures done. Some of the community men watched the castration and I joked with them “Quien es próximo?” Most of the stallions in this community were left intact unless they had specific behavior issues like jumping fences to get to mares. That’s just a fun fact because I don’t think I asked why this particular stallion was being castrated.

I (Olivia) participated in about five of the dentals today. I am really starting to enjoy them. Before this trip I had only held for the doctors which wasn’t always the most fun, especially when we did 15 Percheron dentals in a day. But being able to perform them on my own was very rewarding when you are making a difference in these working equids lives.

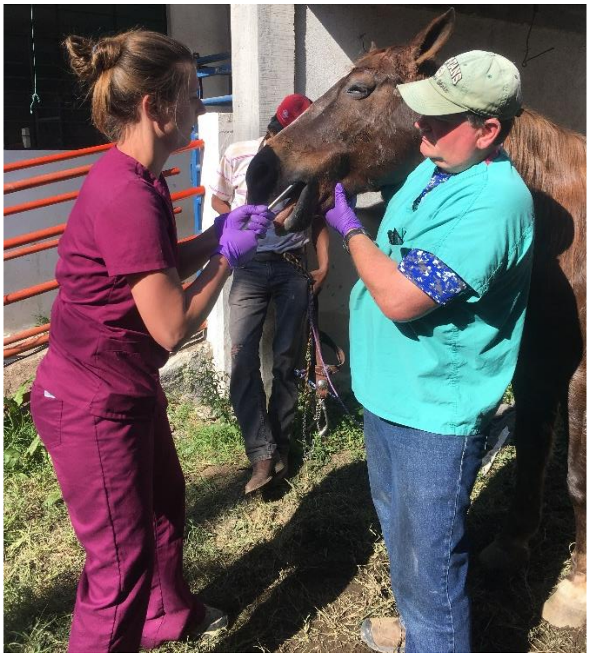

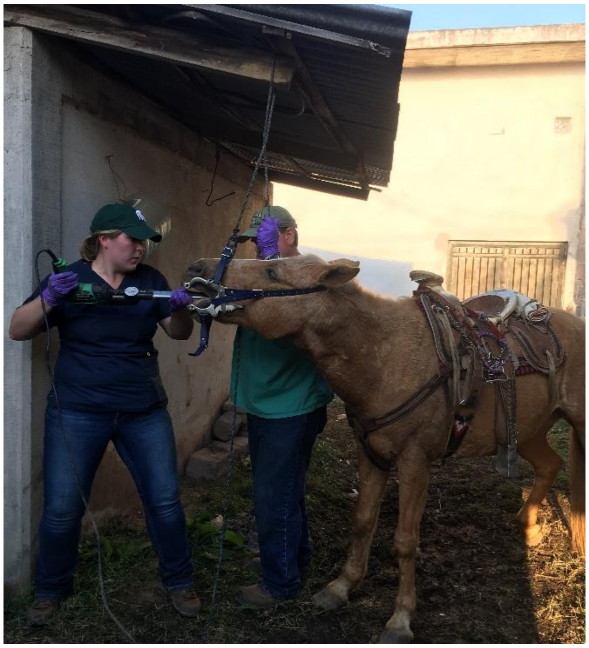

Day 9. Lauren (left) and Olivia (right) getting a lot of hands on experience with dentistry, using both hand floats and power tools!

In total we examined 85 equids, mostly horses with a few donkeys and mules. We performed 12 dentals, and 3 castrations. Unfortunately, part way through the day we ran out of our tetanus combo vaccine (500 doses had been donated to our project) so we could only deworm the remaining animals.

After our work day, we walked down the street to a home/store where a family had prepared us dinner. We ate Bistek a la Mexicana and it was the most delicious food I've ever eaten. I'm quite sure I (Mackenzie) could eat it every day of my life. We thanked the family and the cooks, two women smaller than me (I am 5 feet tall), for their hospitality and amazing food. We walked away as the sun was setting and I really felt humbled by the day. There are so many other places I could be, but today I spent the day in Miahuatlán, outside in the sun vaccinating stallions and burros while speaking (poor but passable) Spanish. We left shortly after dusk and headed back down the windy, bumpy road back to Xalapa. However, since the drive was comparably short and we were done a little earlier today than on previous days, we stopped for some shopping in Naolinco. This town is well known for leather goods and there were many shops with boots, shoes, sandals, belts, and bags. A couple of the girls went shoe crazy because the deals were too good to pass up. I really wanted a pair of the brightly colored sandals but unfortunately I could not find a pair in my size that I really liked. We then returned to our hotel for the night.

Day 10. Wednesday 2-1-2017, by Sam Gamble

Today we woke up after our last night in Xalapa and ate breakfast at the local restaurant. Afterwards many of the team stopped to buy Veracruz coffee (to take back

to the states) on the way to the vans. After about an hour we reached the city of Mazatepez and decided to have one of our final lectures about the importance of assessing the community. José (a sociologist with the Donkey Sanctuary) and Humberto (an anthropologist with the Donkey Sanctuary) talked about the importance of the community and the equid-human relationship in the work of the Donkey Sanctuary. They emphasized the importance of their work as sociologists and anthropologists in reaching many goals of the Donkey Sanctuary which not only involve veterinary work, but also work that involves the community that can allow for a sustainable solution for the communities and the welfare of their equids. As we were sitting and listening to lectures we watched as a boy walked his horse down the rocky terrain carrying a load of firewood. Mariano also showed us some examples of the importance of not jumping to conclusions about cases when wounds are present. He introduced us to a man who had his young old son handling their horse. The horse had evidence of previous saddle

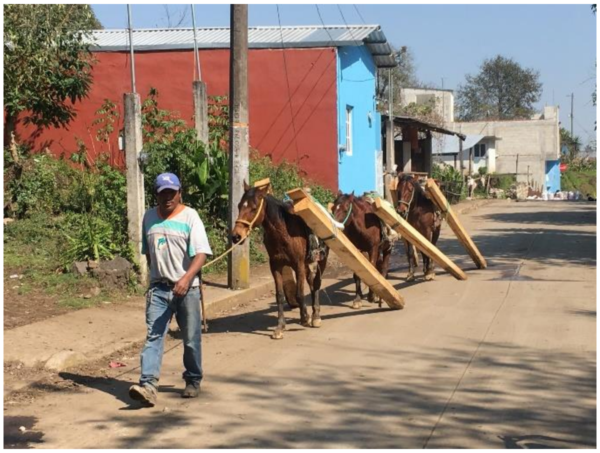

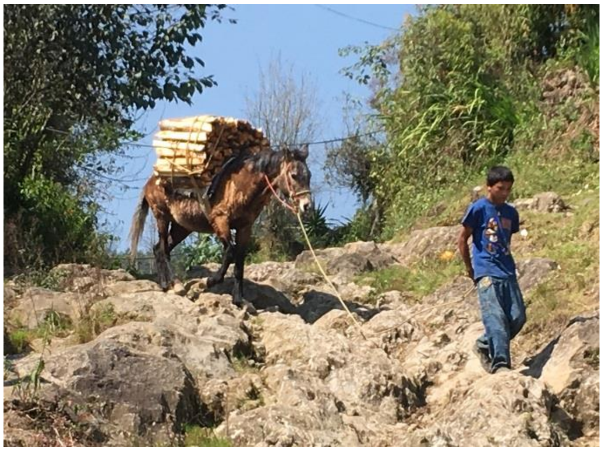

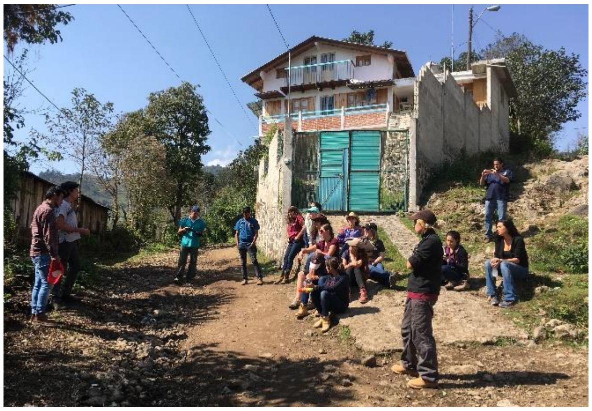

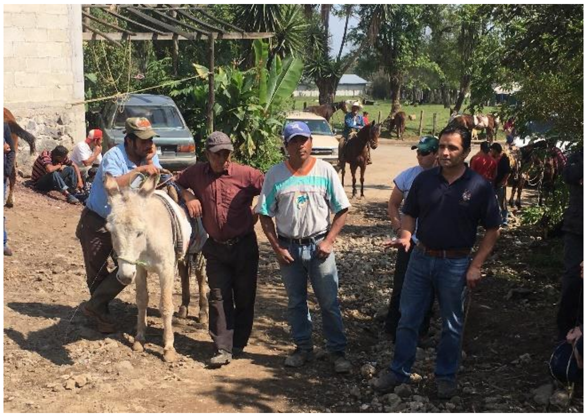

Day 10. Horses dragging heavy boards from the saw mill (upper left) and carrying loads of wood from the mountain (upper right) walked by as we having a discussion about community education from José and Humberto (lower left). Several community members joined in the lesson (lower right) to tell us about how they used their equids and also how an equid trader had come to their community and bought most of the community's burros a few years ago and now the community was in need of burros again!

wounds across his back that had healed. Mariano mentioned that many people may think that this man did not take good care of his horse, but in reality he had owned the horse for 10 years from the time he was a colt, and that the animal developed bursitis once from the hard labor and the General came out to treat it. Also, the man trusted his young son to handle the horse that was very well behaved, indicating the close bond between the horse and the family. Humberto also emphasized the importance of listening and learning about a community and its reliance on working equids. Although some traditional practices or treatments (e.g., placing a copper wire in the ear as a treatment for sarcoids) may not seem correct to us as veterinarians and students, it is important to understand how and why the treatment may have been pursued before passing judgement. Only once we understand a community can we effectively engage and offer further education to improve welfare of both working equids and the owners they serve. As part of their jobs with the Donkey Sanctuary, both José and Humberto may live in communities for periods of time to get to know the residents and observe their daily lives. This may include going to work with them, following them for some time to learn daily habits and traditions, learning how and why the different type of equids are being used, and getting to know important community leaders.

Following the discussion we began working – we saw many horses walking by carrying wood and corn stalks, but there was only one donkey present. Earlier in our talk it was explained that a few years ago a man had come to the community and offered 1,200 pesos ($60) to buy donkeys. Many people sold their donkeys not realizing that they were going to be sacrificed to harvest their skins (for extraction of skin oils to make “anti-aging” creams for the market in China). The only donkey present today was owned by a man who said he did not sell his burro because it was too important for his family. Because of this massive selling off of donkeys, horses are the only working equids in the community, but they require two to three times the amount of food each week that a donkey consumes. Today, donkeys cost 2,000-3,000 pesos ($100-150) and many

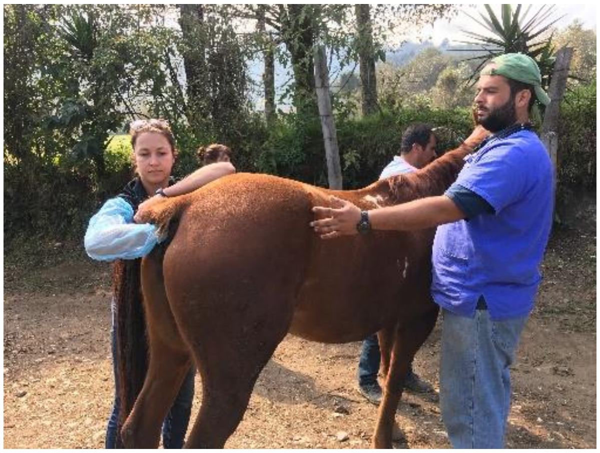

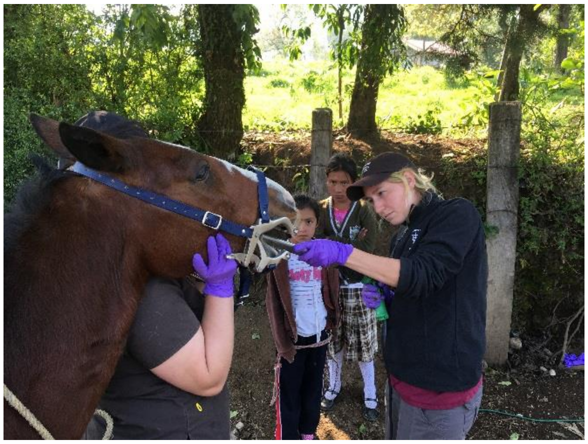

Day 10. Alex palpating a mare for pregnancy (left) and Mackenzie floating teeth of a horse (right). The two girls, ages 7 and 10, with Mackenzie used their horse every day for a 3 hour trip to the mountain to collect wood for their family.

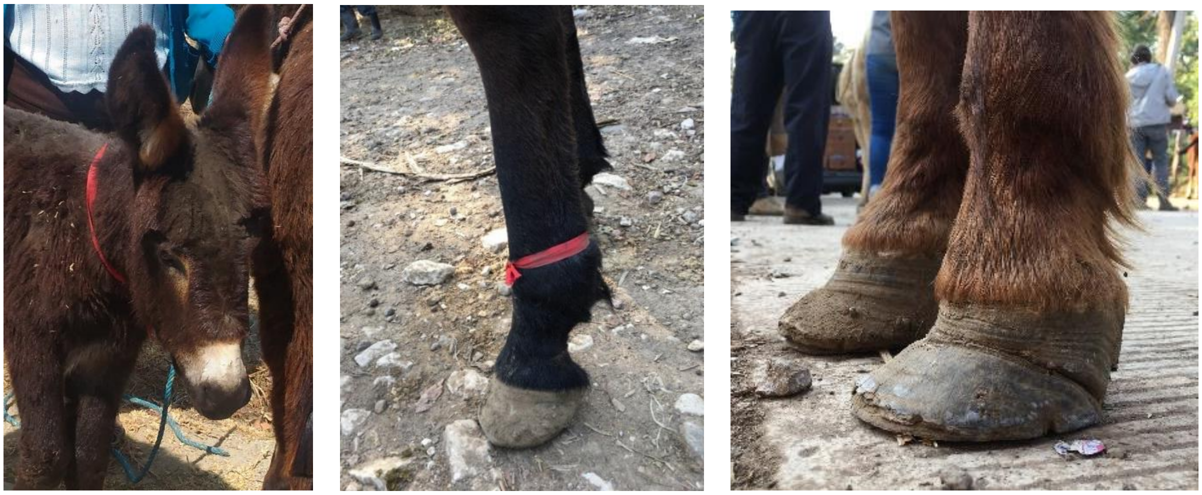

Day 10. Equid owners place red ribbons around their animals’ neck (left) or legs (middle) to ward off diseases and other problems. Maybe this practice could have helped the poor horse with chronic laminitis on the right.

people who sold their donkeys wish they still had them because they considered them better for carrying certain loads in the mountainous terrain surrounding this community.

Today we again saw a number of saddle wounds due to improperly fitted saddles and padding. Luis did a great job explaining to the people why the wounds had developed and provided helpful solutions. Luis mentioned to me that one of the reasons for the wounds is the mountainous terrain that can lead to the saddle sliding back and forth on the horse creating wounds. Additionally, today we saw a horse with numerous sarcoids that were removed, and we also saw a lot of horse shoes that were not the ideal size and position for the horses. The teams talked with the owners on ways to potentially improve the size and position of shoes in the future which could help in this type of terrain. People in the community were very kind and offered us Coca-Cola for

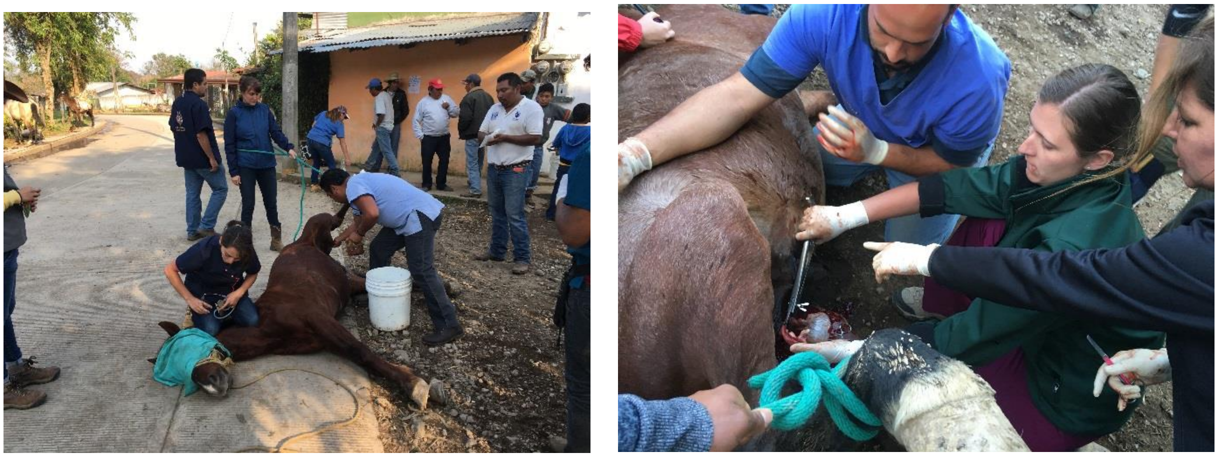

Day 10. Sometimes finding a good site for surgery was challenging – today we ended up anesthetizing horses for castration on the road (left); Dr. Elena is teaching Lauren and Bernie how to use the emasculators (right).

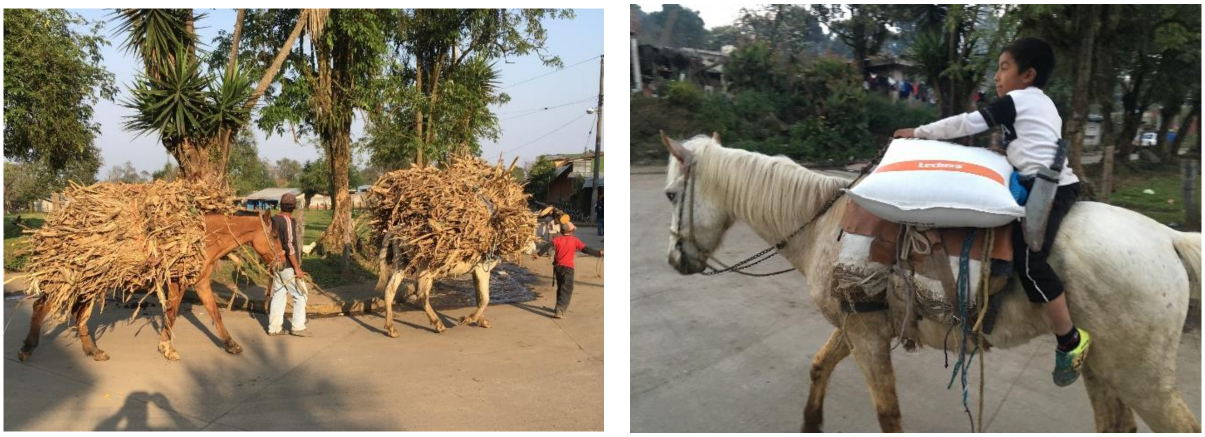

Day 10. Horses in this community were mostly used to carry loads – cornstalks (left) and a sack of food (right) with the little boy on the right having a machete just his size!

refreshment and asked us questions about our school and where we were lived. I spoke with a group of girls who said they were studying English and a man who had lived and worked in Kentucky for 4 years. We also performed about a half dozen dentistries and three castrations at the end of the day. One of the horses that presented for dentistry today was brought in by 7 and 10-year-old sisters. They were quite cute and told us that the two of them used their horse for about 3 hours every day of the week to collect wood from the surrounding mountains, while their two older brothers headed to Xalapa every day after school to work in construction for 6-7 hours. Thus, everyone had to work to support the family but they all seemed very content and reminded us of what is truly important in life.

Overall today was a success, we learned a lot about not only the importance of animal welfare but also about the important role the community plays in this. Understanding the people within the community, including learning, respecting, and understanding their daily lives, cultures, and traditions is a key component of this project. I have a great amount of respect for the Donkey Sanctuary for making the community a huge part of their program, and for spending a lot of time and effort to spend time within in the communities to learn from them to be able to offer a more sustainable solution for them.

At the end of the day we all piled into the vans and headed to a local restaurant for dinner. The restaurant was a favorite of the carnivorous Donkey Sanctuary staff – carne (beef, pork, lamb, and chorizo) and queso asada with tortillas and salsas. Back in the vans and off to our next hotel in Apizaco, Tlaxcala – nearly midnight again!

Day 11. Thursday 2-2-2017, by Hal Schott

Today we woke up to bright sunshine and cool, dry, thin air – the elevation was over 8,000 feet and Emily and Hal certainly noticed the difference from Veracruz on their morning jogs. We found a new breakfast buffet restaurant – we were always well fed before heading to work in the communities – with the coffee pot being a large clay jug

over the stove. The restaurant was in the middle of a group of small stores and next to it we found large bags of “Michigan beans”, as well as homemade potato chips – quite salty but a great late afternoon snack after sweaty work. Today we headed a bit over an hour to an agricultural community named Alztayanca. Located 8566 feet above sea level the sky was a crisp blue and we had to make sure we applied our sunblock. This community was estimated to have over 6,000 working equids in the area but we only saw about 0.1% on this day. The animals were mostly horses used for agriculture (plowing fields and transporting produce) but equids were also used to gather wood from the local mountain slopes. Not surprisingly, the air was dry at this altitude and the soil was sandy – our work site was a sandy soccer field. As a consequence, we saw

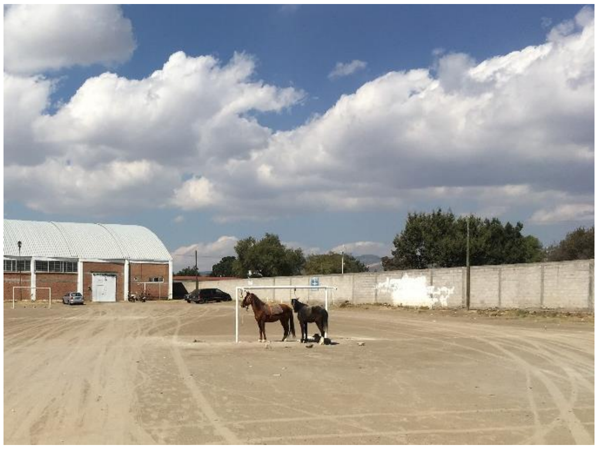
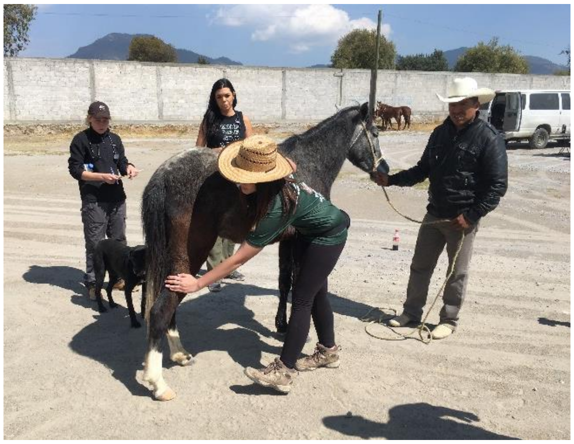

Day 11. Alztayanca, 8566 feet above sea level, was a dry agricultural community where equids are used for plowing fields and transporting produce. Jackie’s hat was quite helpful to block the bright sunshine!

many hard, dry feet today and trimming 25 equids was a real challenge. We also saw a 2-year-old horse with severe contraction of the deep digital flexor tendon in both front legs. The owner asked if could be fixed and, unfortunately, the condition was too severe for any surgical intervention at this age. Amazingly, the horse was getting around fairly well with this severe conformational defect - it really had no other option. The owner

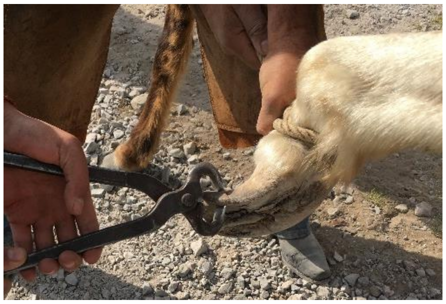
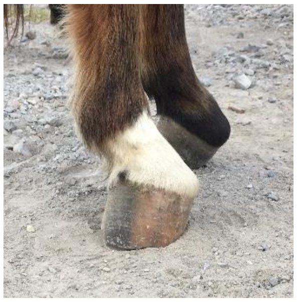
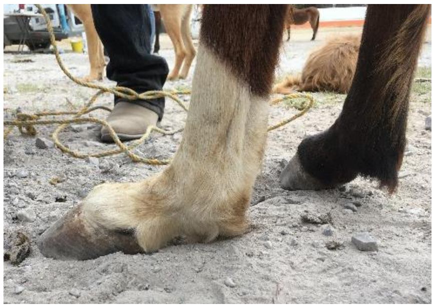

Day 11. Horses in this community had dry, hard hooves that were challenging to trim (left). We also saw a horse with severe club foot of both front feet (center) and another with flexor laxity and dropped fetlocks in both hind legs (right).

also said that this problem had been observed in several of the horses in this community – they were asking about a possible nutritional problem. The horses were in good body condition otherwise making a nutritional problem less likely so we considered a possible genetic problem and recommended against future breeding of the parents of this horse. We also saw the opposite extreme of severe dropped hind fetlocks in another young horse, due to laxity of the superficial digital flexor tendon and suspensory apparatus – cause unknown.

We also performed 15 dentistries, palpated six mares for pregnancy, and performed 1 castration. Dr. Schott helped Bernie pulled a molar from an older horse and an owner presented a horse to Alex and complained that the horse was shaking its head. A deep ear exam revealed a few unexpected small lumps and Alex ended up removing a number of ticks from deep with the ear canals of both ears – hopefully this will help!

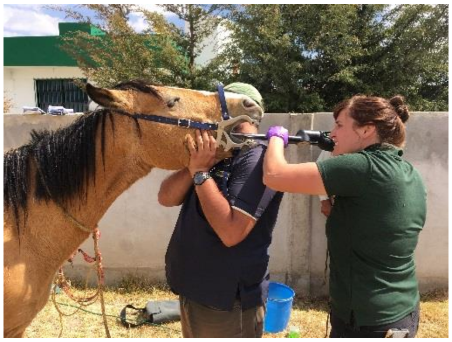
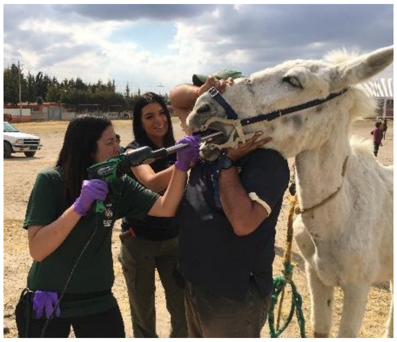
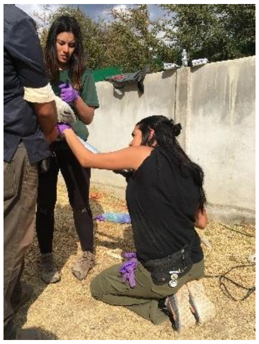
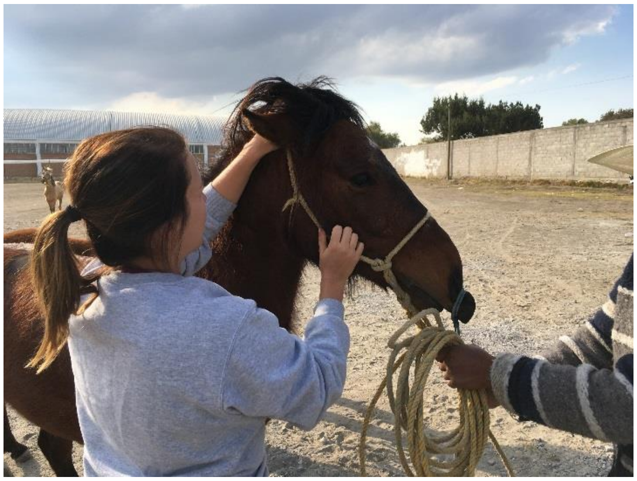
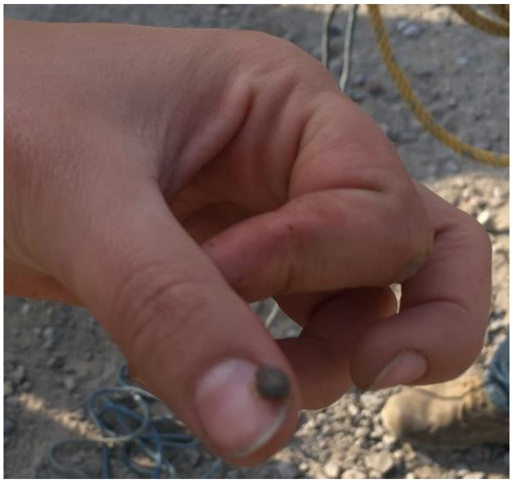

Day 11. Andrea, Jackie, and Sam all gained experience with dentistry today (upper left, middle and right) while Alex helped a 2-year-old horse that was shaking its head due to ticks in both ear canals (bottom pictures).

We worked with a local leader that was essentially the mayor of the community. He was quite professional and had the community members organized for our day's work even before we arrived. This man was only 23 years old while most of the men with the working equids were older. We talked with him for a while about the challenges for young adults in the community – they are attracted away to Xalapa and Mexico City for

better paying jobs and increased social life. This trend poses a significant challenge for sustaining community and agricultural production in Alztayanca and the surrounding communities. There was a large building at one end of the soccer field that had been used for manufacturing local produce for several decades. However, the plant closed more than a decade ago and it was now rented out as a storage facility for paper records from the larger cities! This agricultural area was also experiencing increased use of farm machinery because many of the plots of land were several acres in size. As a consequence of the challenges and changes, the future of this community and its population of working equids is uncertain.

As the day was drawing to a close, community members brought us a large tub of homemade sopes to quell the appetites we had worked up. Another product of this region is Pulque, an alcoholic beverage made from the fermented sap of the maguey plant. It is traditional to central Mexico, where it has been produced for millennia. Pulque is a milky, viscous drink with a sour yeast-like taste when unflavored. After our fill of sopes, the community leader invited us to his house for Pulque tasting. We tried the natural, unflavored version as well as strawberry and coffee flavored batches – another experience we would never have had unless we were welcomed into the local communities. After that we headed back to Apicazo, and feeling somewhat deprived of food we were more used to, we headed to a pizza restaurant for dinner – Lauren said the five cheese pizza was the best she ever had!

Day 12. Friday 2-3-2017, by Jackie Dessoliers

Today was our last day working in rural communities ☹. After another great breakfast we drove to a small town named San Juan Tepulco in the state of Puebla. As we drove into town, a woman announced over a loud speaker that we had arrived and that people should bring their equids to the field where we would be working. On this final day we were joined by Angel, another farrier that works for the Donkey Sanctuary and a welcome addition to spare our tired backs. San Juan Tepulco is one of the original communities that the Donkey Sanctuary started working in more than 20 years ago. The community is estimated to have about 1,200 donkeys, mules, and horses, although creating and maintaining a census of the equids had been challenging. The donkeys are mostly used to carry wood and corn from La Melanche, a mountain 2 hours away. In this community, women and children work with the donkeys while the men travel to Mexico City for daily construction work. Many of the women also carried bambinos in sarong like garments and also had colorful ribbons interspersed in their braided hair.

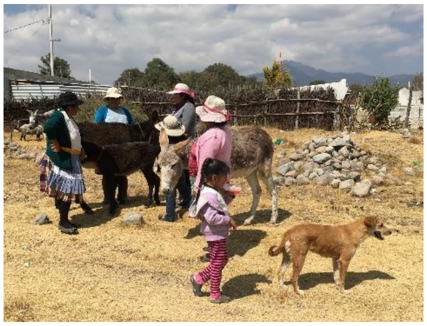
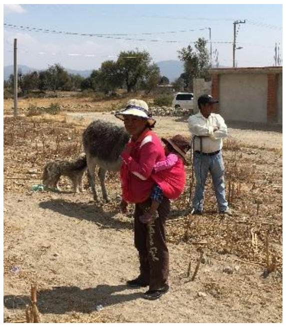
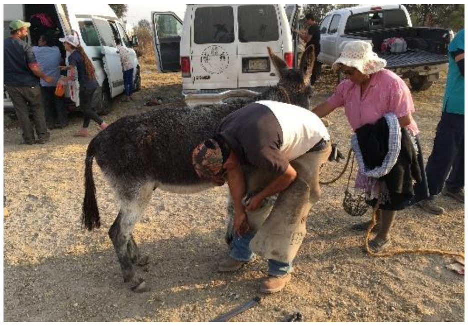

Day 12. Our arrival in San Juan Tepulco was broadcast by loudspeaker and women and children soon arrived (left) with their working equids (and bambinos on their backs [middle]). Today we were happy to be joined by Angel, another farrier that works for the Donkey Sanctuary (right).

On this final day, Humberto brought out a secret weapon – small laminated cards that were given to the animal owners for their turn! Our day consisted of 20 foot trims, 15

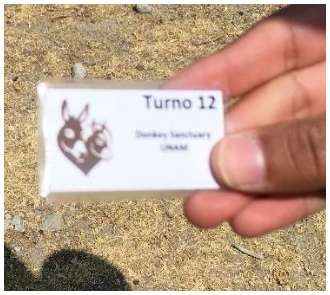

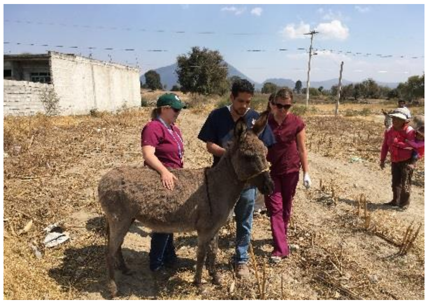
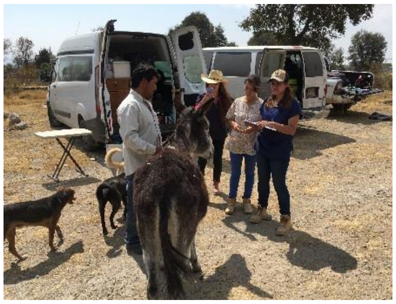

Day 8. Humberto's secret weapon – laminated cards to let equid owners know when it was their turn for service (left). Lauren and Olivia are examining a burro with Dr. Eduardo Santurtun, head of the Donkey Sanctuary in Mexico (middle), and Monica, Anaid, and Aurora interview a donkey owner (right).

dentals, 1 castration, and deworming (again, we were all out of vaccines by this time). Emily and Bernie also examined a coughing horse that most likely had recurrent airway obstruction (heaves) and provided a good teaching opportunity to review this chronic airway disease (the dusty, high altitude [about 8,500 feet] environment was likely a nonspecific exacerbating factor).

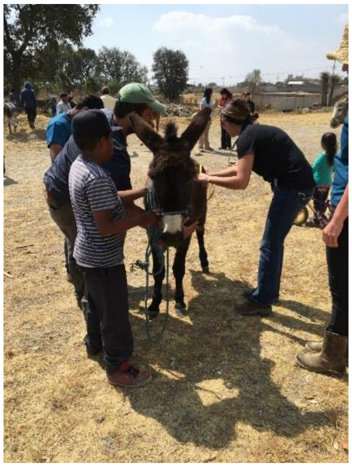
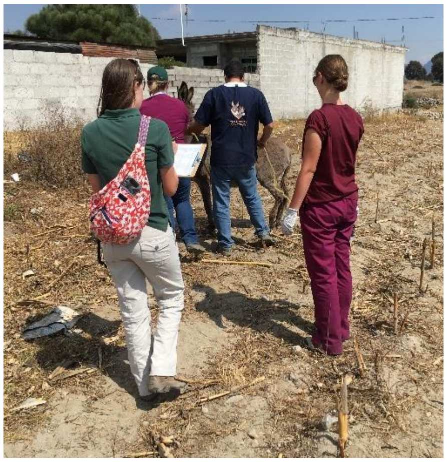
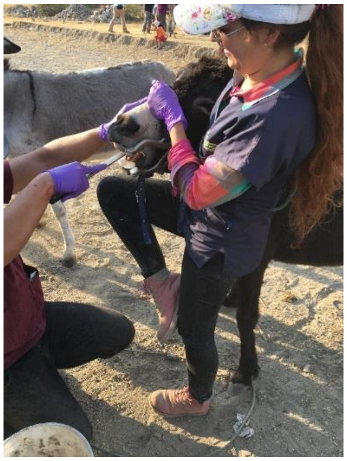

Day 12. Bernie and Alex measuring girth circumference to estimate bodyweight of a donkey (left), Emily keeping records of a physical exam (middle), and Monica's unique method to help hold up a donkeys head during dentistry.

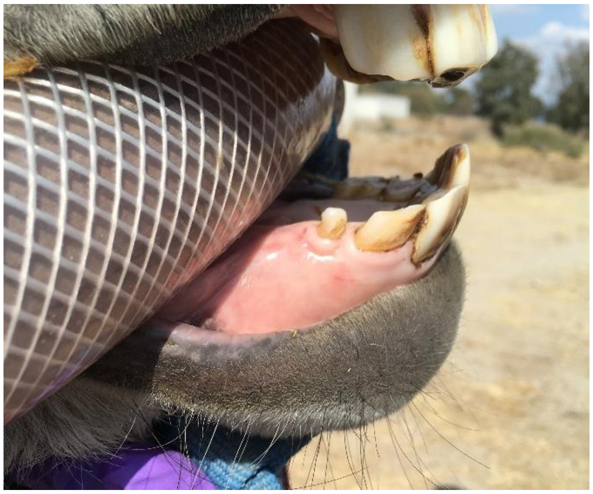
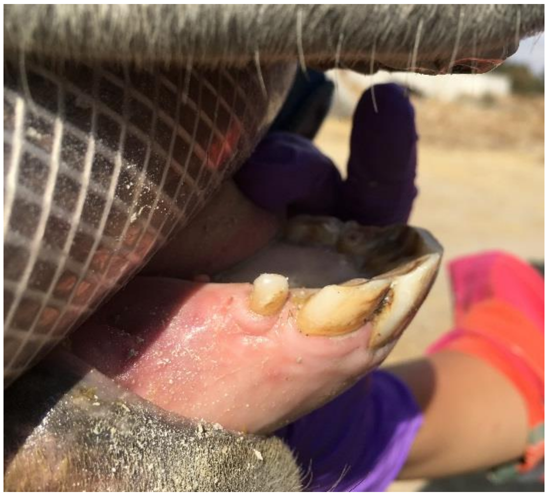

Day 12. A donkey with overgrowth of bottom incisors due to a "parrot mouth" or overbite before (left) and after correction (right).

Towards the end of the day a little boy walked into the work area leading his donkey. It was a priceless sight that embodied how important the working equids are to the families that own them and how these animals really are family members. It was a great image to remember all of the time we spent in all of the rural communities.

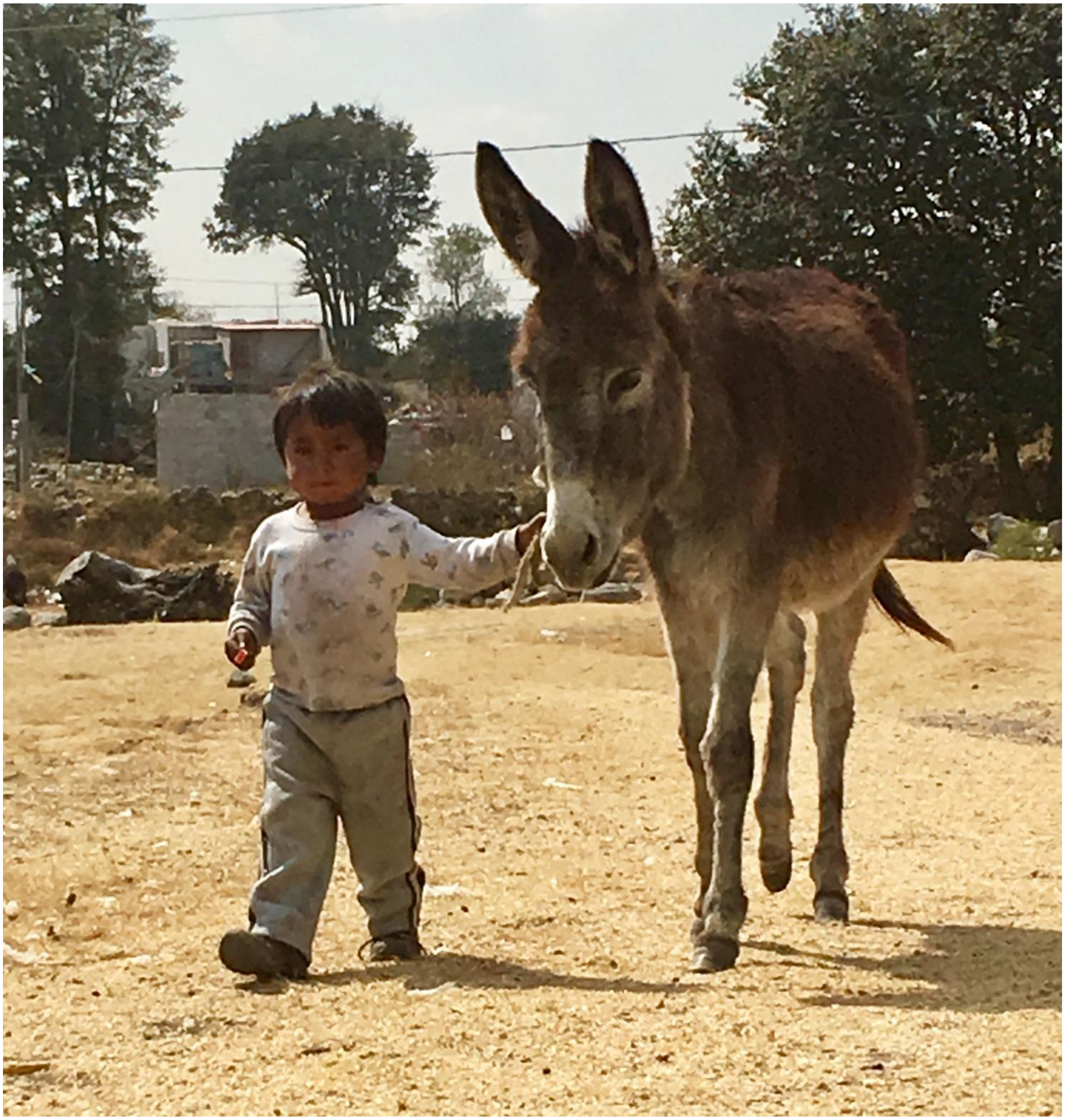

Day 12. A little boy with his donkey – a picture is worth a 1000 words!

At the end of the day we packed up our supplies and headed to a local restaurant where we said goodbye to our fellow UNAM interns and students following the meal, after they gave some gifts of coffee and tequila for Drs. Schott and Bunn. Then it was back in the vans for a long ride to Mexico City where we arrived at our final hotel a bit after midnight – thanks to the General and Luis for driving us safely well over 1000 miles over the past couple of weeks.

Days 13-15. Saturday-Monday 2-4 to 2-7, 2017, by Lauren Kustasz

On Saturday morning we began our day with a tour of UNAM's Mexico City campus. The General drove us to campus and showed us around a bit and then took us down to the Donkey Sanctuary office in UNAM's Veterinary School. There, José gave us an in depth description of where the Donkey Sanctuary does its work all around Mexico and how they decide which communities to work with. It was interesting to see behind the scenes of the Mexico Donkey Sanctuary program and to learn more about where the organization works across Mexico and their many logistical challenges.

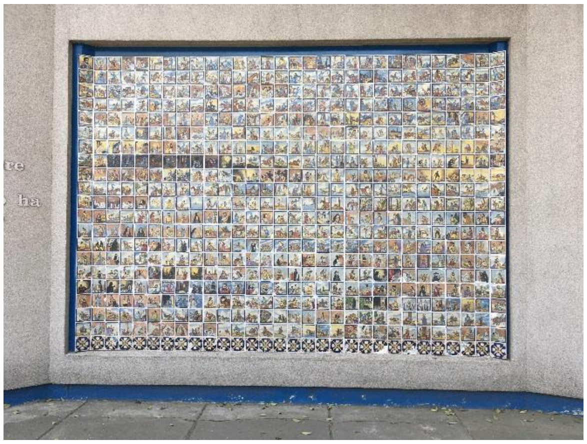

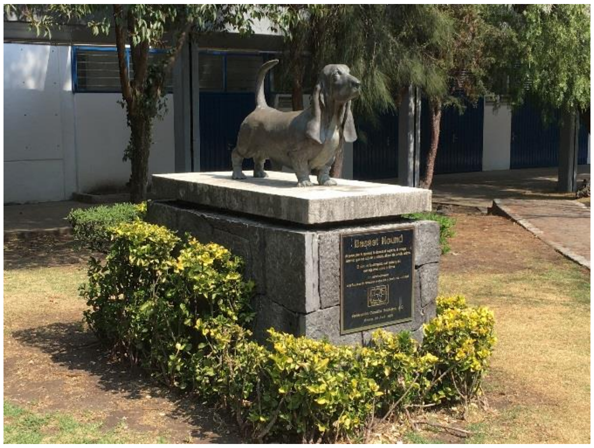

Day 13. A large tile mosaic at UNAM's Veterinary School detailing the history of the Hildago (nobility) in Mexico over several centuries (left) and a Bassett Hound, one of the several animal statues in the courtyards around UNAM's Veterinary School (right).

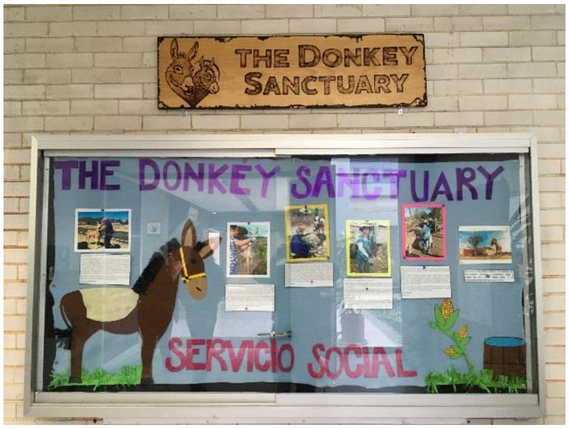

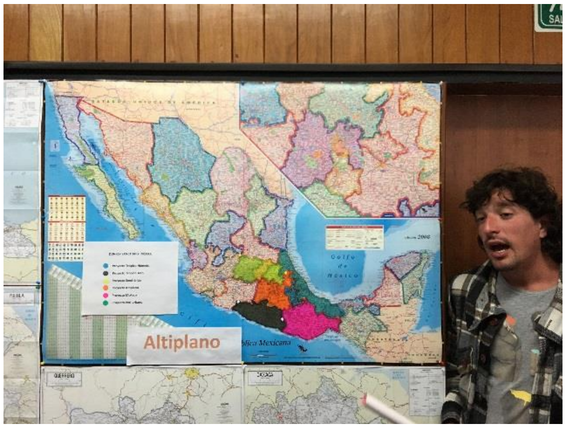

Day 13. A display made by UNAM students outside of UNAM's Donkey Sanctuary office (left) and José showing us a map showing the five states (color filled) of south central Mexico where the Donkey Sanctuary has active projects (right).

After this, we met up with Bernie (UNAM equine hospital intern) who showed us around the equine hospital. The hospital was similar to MSU's equine hospital with two surgery suites (one for colic surgery and the other for arthroscopy). Both surgery suites had similar padded rooms for induction of anesthesia and recovery, as well as a similar hoist

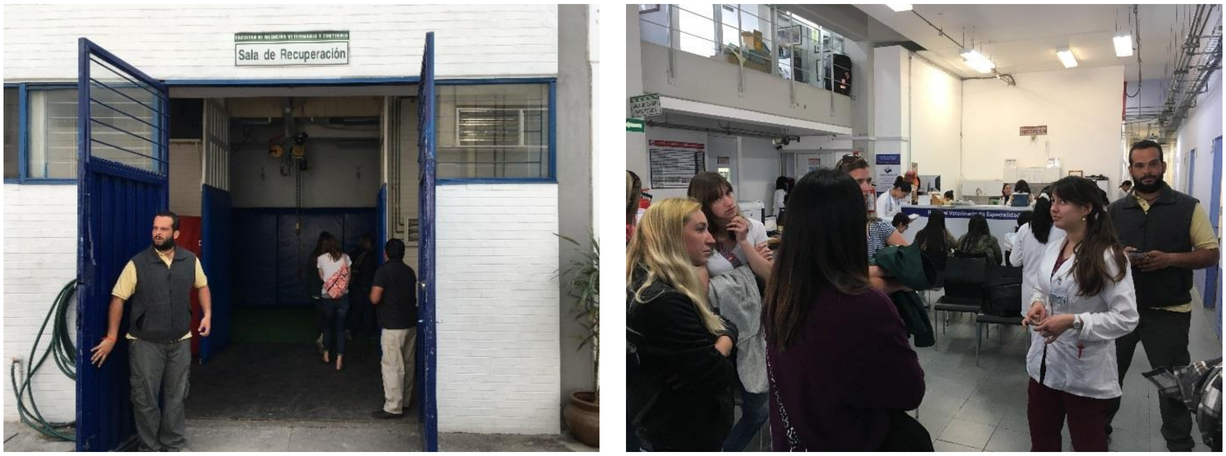

Day 13. Bernie, a UNAM equine intern, gave us a tour of the UNAM equine hospital (left) and after that one of the small animal residents met us at the busy small animal reception desk to give us a tour of the small animal clinic (right).

mechanism to lift horses into the surgery rooms. The main hospital had fewer stalls than MSU's hospital with only one main ward, although there was also an infectious disease ward that we did not visit. Most of the stalls in the main ward were occupied by patients. One of the equine residents was kind enough to give us rounds about the patients in the hospital – the level of care was very similar to what we do at MSU. Patients included a dressage horse that had received a corneal graft, another performance horse that had just gotten a cataract removed, a horse recovering from enterocolitis, another one recovering from a sinus surgery, and an orphan foal. This was a busy time in their equine hospital, compared to normal, because we learned that their case load is only about 300-400 patients per year (compared to about 1,500 per year here at MSU). They also had a horse and a donkey living there that had been rescued from the slaughter house that were waiting to be adopted.

After touring the equine hospital, we wandered over to the small animal hospital to see if we could find someone to give us a tour. One of the small animal residents was nice enough to give us a quick tour of the hospital, even though it was packed with patients and busy. Although the small animal hospital at UNAM was small and crowded, it had all of the major specialties, even rehabilitation. It was nice to see that they had a separate area for dogs and cats, something that MSU is currently building. Also, it was interesting to learn that they have 22 residents at all times (three for each specialty)! Another difference from MSU was that their emergency service only takes patients that have been seen previously at their hospital, meaning they already have a doctor-client-patient relationship for all animals presented on an emergency basis.

Next, Dr. Mariano joined us and gave us a tour of UNAM's central campus. We had a great snack from a bicycle vendor selling tortas overlooking the large central campus lawn area, where many student events occur. This was followed by some tasty fruit ice cones! Adjacent to this square was the main library, a seven story building covered with

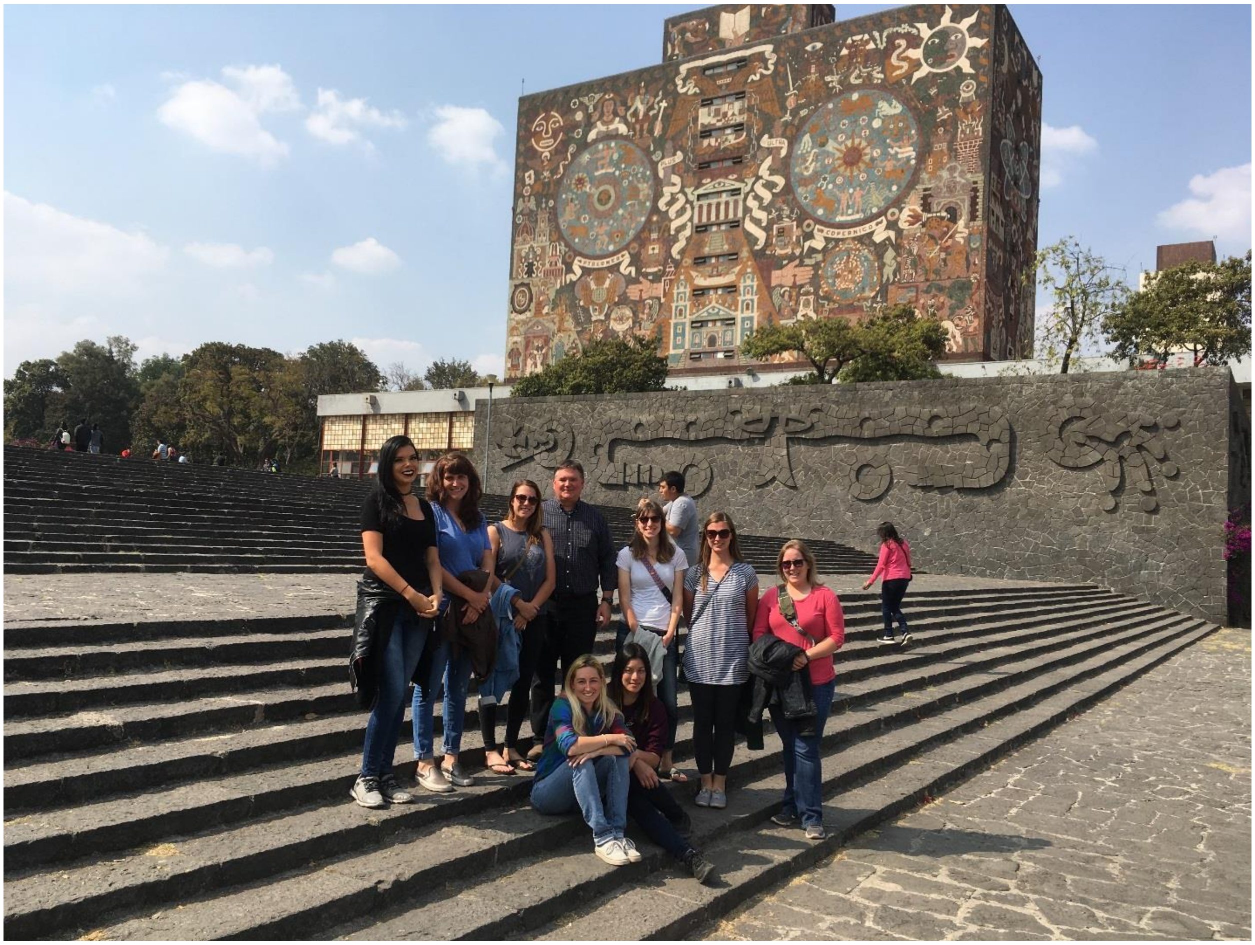

Day 13. Our MSU team touring UNAM with the main library covered with a mosaic by Diego Rivera in the background.

a beautiful tile mural by Diego Rivera. From this main square we then walked over to the old Olympic Stadium where UNAM's soccer and American football teams play.

Once we were done touring campus, the General and Mariano took us to Coyoacán, a cute little suburb of Mexico City near the UNAM campus. We spent about an hour shopping for souvenirs in a craft market and then all went to a restaurant for dinner. The restaurant had the most amazing melted cheese to put on tortillas! On the way to the restaurant, we saw several stores making churros so we had to stop at one after dinner for dessert, they were delicious and a perfect ending to the day.

Sunday morning was a free day to rest and we had a bit of a later start. Five of us decided to go tour the Museum of Frida Kahlo. It was also nearby in Coyoacán and the museum was packed, so we had to wait a bit to get inside. The museum was built in Frida's home, La Casa Azul (The Blue House). The museum was beautiful and I learned a lot about Frida's life, her accomplishments, and her love for her country's traditions and culture.

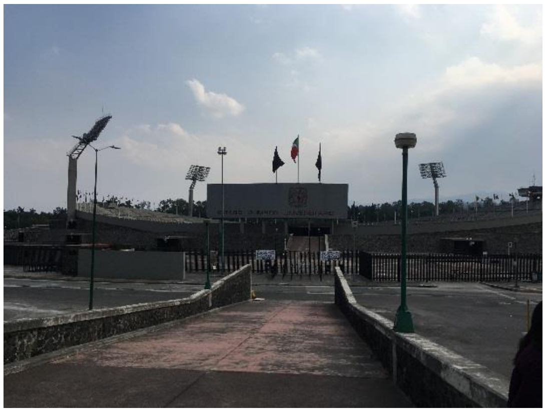
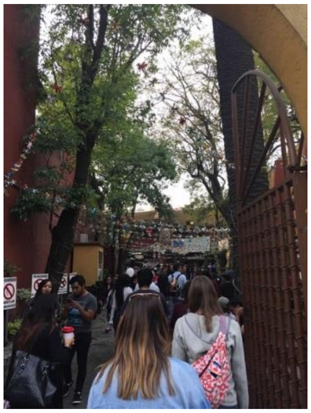
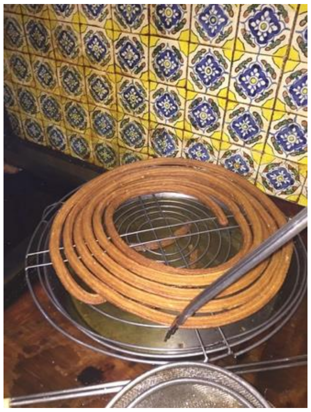

Day 13. The Olympic Stadium (left), entrance to the craft market in Coyoacán (middle), and fresh made churros for dessert (right).

After visiting the museum, the five of us decided to walk around the market that we went to the day before. We bought some delicious salsa and then decided it would be nice to sit down at a restaurant for a while and relax. We found this really cute cantina that looked out over the city square. We each had a drink or two and some nachos. It was a really nice afternoon. When it hit evening time, our group headed back to our hotel where we met up with Dr. Schott, Dr. Mariano and Dr. Elena to watch the Super Bowl in the hotel bar. We were all rooting for the Falcons, so the end of the game was a disappointment for all of us, but it was an exciting game to watch!

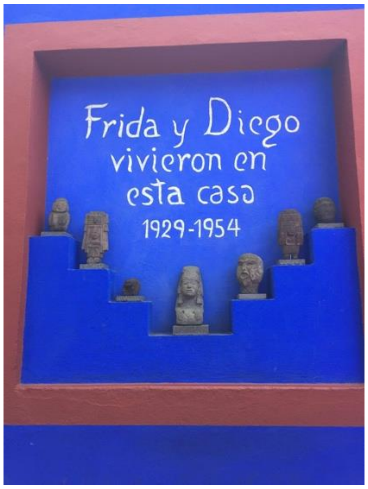

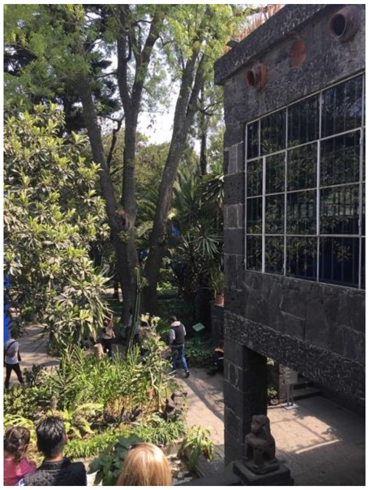
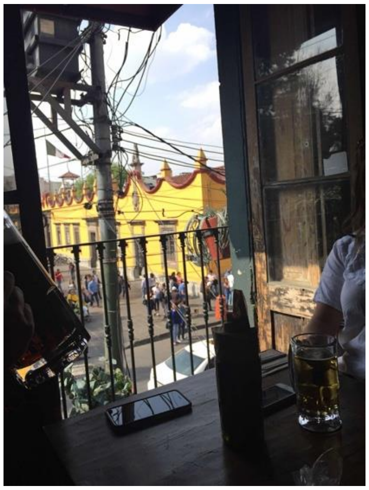

Day 14. The Casa Azul (Frida Kahlo museum) entrance (left), view of the courtyard of Casa Azul (middle), and view of Coyoacán from a cantina (right).

Monday was our last full day in Mexico. In an effort to summarize our experience and create a sense of closure, the entire gang (minus Dr. Elena, the UNAM interns, and a few of the UNAM students who were busy working in the hospital) met up to discuss our favorite memories and to reflect on what we had both accomplished and learned during our time together. We sat outside the new contemporary art museum at UNAM for our discussion. All in all, we had examined, vaccinated, and dewormed over 800 working

equids in the eight rural communities we visited from Veracruz, through Tlaxcala, and back to Mexico City. We trimmed the hooves of more than 130 equids, we performed 75 dentistries, we palpated 30 mares for pregnancy, and we castrated 20 equids. In addition, we treated many wounds and helped care for other problems. More importantly, we gained first-hand knowledge of the vast need for care of working equids and developed a deep respect for the Donkey Sanctuary mission. We all felt truly blessed at having helped many hard working Mexican families while at the same time making new friendships with all of our UNAM and Donkey Sanctuary colleagues. The discussion was a nice way to end the program; however, it was sad to know we were about to say goodbye to all of our new friends. The good news is that we have been invited back in 2018 with a new group of MSU students and faculty! We ended our morning with a final group picture and we departed ways with some of the crew while the rest of the group headed to downtown to see the heart of Mexico City.

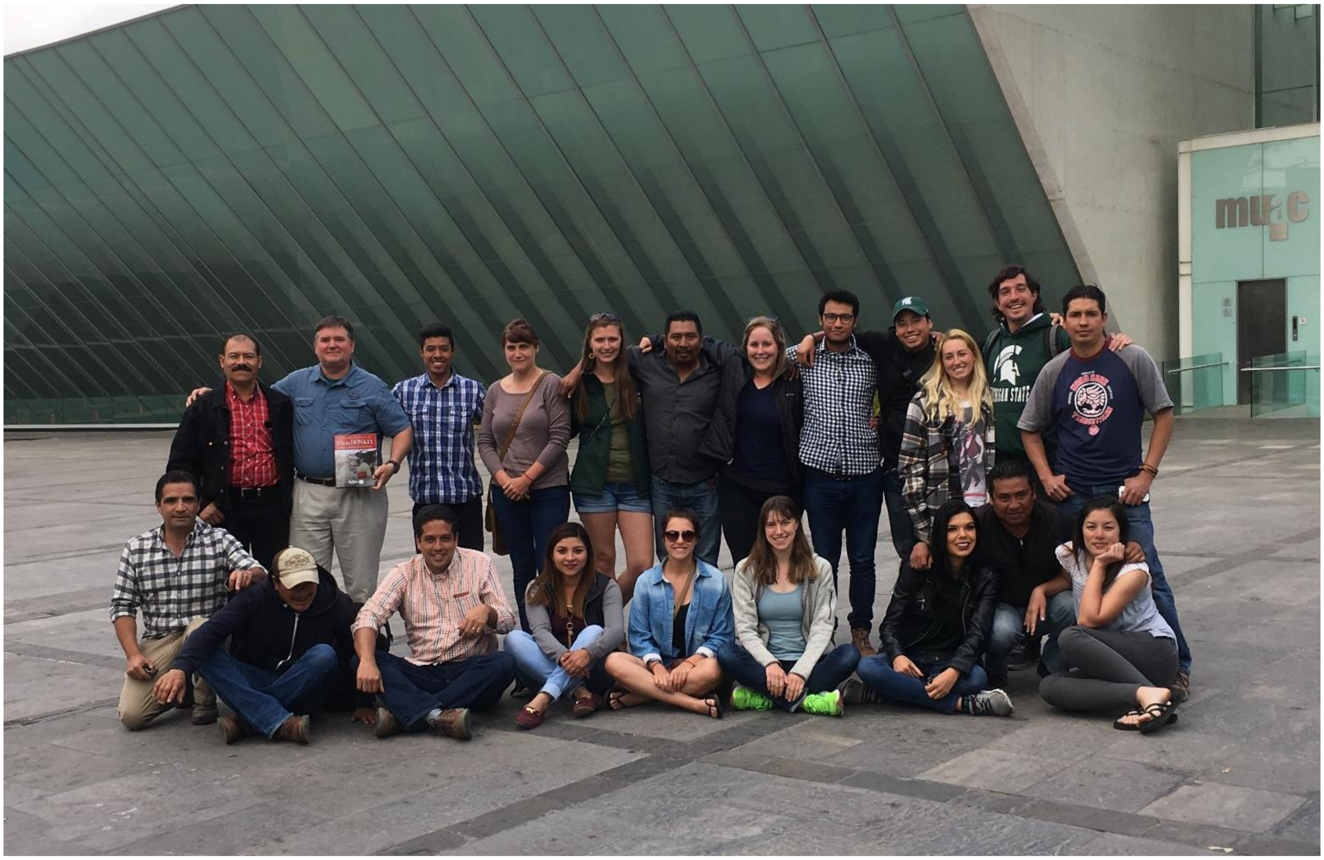

Day 15. Our **Equine Welfare in Practice 20**17 team: front row (left to right): Dr. Mariano, Angel, Dr. Eduardo, Aurora (UNAM student), Alex, Emily, Sam, the General, and Jackie; back row (left to right): Luis, Dr. Hal, Carlos (UNAM student), Andrea Lauren, Mauro, Olivia, Humberto, Dr. Carlos, Mackenzie, José, and Dr. Arturo.

Downtown, we met up with a large animal internal medicine veterinarian from UNAM (Dr. Maria Masri) and her husband and ate an absolutely delicious lunch at a beautiful

restaurant overlooking the Templo Mayor archeological site. Then we headed back down to the Zócalo or Plaza de la Constitución (main downtown city square), the original main ceremonial center in the Aztec city of Tenochtitlan. We visited the cathedral and watched as a huge Mexican flag was lowered in the center of the square by soldiers of the Mexican army. We left downtown around 7 pm and drove around Mexico City for a while with Dr. Mariano as our tour guide, seeing the Ángel de la Independencia statue given to the Mexican people by France in 1910 with gold Lady Victory at the top, and then headed back to the hotel to pack up and get ready to return to Michigan early the next day.

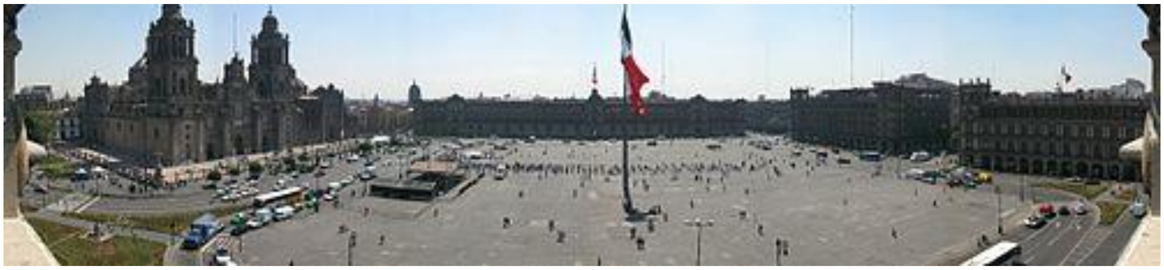

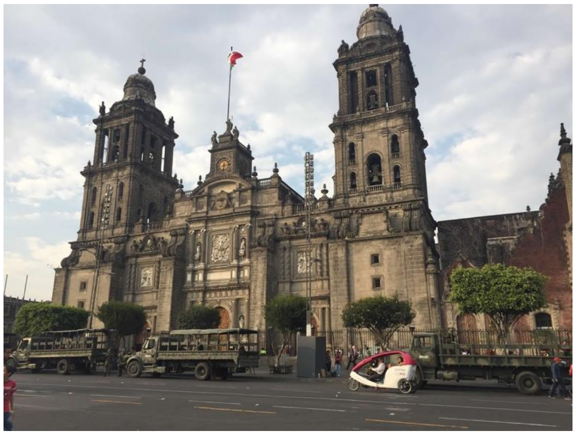

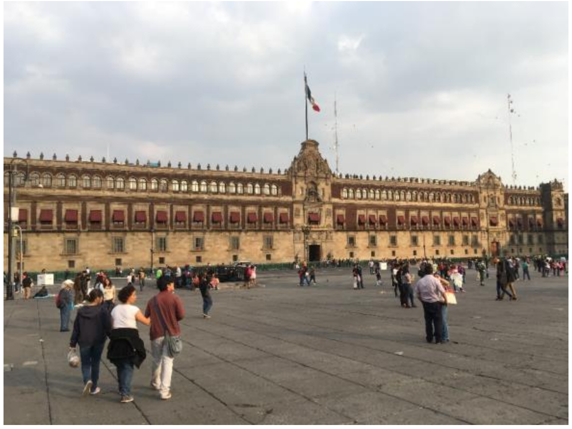

Day 15. The Zócalo or the Plaza de la Constitución, the main downtown city square (upper panel), flanked by the Metropolitan Cathedral of the Assumption of the Most Blessed Virgin Mary into Heaven Cathedral to the north (lower left) and Palacio Nacional to the east (lower right).

Day 15. The Ángel de la Independencia statue, given to the Mexican people by France in 1910 with gold Lady Victory at the top, was one of our last sights in Mexico City.

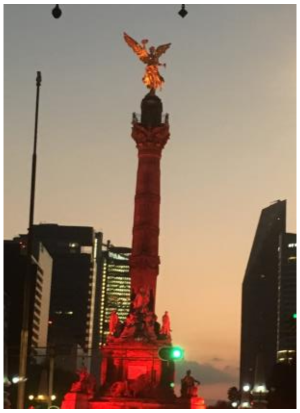

THE END
