# Supplementary material for: Equine Welfare in Practice: A Collaborative Outreach and Education Program with Michigan State University, Universidad Nacional Autónoma de México, and Universidad Veracruzana
Source: Animals (Basel). 2019 Apr 13;9(4):164. doi: 10.3390/ani9040164 (PMC6523699; doi:10.3390/ani9040164)
Supplement: Supplementary file 1 [file animals-09-00164-s001.zip › animals-471606-supplementary/MSU-UNAM-UV Equine Welfare in Practice - blog - 2019.pdf]

Equine Welfare in Practice 2019 – Student Blog

Day 1 - Saturday 1/19/19, by Rachel Baumgardner - The Journey to Veracruz

We were all excited to begin our journey to Mexico. My peers and I spent a bit of time practicing basic phrases in Spanish and trying to cram 2 weeks of clothes and personal items into one carry-on bag. I ended up booking a different flight than everyone else, which ended up working in my favor. We left the Midwest just as a horrible cold front hit, causing strong winds to come from the north to bombard the gulf. My flight landed in the morning before the winds really hit. Due to a delay getting medications and supplies through Customs in the Mexico City airport, most of my classmates and my professors missed the connecting flight to Veracruz. The next flight they were able to take couldn't land in Veracruz due to the high winds, and they spent the night back in Mexico City, after a detour south for landing and refueling in Villahermosa, Mexico.

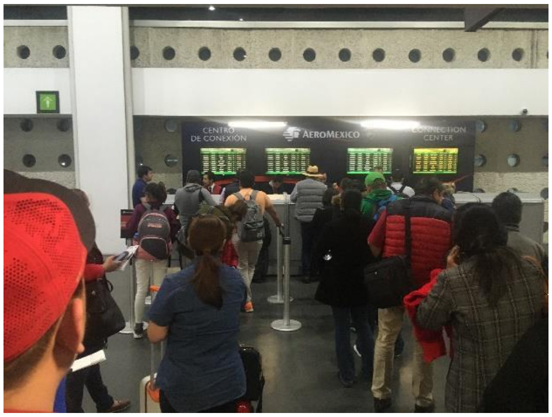

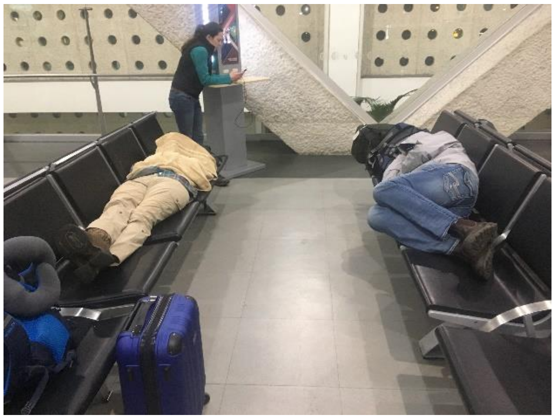

Rebooking our flight to Veracruz once again and Drs. Esser and Kinsley trying to catch 40 winks in the Mexico City airport

While my travel companions were having a less than ideal time getting to Veracruz, I enjoyed a day of beautiful weather along the Atlantic coast in Boco del Rio, at the southern end of the municipality of Veracruz. I promptly got a sunburn but I was so Vitamin D deficient that I didn't notice. I swam in the ocean for the first time in my life. I read a book on the beach, followed by a siesta. I awoke from my siesta to texts from my classmate, Roya, who had made the connection from Mexico City. She made her way from the airport to La Costa Inn with Drs. Alejandro (Alex) Estrada Coates and Miriam Alva Truijillo, who are the equine faculty members at the University of Veracruz that are collaborating in our 2019 Equine Welfare in Practice Clerkship. We enjoyed a nice dinner at a restaurant near the hotel and got to know our Mexican mentors.

Day 2 - Sunday 1/20/19, by Rachel Baumgardner – exploring Veracruz

On Sunday morning, the rest of our MSU group finally made it to the hotel, still tired from their short overnight stay in the cold Mexico City airport. After a shower, a short rest, and lunch, the team was ready to explore and headed out to see San Juan de Ulúa, a historic port and fortress built in 1565 using blocks of coral cut from the

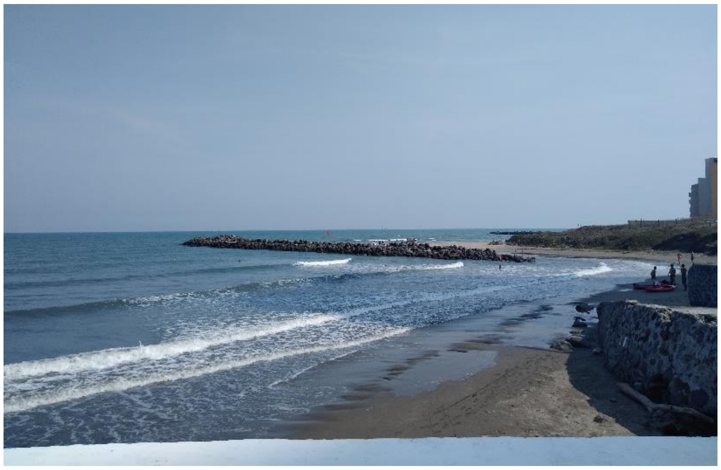 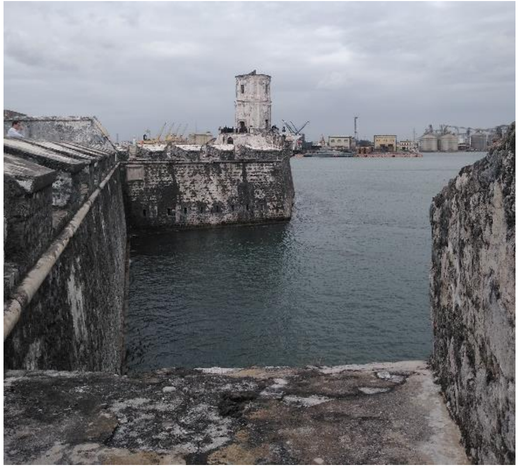

My view from the sidewalk of the beautiful beach in Boca del Oro and the fort San Juan de Ulúa,
a popular historical site and the last stronghold held by Spain during Mexico's war for Independence.

seemingly endless supply in the Veracruz Bay. The fort was the site of multiple clashes between Spain and Mexico, and was the last stronghold held by Spain during Mexico's maintained as a symbol of the perseverance of Mexico in gaining their Independence from Spain. We all enjoyed exploring the fort and its prison chambers (which were fairly dark and scary) for a few hours. After our tour we regrouped at the La Costa Inn, purchased supplies from the store near the hotel, and walked to a nearby restaurant (Los Giros) for a tasty Mexican dinner across the malecon from the Atlantic Ocean.

Day 3 - Monday 1/21/19, by Rachel Baumgardner – the Clerkship starts!

On Monday morning we had breakfast at the hotel and loaded up in the vans and truck for a caravan to the University of Veracruz's Equine Hospital. Drs. Alex and Miriam gave us a tour of the examination room, patient stalls, induction room, and surgical suite. We were quite impressed with the facility that has been open for almost 2 years where the veterinarians have already seen a number of patients and completed a number of surgeries. The hospital is staffed by the two faculty veterinarians and University of Veracruz students that rotate through during their final year of training. These students are in charge of the care of the animals 24/7. They have a few "resident mascots" at the hospital, including two miniature horses (Bandito and Isis), an iguana found injured by a hawk that was undergoing rehabilitation, and a mammoth donkey. The facility also houses about a dozen mares that were all currently in foal with the donkey so they are expected a crop of mules foals later this year.

The surgical suite at the University of Veracruz's Equine Hospital

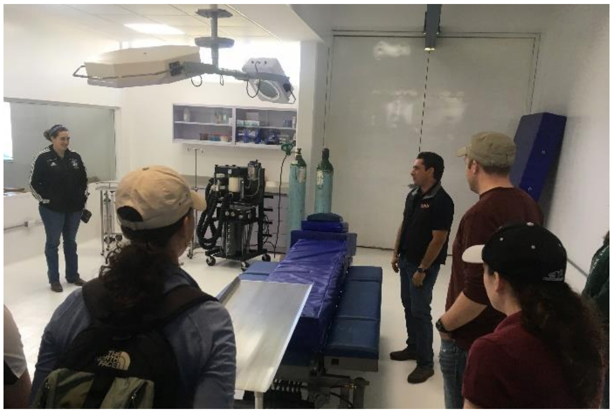

While at the veterinary hospital, Mauro from the Donkey Sanctuary talked to us and demonstrated the important differences between handling horses, donkeys, and mules, with Dr. Jose Casillas as our interpretter. Many of us had experience with horses, but few of us had worked much with either donkeys or mules. They are quite different, despite being closely related! We had a great time attempting to take a heart rate on the resident mule, who was young and had not been worked with much – I learned first-hand the reach of it's hind feet. I found the lesson enlightening, and enjoyed trying to develop trust between a young, unbroken animal and myself. This introduction made me look forward to the rest of our trip, in working with many different equids across many different communities. After a lunch of homemade tamales at the Hospital, we once again and loaded into the vans and trucks and started our 4 hour trek south to UNAM's Los Tuxtlas Research Station in the Veracruz rainforest.

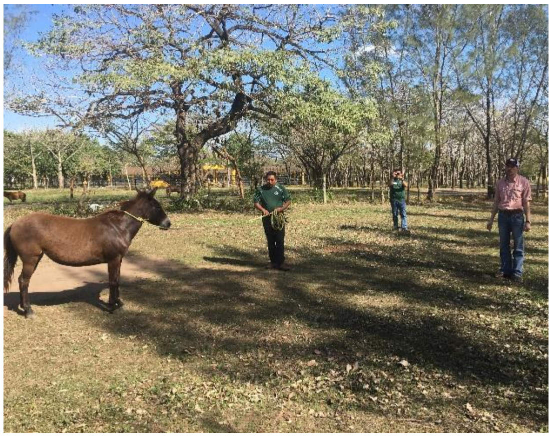

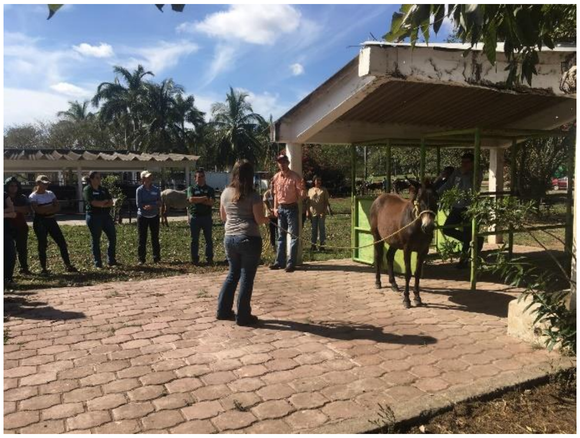

Mauro, an expert in equid behavior with the Donkey Sanctuary, providing a demonstration (with translation by Dr. Jose Casillas) of the differences in behavior between horses, donkeys, and mules and Katelynn trying to implement what she learned about mules.

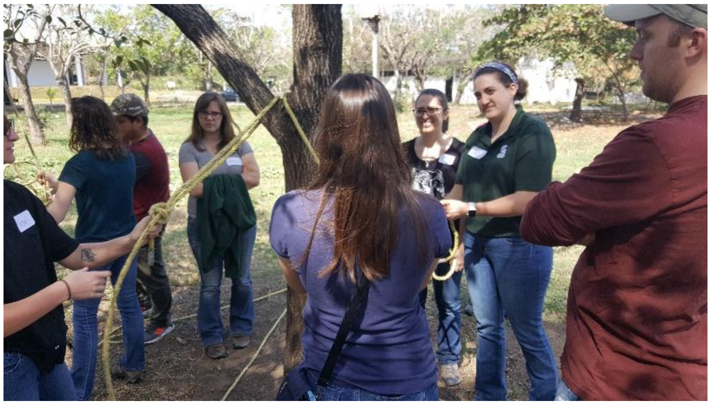

Practicing tying bowlines and quick-release knots for securing equids at the University of Veracruz Equine Hospital.

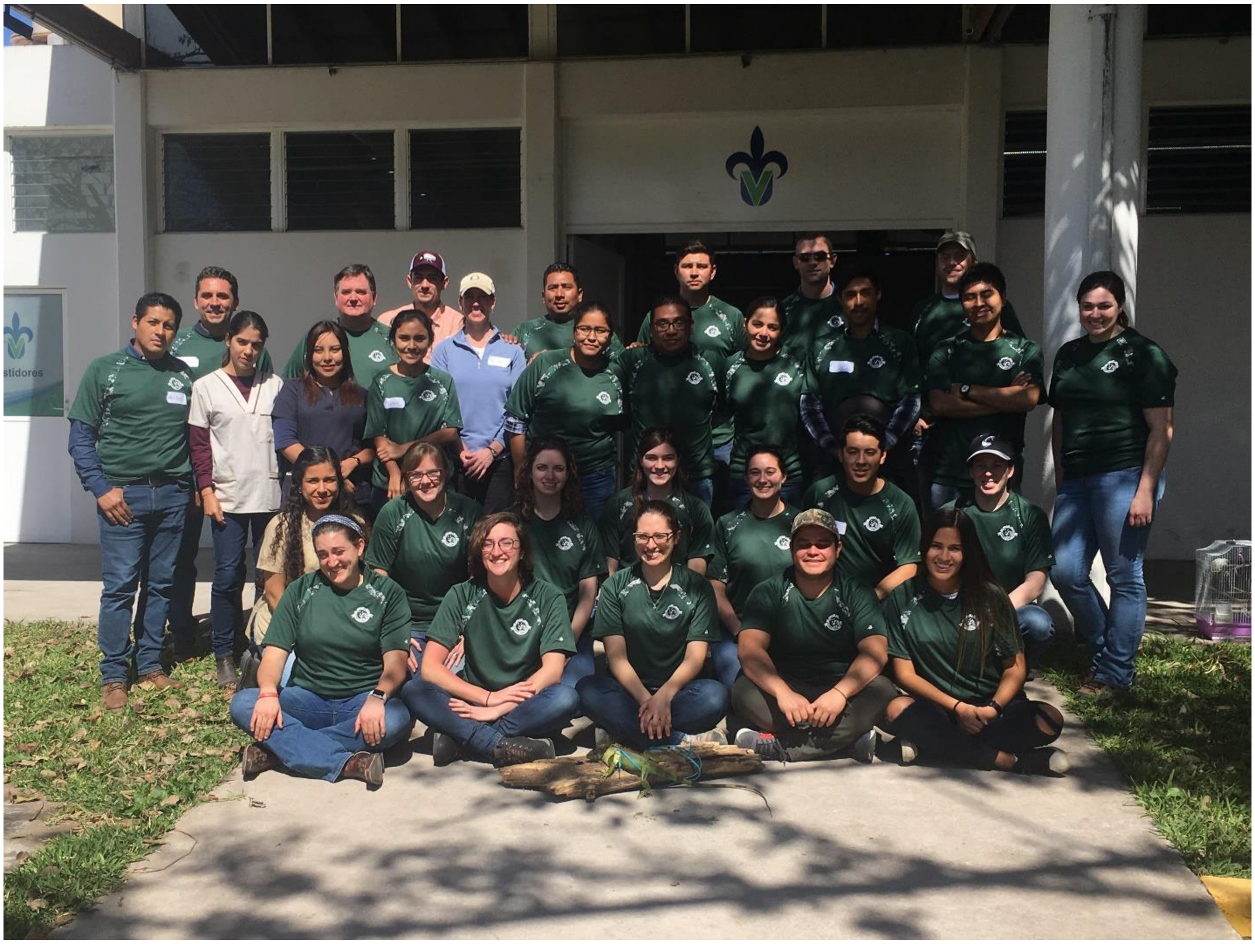

The faculty of UV, students of UNAM and UV, MSU faculty, and MSU students in front of the UV teaching hospital.

We arrived at Los Tuxtlas in the late afternoon and were introduced to Rosamond Coates Lutes, Dr. Alejandro’s mother and Director of the UNAM Research Station. She gave us a short overview of the history and many ongoing projects of the station, with special emphasis on poisonous snakes in the rainforest, especially for Dr. Kinsley. After that we went on a guided walk through the rainforest during which we heard and then saw some of the resident howler monkeys, while avoiding grabbing onto the trunks of

the many spiny trees lining the trail. After arriving back at the station it was time for dinner in the Comedor and a few hands of Euchre and other card games in the Library before calling it a night.

Day 4 - Tuesday 1/21/19, by Katelynn Youatt & Mariela Fernández

There are some experiences that transcend the ordinary. Getting to provide veterinary care for rural communities in Mexico has undoubtably been one of those experiences for me. After our eventful travel to Veracruz, we ultimately ended in the region of Los Tuxtlas. Here we are staying at the UNAM Biological Station in the rainforest. It is not hard to feel that you have crossed parts of the world when there is an Iguana sitting outside your window, and Howler Monkey cries greet you in the morning. The station will be our base for the next week as we venture out to local communities; providing vaccines, minor surgical procedures, hoof care, and dental care to the working equids of Mexico.

The fourth day of our trip has also been our first day visiting a local community. **Balzapote** is a community of approximately 650 people in the region of Los Tuxtlas. The community's economy relies heavily on fishing and cattle raising, with corn being their primary product. Because of its rural nature, up to 50% of the community members own either a horse, donkey, or burro – with a majority of households owning a horse. These animals are used primarily for cattle work and transport; although a few are pets. Because of this, healthcare for these animals greatly affects not just their welfare, but the lives of the people that own them as well.

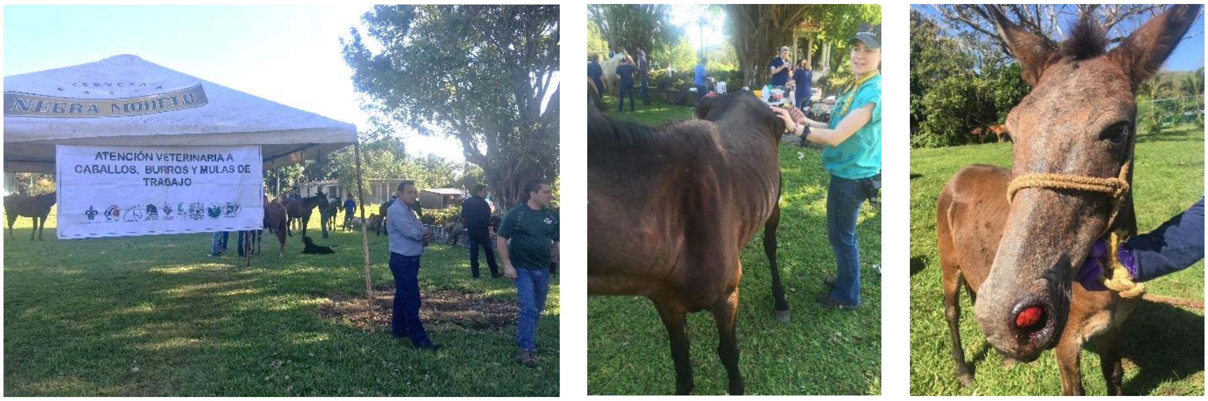

Our project's welcome station (left), Skylar examining a skinny horse (acquired by the new owner 6 days before, most horses were actually in good body condition, middle), and an unusual nasal mass (that was surgically removed later in the day, right) in an older mule that was still working every day.

Our day started when we were greeted by a small boy (perhaps 6 years old) riding his *burro* – or donkey – into our work station. His arrival heralded in what became over 80 horses, donkeys, and mules that day that traveled to see us for veterinary care. Upon arrival all animals were assessed for any initial needs. They received a physical exam, and were evaluated for what other services could be provided. All animals were eligible for vaccines, vitamins, and de-wormer. In addition to this, they could be sent for farrier

work (trimming the hooves), castration of intact males, dental procedures, and reproductive soundness exams. Every owner coming in also was able to benefit from education about saddle fit, and husbandry of their animal. It was really exciting as a student to work on anesthesia, and do both dental work and castration surgery. For many of us, it was our first time doing these procedures, and we were able to work on them confidently with the direction of our mentors.

In addition to great care, working with the students of the University of Veracruz and UNAM provided an extra opportunity to work on both our communication and Spanish skills. Pairing up, we worked as teams to properly communicate with clients, and were able to learn a lot from each other. It is fun to know that no matter what the language, there are common goals and abilities that we can come to the table with as veterinarians.

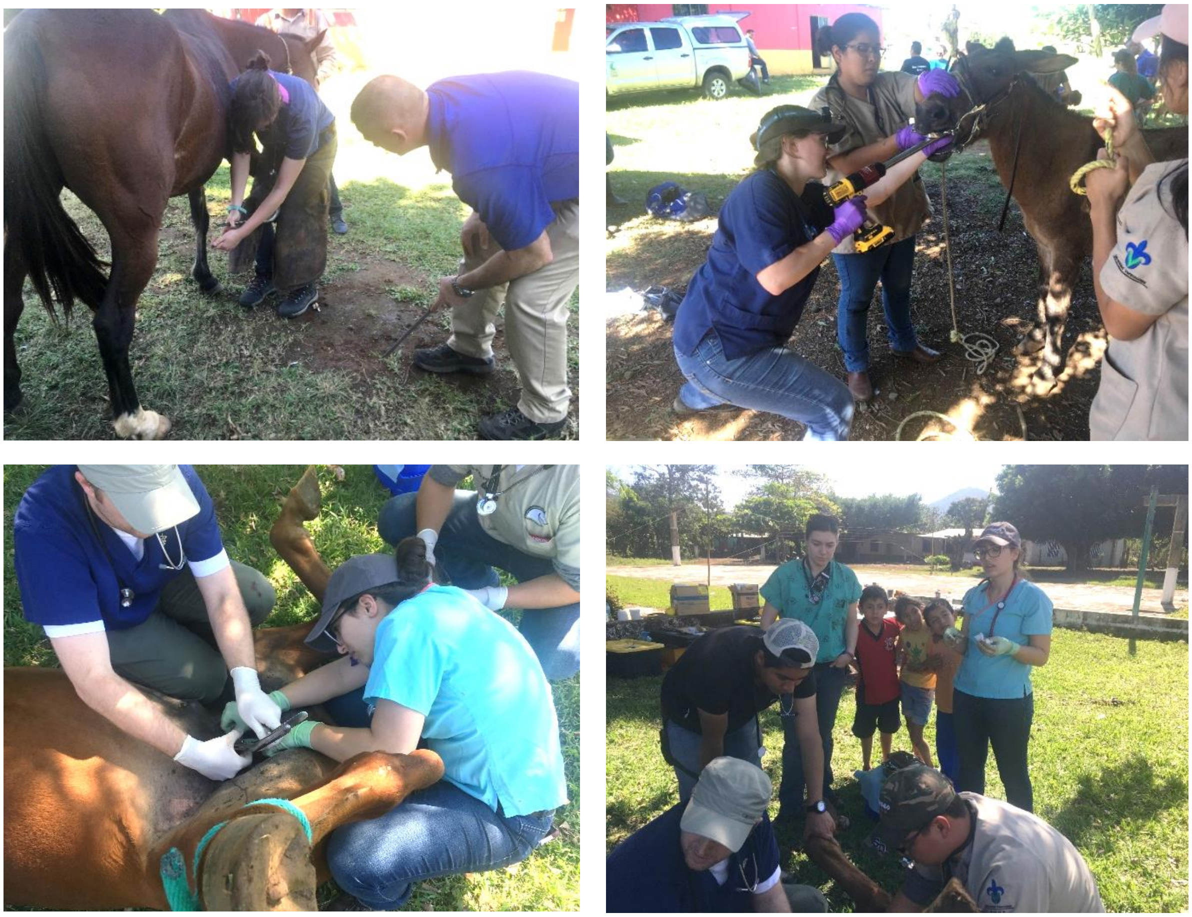

Dr. Matt Davis (one of the Michigan equine vets that volunteered to join our team for the first week of the clerkship) instructing Rachel in farrier work (upper left), Katelynn floating a horse’s teeth (upper right), Dr. Kinsley talking Roya through a castration (lower left), and Skylar and Roya practicing their Spanish with kids from the community that were fascinated with the castration being performed by Dr. Kinsley and Gerardo

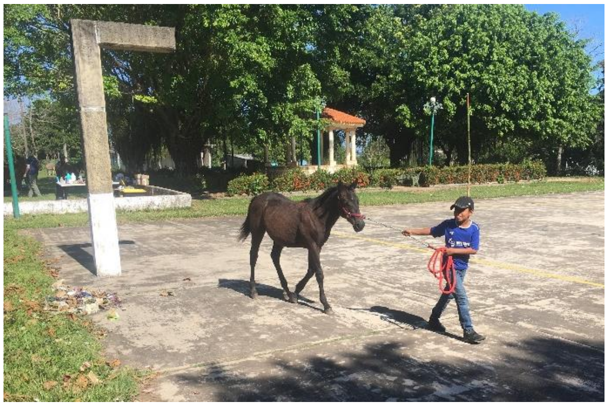
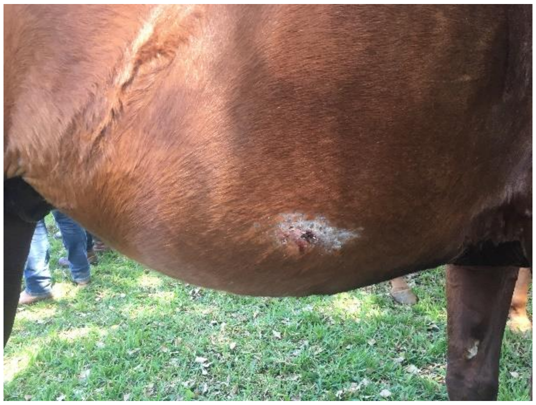

Owner education is an important component of our clerkship: reaching out to children and letting them see how veterinary care may be effectively and safely provided to their animals is important to shape future care of working equids in their communities. In contrast, it was more challenging to try to convince the owner of the horse on the right to stop using spurs that created this wound on the side the horse.

Helping a community and the animals in it has been extremely rewarding. Members of Balzepote were so gracious about hosting us. We were treated to home-made tamales for lunch, and everyone was so patient and kind. As an added bonus, we were able to visit the beach and go swimming in the ocean to celebrate our first day working as a team, and a job well done. It is very inspiring to see what can be accomplished by coming together – I so look forward to learning and experiencing more on this trip.

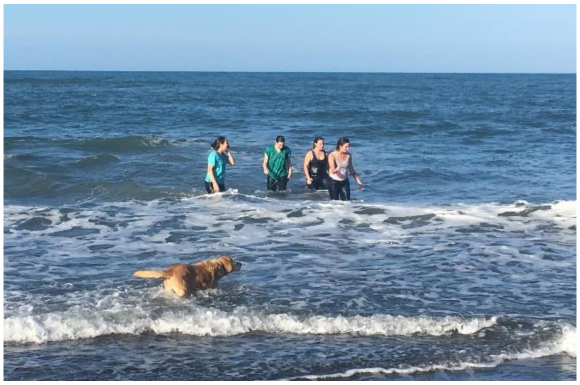
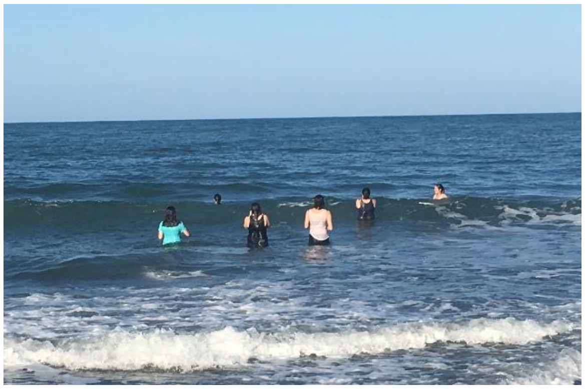
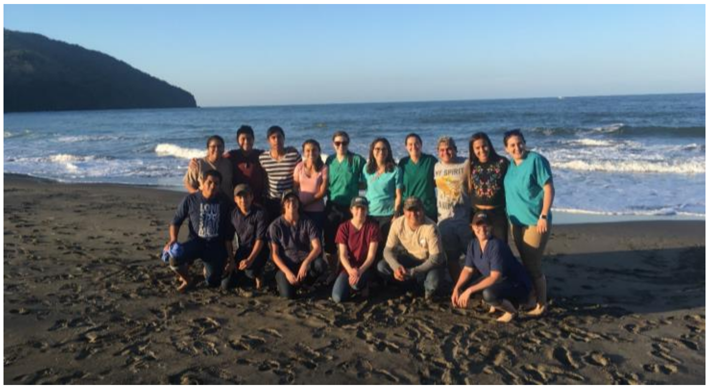

A well-deserved swim in the Atlantic Ocean at the end of our first day of community work!

Day 5 - Wednesday 1/23/19, by Skylar Thompson & Jorge Gonzales

Today started with a morning rounds session after breakfast at the Biological Station on evaluation of the cardiovascular system by Dr. Schott. After a fairly short drive through the rainforest to **La Palma**, we set up station in their soccer field surrounded by walnut trees that ended up providing useful shelter from a downpour. The community is home to about 900 people and we treated nearly 70 equids. The majority of the town's equids are horses, with a few donkeys and even fewer mules scattered amongst the population. The number of all three equids types has declined over the past 5 years, but we were still busy from the moment we arrived to the time we finished our day with a last minute castration and dental. Although we started the day with the sun shining, the last two procedures occurred in the rain, and we held a tarp over the surgery station to help prevent raining into the surgical field and to limit the amount of muddy ground.

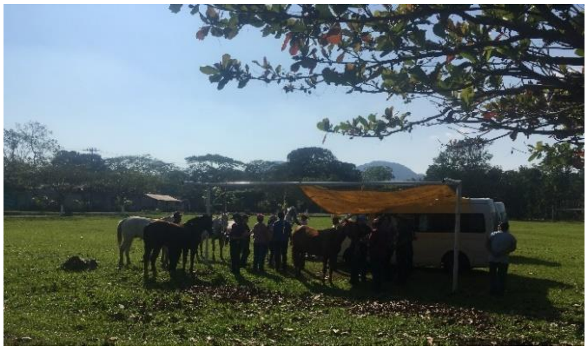
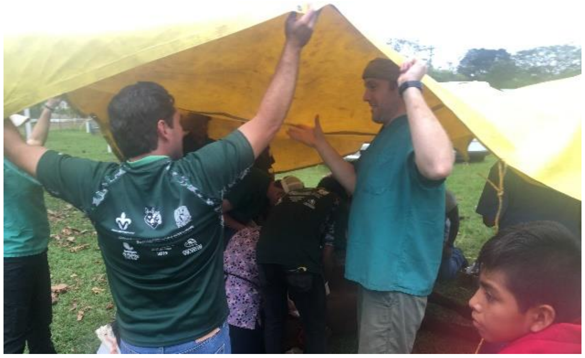

Our yellow tarp was an essential piece of equipment as it provided both shelter from the sun when we set up our supply station each morning (left) as well as shelter from the almost daily showers in the rainforest communities when castrations were performed (right)

Equids are expensive for community members to keep, and some have run away. The community leader suspects both factors have contributed to the recent decline in numbers. La Palma's equids support its heavily agricultural economy. One woman told us that it is easy to see how it's possible to live off the land within the community because so much grows well in the area. Horses within La Palma transport both people and agricultural goods, like milk, hauling up to 80% of their body weight, with donkeys

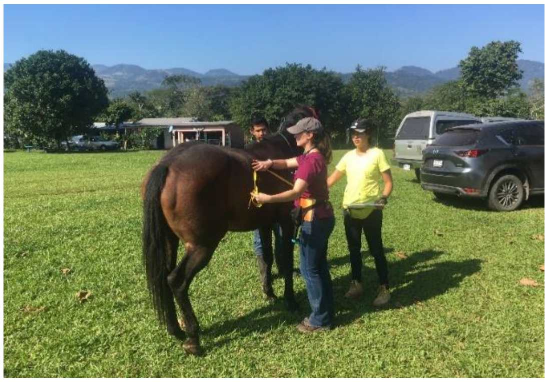
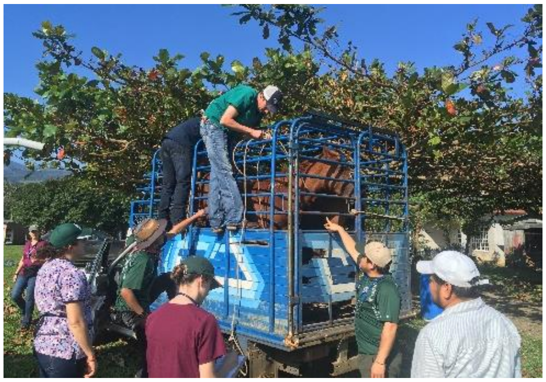

Many of the horses are reasonably well behaved, allowing us to take girth circumference and body length measurements to estimate body weight; however, others – notably mules and donkeys and young equids are not well broke requirement treatment in the trailer or after being roped and "choked down".

and mules usually able to carry a greater percentage of their body weight than horses. Additionally, the equids are used to plow the fields growing the community's crops.

Nearly all the equids in La Palma were born within the community, and we saw many foals and yearlings today. It was a new experience to watch foals get roped for the first time. For the young animals and even many of the older ones, this was their first experience with veterinary procedures. Community members would catch the foals, and once it was on the ground a community member would restrain while a veterinary students dewormed, vaccinated and administered oral vitamins. These young horses will generally start work at 2.5 years of age, working in the fields from June to December, and hauling milk year round. Their work is 7 days a week for approximately 3 hours per day. The average, healthy horse continues its work in the community for 10 years before being turned loose. It was surprising to me to see how, despite the lack of consistent veterinary care, many of the horses have decent teeth and hoofs. They are fed only pasture and allowed to graze often, and many wear down their feet appropriately during their daily work. Still, we saw quite a few overgrown hooves and mouths with sharp points that could lead to discomfort, and by the end of the day we had completed over 10 dentals, and I got to pull my first shoe doing one of the many hoof trims.

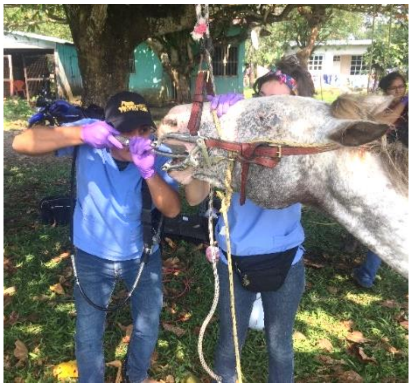
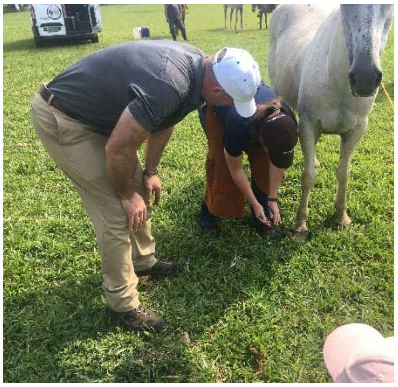
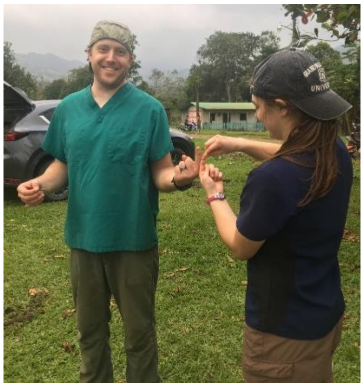

In addition to providing needed care to working equids, the Equine Welfare in Practice clerkship is all about practice, practice, practice – repetition of routine procedures by veterinary students leads to mastery and confidence. Rachel is taking her turning restraining while Hector (UV student) performs a dental float (left); Dr. Davis is guiding Katelynn through a shoe removal (center); and Dr. Kinsley offers his finger for Skylar to practice a Miller's suture knot (used around the cord during a castration, right).

Because La Palma sees veterinarians so rarely, owners have to care for their own horse whenever a problem arises. The community leader said that horse owners are commonly forced to deal with lameness that prevents work, hoof issues, gastrointestinal problems, injuries and insects, ticks or bats. The horses do occasionally receive ivermectin to help with internal and external parasites. La Palma's farrier participated in the activities today, interacting with the farrier station and occasionally jumping in on a hoof. He will shoe horses throughout the year, and those with shoes get them replaced about every month. However, there is no saddle maker or dentist in the community. One really interesting case we saw today was a younger horse that walked in with a stiff, stilted gait. The horse's mouth was difficult to open and when Dr. Alex lifted it's head, the third eyelid became very obvious – tetanus was the most likely diagnosis and

hopefully the horse will fully recover in a few weeks as it was still able to eat and drink adequately. Interestingly, the local treatment includes administration of penicillin and crocodile oil? Visiting La Palma was a rewarding experience, and I am excited to continue learning and developing skills while working in future communities.

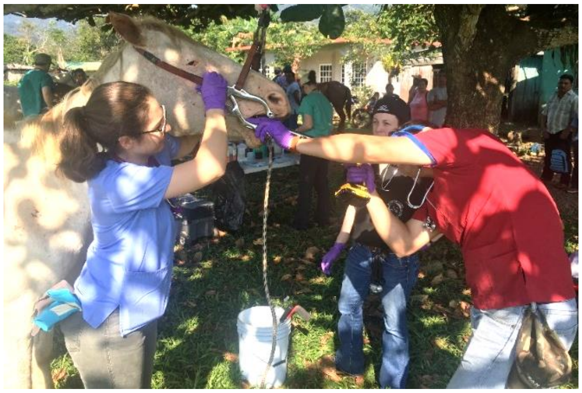

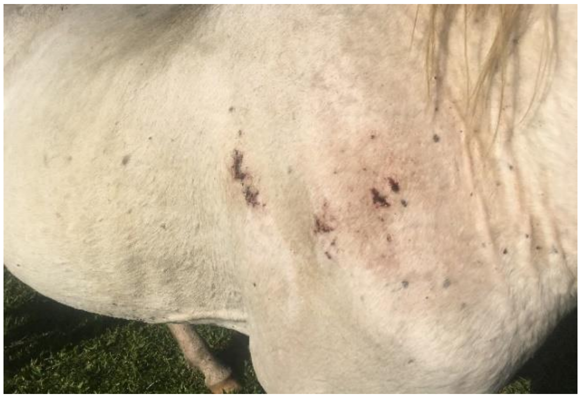

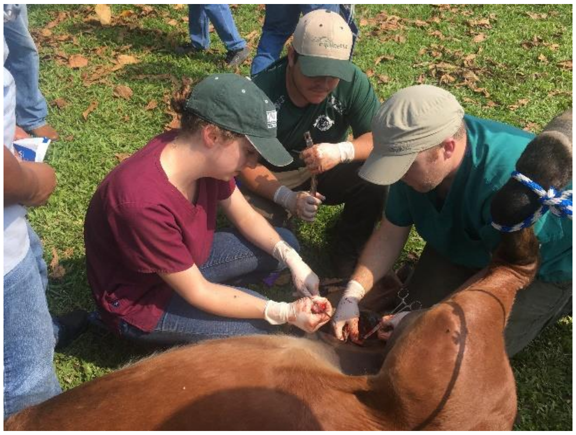

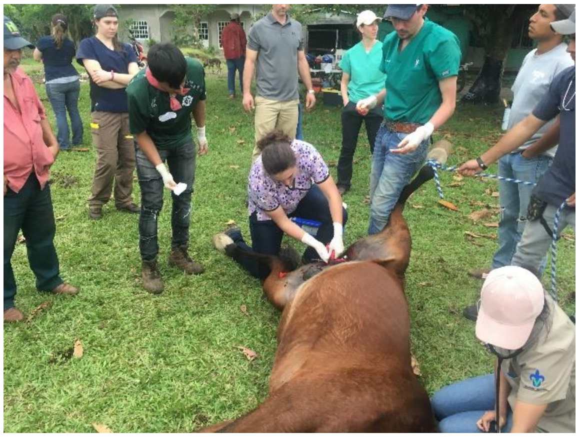

More practice: Roya is holding a horse while Geraldo (UV student) performs dentistry (upper left); on closer examination they noticed multiple vampire bat wounds on the shoulder of this horse (upper right); Kathleen performs a castration with Sam (UNAM intern) under the guidance of Dr. Kinsley (lower left); and Dr. Casillas helps Kaytie through another castration.

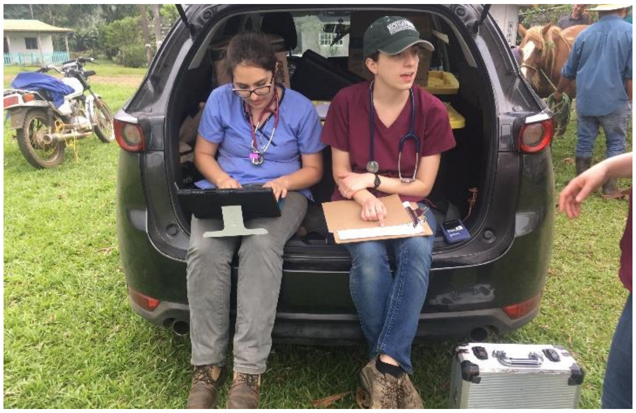

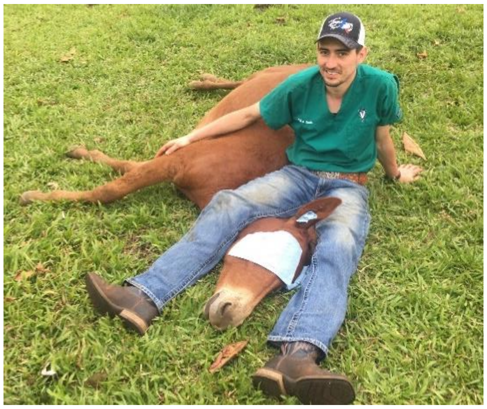

At the end of the day, record keeping is very important and Roya and Kathleen enter the day's equids into the spreadsheet (left), Dr. Casillas gets a respite while recovering a mule after castration (right).

Day 6 - Thursday 1/24/19, by Megan Ahearne & Stephanie Ortiz

This morning we ventured even further into the rainforest to the small community of **Ejido Mario Souza**. During the day my partner and I had the pleasure of interviewing the community leader Señor Rosario Chapan Teodoro. He informed us that his community is home to around 800 humans and 80 equids. We set up on their soccer field and began our day efficiently as we were becoming a well-organized group. One of the most striking aspects of the community was the interest of their younger members. We were conveniently located by their primary and secondary schools so the kids often ventured out to see what we were doing between lessons. This resulted in a rousing pick-up match of soccer, aka football, with all ages participating - community kids, doctors, our van drivers, Spanish-speaking and English-speaking students alike! We might not all have spoken the same language, but we all knew the thrill of the game and competition was fierce. We were also served a delicious lunch delivered by the local cattlemen's association (ganaderia), consisting of freshly smoked chicken, or 'pollo', rice, pasta, tamales and Coca-cola. It was a fabulous time to bond with the community.

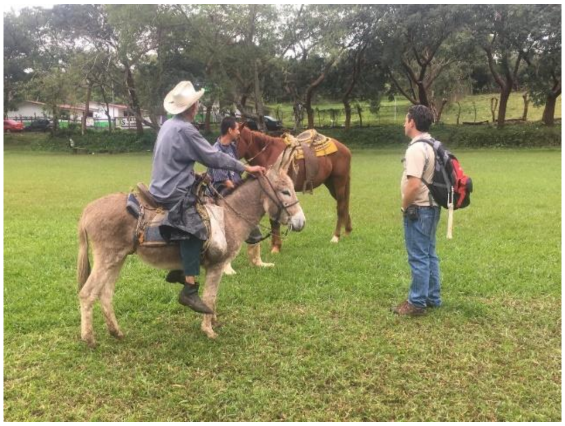
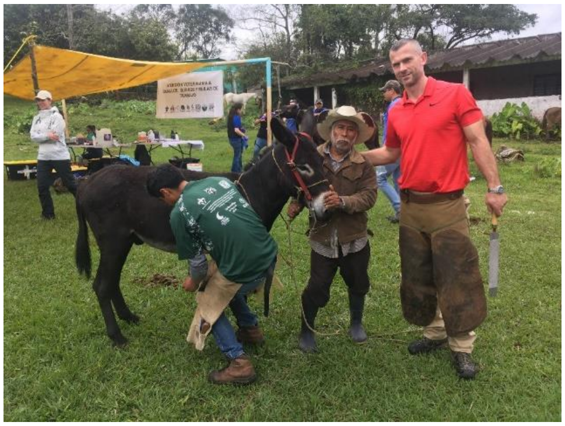

Our Universidad Veracruzana leader Dr. Alejandro greeting a community member that rode in on his burro (left) and Dr. Matt working with Luis trimming feet of another working equid (right).

Now, more about the reason we are here - the equids. Mostly horses populate Ejido Mario Souza, followed by donkeys and then mules. Due to increasing availability of motor transport and passable roads, the number of animals has been decreasing. Where equids have mainly been used to pull carts and transport goods in the past, they are more and more often kept for human transport and competition/pleasure. One of the most surprising facts that we learned during our interview was the cost of the animals. Mules cost over three times as much as horses and four times as much as donkeys. This has to do with the relative strength of mules to carry greater loads as compared to their equine counterparts, as well as the ease at which they are kept. Most equids in Mario Souza are kept solely out on the green pastures around the rainforest. They put in a hard day's work every day, working with their owners for up to 8 hours a day.

During our visit we treated almost 30 animals for everything from routine vaccines and deworming to farrier issues to dental problems. This was the first time that Señor Rosario could remember equine veterinarians visiting the community. Most veterinary

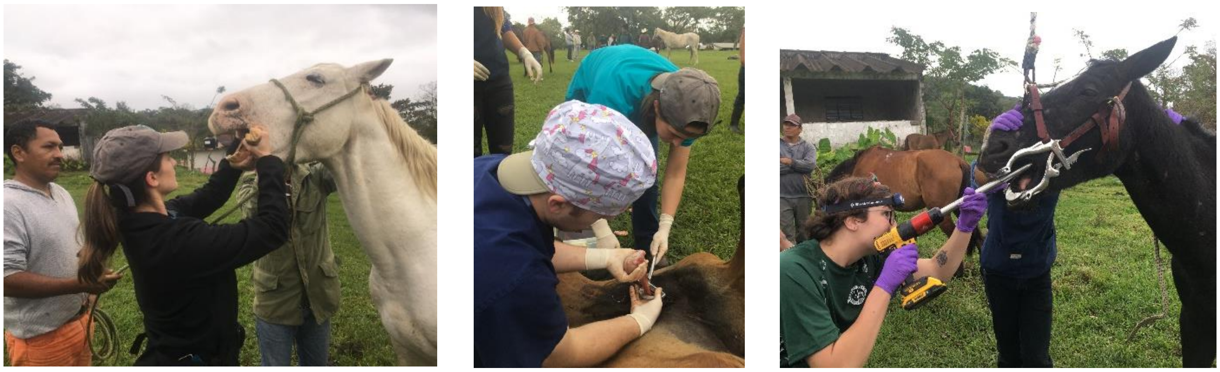

After the initial few days confidence is growing: Skylar quickly grabs a tongue and examines a horse’s mouth (left); Megan emasculates a testicle under the guidance of Dr. Kinsley, wearing a good luck unicorn surgical hat (provided by Megan) (center); and Rachel exudes confidence with the power float (right).

care is provided by the owners, with many problems going untreated. The major problems reported by Señor Rosario were hoof ailments and lameness. This region also sees a good amount of respiratory illness and wounds due to the unique rainforest climate. Thanks in part to their natural forage diet most animals had minimal dental issues with many being in good body condition despite never having their teeth floated or receiving supplemental feed. Aside from a yearly visit from a saddle tradesmen, the rest of horse care and management is provided solely within the community. Animals often serve their owners for up to 20+ years before being retired. After spending time in the community and getting to know some of the owners it was obvious that they have a deep respect for their equine partners.

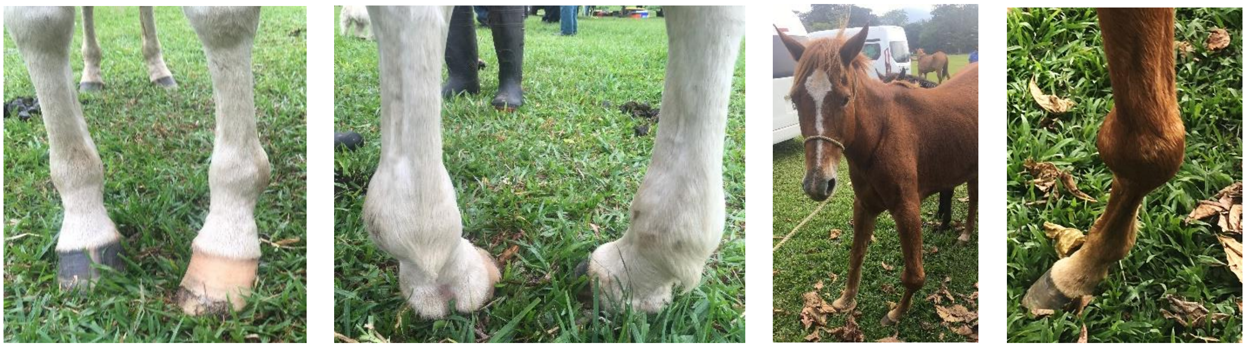

Musculoskeletal injuries unfortunately do not get the same level of care in rural communities as in the hospital settings our group was used to: today we saw a gray mare with an old fetlock injury that likely involved rupture of the lateral collateral ligament and resulted in about a 30º medial angulation of the phalanges (left) and yesterday in La Palma we saw an 8-year-old mare that sustained a carpal fracture as a yearling leading to an obvious carpal varus with minimal range of motion in the carpus (right); both mares could not work in the field and were now being used as broodmares. Our group did our best to trim the hooves of both of these mares.

It was a treat to get to visit this remote community and speak with Señor Rosario. At the conclusion of our interview he expressed his deep gratitude for all we had done for the community and hoped to see us back in the future. At the end of the day, another soccer ball was inflated and the students engaged the kids in the community for a pick-up soccer match.

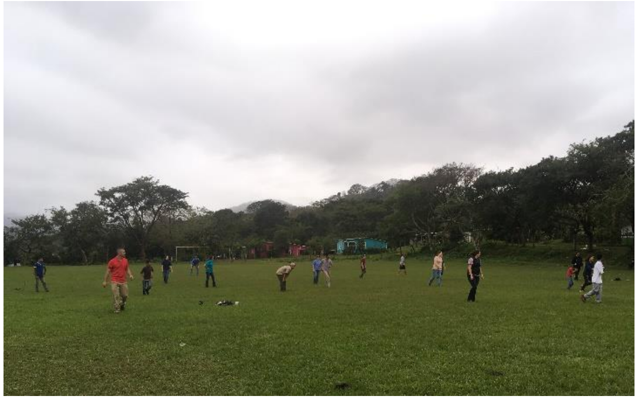

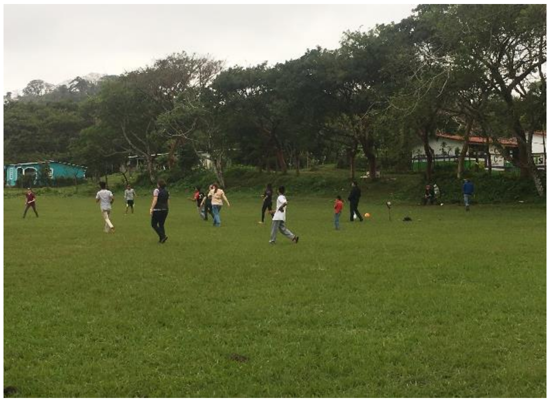

At the end of the day it was time to inflate another soccer ball and have a pick-up game with the children in the community (who were much better than us).

Day 7 - Friday 1/25/19, by Anne Meyers & Yajaira García Jácome

Today we visited the community of **Perla de San Martín**. After another morning rounds session after breakfast from Drs. Kinsley and Davis on potential complications after castration (Dr. Casillas was once again our translator), we had a long and bumpy drive up into a volcanic region. We had to use different transportation options including the back of a truck to make it up the steep dirt roads. I am susceptible to motion sickness which has been a challenge on this trip and particularly on this car ride. We worked in a soccer field that was surrounded by beautiful mountains. There was a line-up of horses waiting for us to work on despite the rainy weather when we arrived. The rain intensified as we started working and so we had to start having people record data from the vans, as our papers were getting soaked, and hold off on performing surgeries. I was cold until I started trimming feet with Dr. Casillas which instantly warms you up and gives you a good leg and back work out. As the day went on, the rain slowed down and the sun came back out.

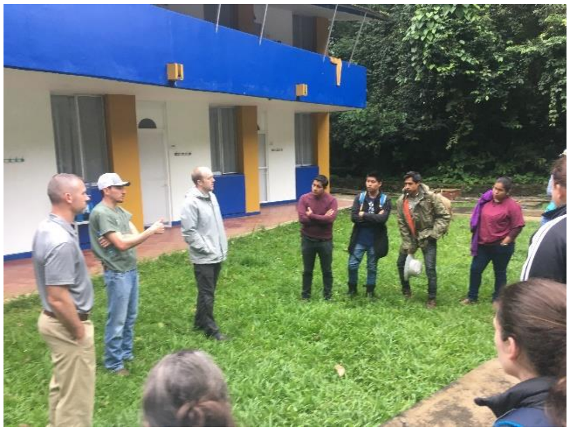

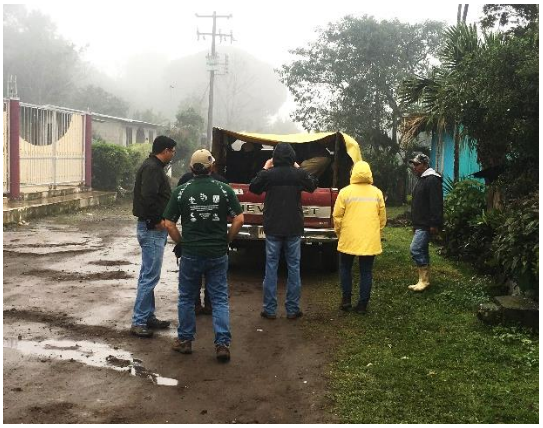

Morning rounds with Drs. Kinsley and Davis after breakfast at the Biological Station (left) and loading up into a truck for the trek up the bumpy roads to Perla de San Martin (right).

I spoke with one of the leaders in the community, Guillermo Ramirez. He estimated the population to be 450 people. The people who came to us with horses were from a larger area than just the immediate community as the population is more spread out in this

area. The economy in this area relies on agriculture. They grow mostly corn and beans. The uses of equids in this community are to transport agricultural goods, transport people, and herd cattle. The population of equids is about 200 with 80% being horses, 10% donkeys, and 10% mules. The numbers of all equids in the community has declined as the use of motorcycles has become more popular. Equids in the community are bred and raised in the community with little buying and selling from outside areas. The equids in the community live on pasture forage. Horses, donkeys, and mules all begin working around 3 years of age and work all year round. When they work, it is for 3 hours a day. They work 5 days a week. The average horse works until they are 20 if they stay healthy, and donkeys and mules work until they are 25 if they stay healthy. When equids are no longer used they are sold when possible.

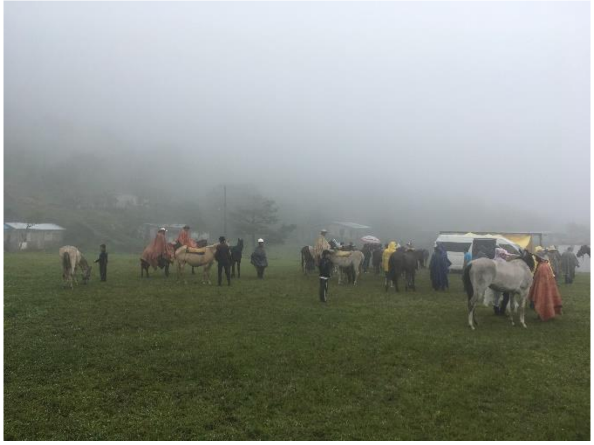
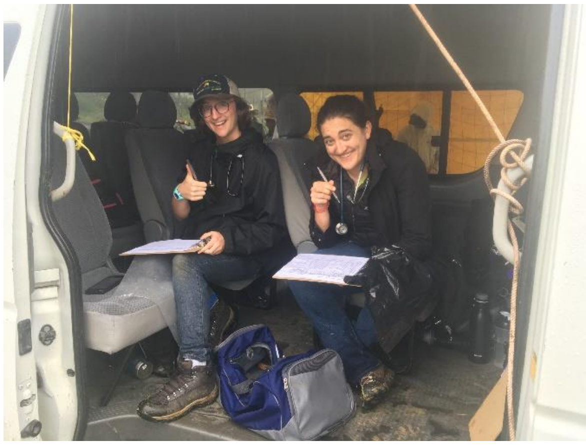
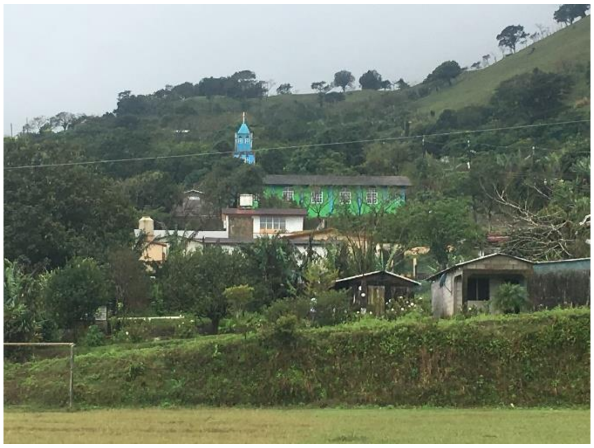
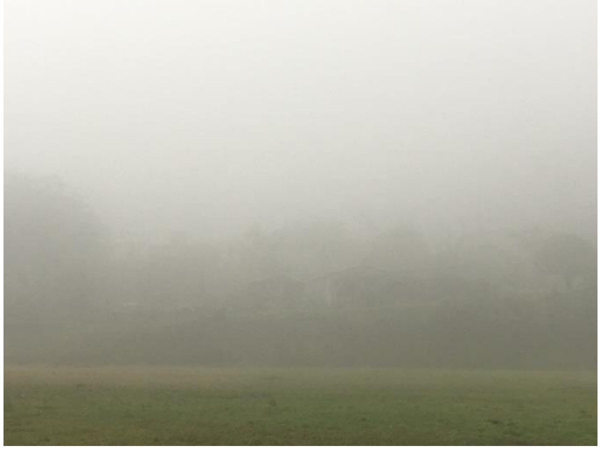

It was a misty and rainy day at Perla de San Martín prompting Rachel and Kaytie to retreat into one of the vans to record equid data without getting the papers too wet! A colorful church appeared and disappeared throughout the day as the mist came and went.

The last time a veterinarian came to the community was 1 year ago so much of the health care performed is performed by the owners. The most common medical problem was intestinal issues including colic and diarrhea. They have problems with lameness, decreased fertility, and intestinal parasites as well. The horses are treated by some owners with ivermectin monthly and Bovitrax every month. No other preventative care is provided. There is a farrier that comes to the community every 3 months. The farrier

visits do not always result in healthy feet for the equids in the community. We trimmed many feet with shoes on that were too small for the feet or the feet were not being trimmed frequently enough. The community leader estimated that 60% of horses were shod, 5% of donkeys were shod, and 90% of mules were shod. He believes shoes are replaced every 3 months when the farrier comes. There has never been a horse dentist or saddle maker/fitter in the community so our presence was appreciated.

Many children from the community came to watch what we were doing and talk with us. Bathroom facilities are always an interesting situation for us and in this community one of the houses nearby allowed us to use their bathroom. This was my first experience seeing the inside of a house in a community. It was simple but functional and the children were very happy to talk to me and show me their toys. I have not taken Spanish since middle school as I took French in high school so I was not very fun to talk to for them. I definitely wish I stuck with Spanish into high school as it is very useful in the veterinary field. There has been very nice scenery on this trip but this community stood out for me as one of the most beautiful especially once it stopped raining.

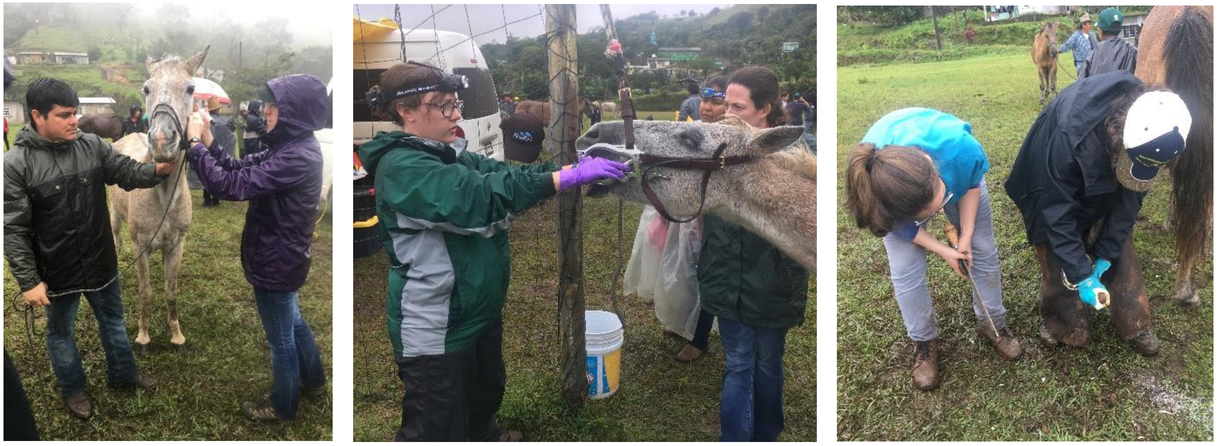

Despite the rain, work continued with Kathleen and Sam treating a horse (left), Kathleen examining a horse for dentistry with Dr. Lauren Fischer (center), and Rachel and Roya trimming feet (right).

Later in the afternoon we saw an interesting case of a horse with cranial nuchal bursitis, also known as poll evil. Although this lesion used to be associated with brucellosis in horses, a more likely cause was a traumatic injury. The swelling was a rather large lesion but did not appear to be significantly affecting the quality of life of the horse. The owner said the swelling had been there for 2 years and that the horse was able to work without any difficulty. We learned that a treatment for this in their community was to slice it open with a hot machete, which we definitely did not want to do. Unfortunately, nuchal bursitis is difficult to treat even in large veterinary hospitals like MSU, so we left the problem alone.

Visiting Perla de San Juan was tough on the team physically because of the rain and mud. We worked through it and were able to take care of many working equids. We enjoyed hot cups of a cinnamon coffee throughout the day provided by the community.

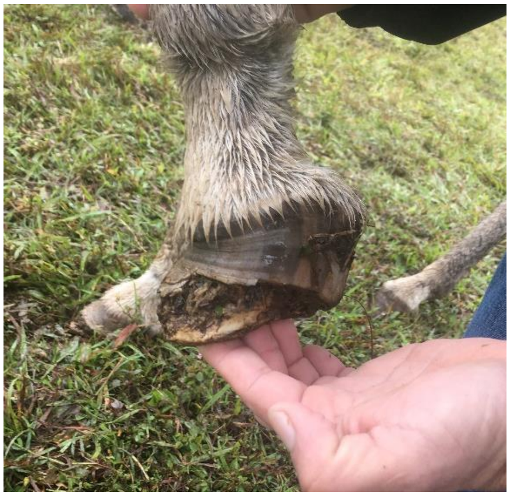
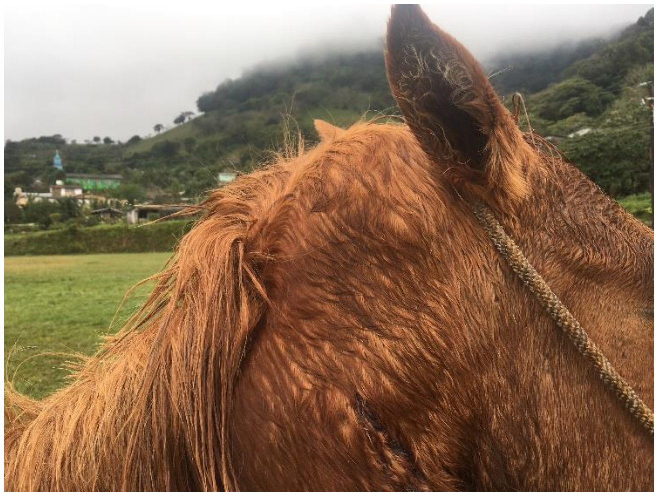

Today we saw a burro that had avulsed a large portion of the hoof capsule (left); unfortunately, the burro will likely be lame for several months until the foot can regrow. We also saw a horse with nuchal bursitis – the bursa had been enlarged for over 2 years and had substantial mineralization; however, the horse was not painful and was still able to perform work. Thus, we left the injury alone at this time.

The trek back home was less rainy and I enjoyed a warm shower when I got back to the biological station and prepared myself for another day of work.

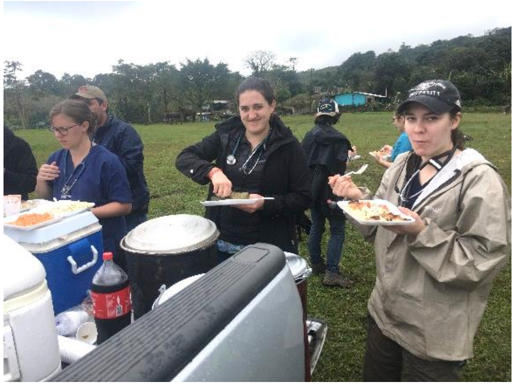
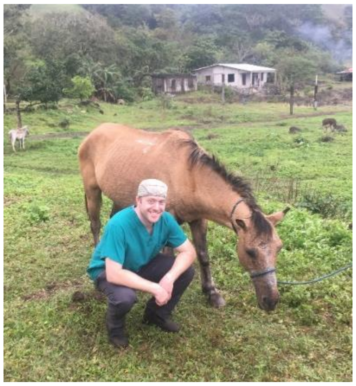
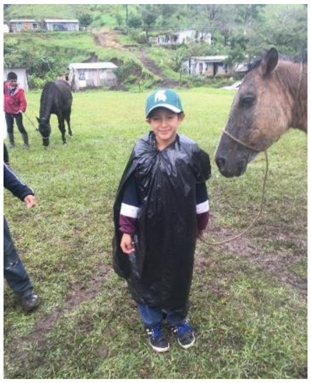
A much appreciated hot lunch and hot cinnamon coffee were provided by the Cattleman's Association (left): Dr. Kinsley met a horse named "Cacahuate" = peanut (center, fortunately, he was not allergic to this equid); and a community boy learned to shout "Go Green" and earned himself a Spartan hat.

Day 8 - Saturday 1/26/19, by Kathleen Grace & Samuel Aponte Landeros

Today we visited our fifth community, **Rancho San Joaquín Las Margaritas-Tebanca** after a morning rounds session with Dr. Esser on examination of the respiratory system. Though the drive to each community so far has been beautiful, the drive to Rancho San Joaquin was exceptional in that we had a view of a huge inland lake (Lake Catemaco), an area popular for tourism. We could see women from the surrounding villages in buildings along the road who were weaving baskets from reeds that grow on the side of the lake. Today's site was also different in that it was not an open field. Rather, we drove up a drive of cobblestones surrounded by pastures to a small building. We already had a crowd of horses waiting for us - we had to drive through a herd of them

which startled a few of the foals. The cobblestone triage area was a welcome change from the soccer field that turned into mud in our previous community. Our surgeons were probably most pleased about this. The crowd of individuals at our site added to the “ranch” aesthetic as many were wearing straw sombreros, or cowboy hats. This included even some of the youngest cowboys I have ever seen. Brahman cattle supervised our triages, farrier work, and castrations with minimal interest.

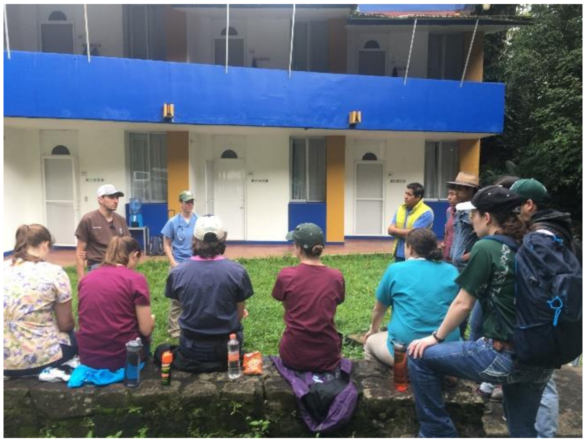
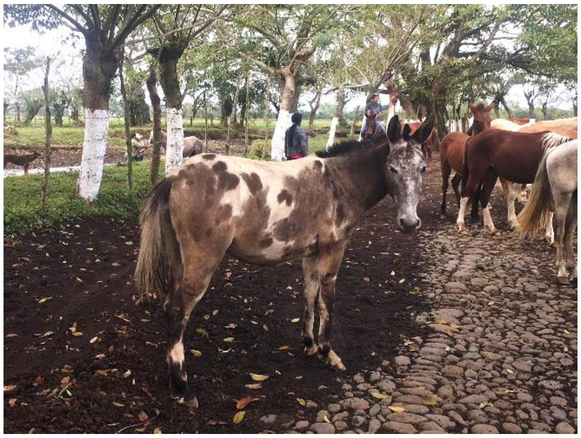

The morning started with rounds after breakfast with Dr. Esser (left) and then we loaded up and arrived at Rancho San Joaquín Las Margaritas-Tebanca where horses were lined up waiting for us (right).

Today I spent some time at the dental station with Dr. Lauren Fischer. She was very pleased that this new community provided a makeshift dental suite in the form of a back patio that was sheltered from the rain and had enough room to simultaneously run two dentals. Dr. Lauren tested us today by allowing us to act as doctors and be in charge of a full dental by ourselves. This included sedating our horse, setting up the oral speculum, performing external and internal oral exams, and using the power float to correct any issues. This was a valuable exercise for us as it is important the closer we get to graduation to remember that we will be in charge of our own procedures soon enough. I am already becoming more confident in my dental abilities thanks to Dr. Lauren.

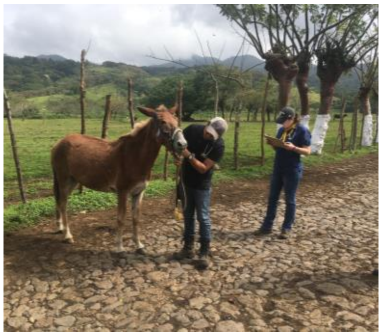
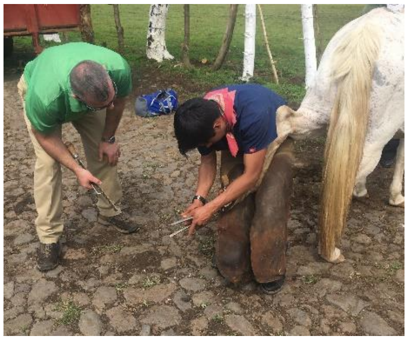
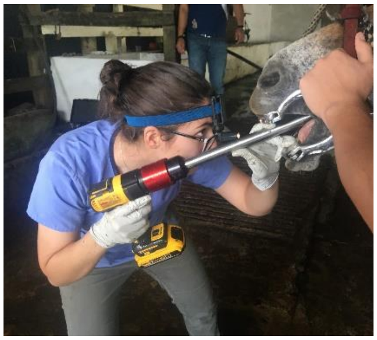

The days always started with admissions/examinations – Jorge is examining the mouth of a horse while Skylar is recording data (left); Dr. Matt is guiding Luis through a foot trim (center); and Roya is working away with the power float (right).

Sadly, we had to say goodbye to Dr. Matt Davis today as he headed back to Veracruz for his flight home. We all appreciated his teaching, patience, and enthusiasm during his time with us. We were amused watching the young Mexican boys blush when he shook their hands like they were grown men and laugh as they played soccer with him.

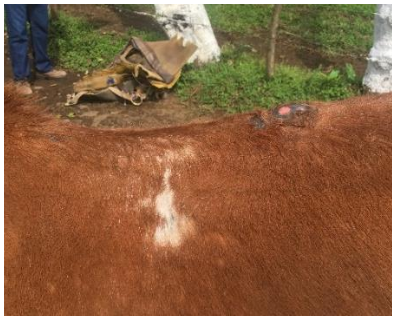
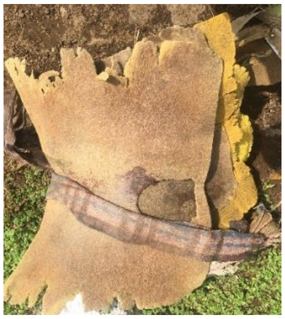
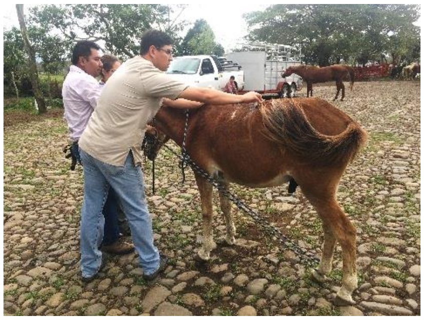

Saddle sores (left) and other harness trauma wounds are an ongoing problem for many horses That work every day. Examining saddle and padding fit (center) and making adjustments, along with cleaning the wounds (by Geraldo, right) was another expertise of Mauro from the Donkey Sanctuary and he helped many equid owners to improve saddle fit.

An unusual situation for rural equine services presented itself to us today when we had a horse arrive with prior bloodwork. A human physician had performed bloodwork for his daughter’s horse that had been losing weight despite receiving an adequate diet. The owner was concerned the horse may be anemic, but the bloodwork revealed a low total protein concentration. We checked the sclera for a color change which may indicate liver disease and for any signs of edema. Based on physical exam, we decided the most likely reason for weight loss and low protein was a protein losing enteropathy. We performed transrectal palpation in order to confirm there was no palpable mass in the abdomen that could support neoplasia. Ultimately, we sent the owner home with instructions to try a tapering dose of dexamethasone for possible inflammatory bowel disease. This was a nice exercise as it allowed us to see how we can best help with what resources are available in the field without the aid of advanced diagnostics.

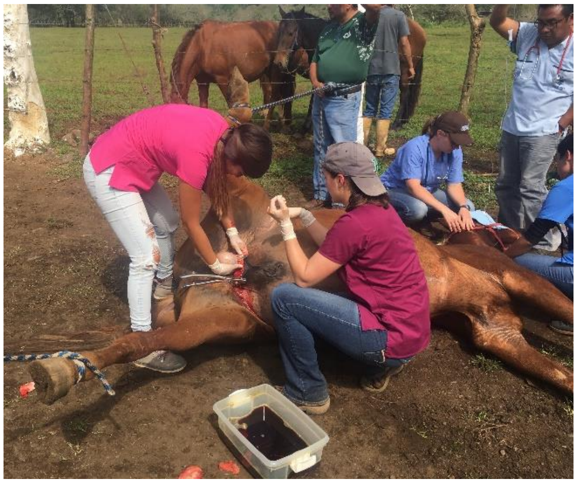
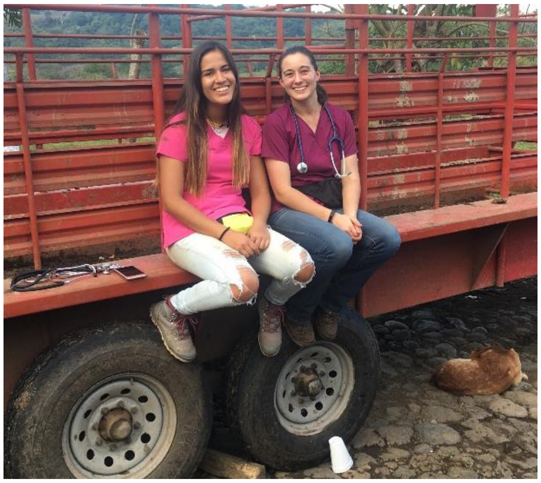

One of the fun part of this experience is that students work in teams of US and Mexican students: Megan and Stephanie are castrating a horse (left) and later in the day get a chance to rest a bit watching their classmates do some farrier work (right).

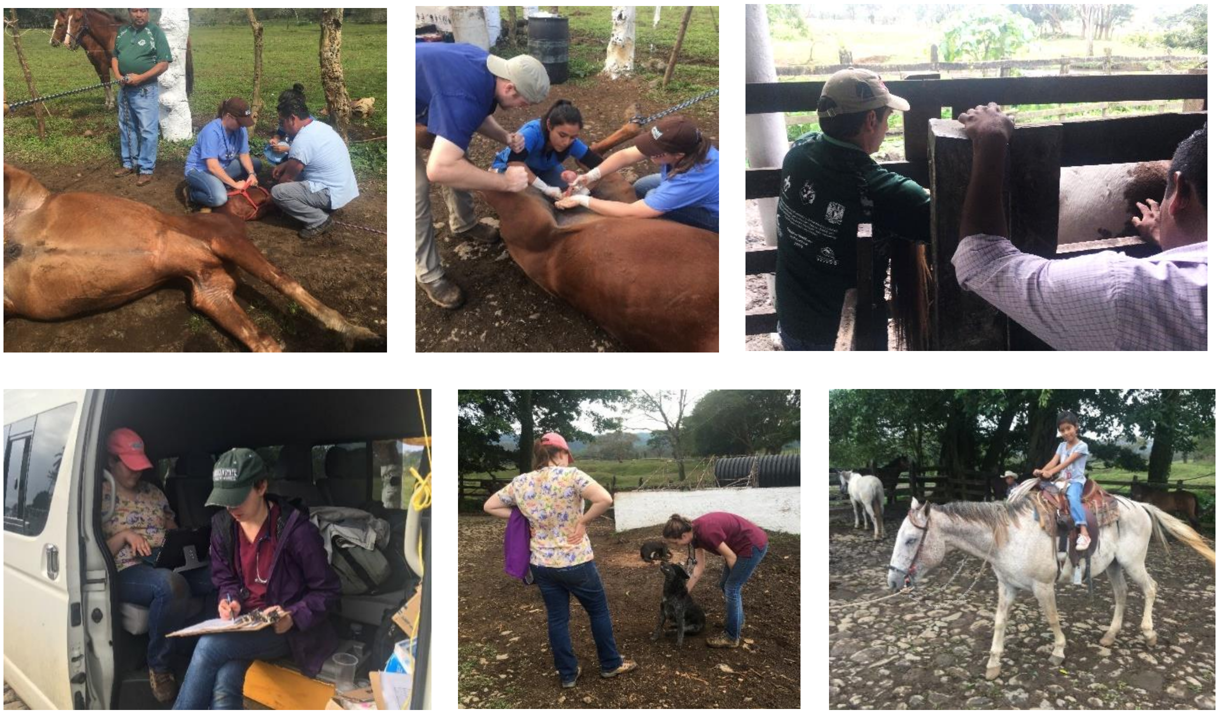

Before field surgeries can be successfully performed, good anesthesia is paramount and Katelynn and Mariela perform anesthesia with Dr. Antonio (El General) (upper left) before moving on to be the surgeons on the next horse to be castrated (upper center). All the while we are working at other stations, Dr. Alex has been palpating mares with many students to assess pregnancy status (upper left) and some mares are not that easy to palpate! At the end of the day, record keeping is again critical and Kaytie and Kathleen work together to complete the excel spreadsheet for the day (lower left). After that they find some time to play with a couple of the local canine friends and also say hello to a young girl riding her horse – a good reminder about why we are here to help both working equids and their owners.

We wrapped up our day watching the clouds retreat up the mountains and petting the sweet farm dogs. When interviewing the community leaders, they were quick to ask me if we would be returning again next year. Considering the great experience we had today, I certainly do not think we would hesitate if given the opportunity to return to Rancho San Joaquin.

Day 9 - Sunday 1/27/19, by Roya Oliai and Gerardo de Jesús Rivas Gómez

On Sunday we awoke to heavy rain in our Las Tuxlas rainforest dormitories and hoped that it might be dry in the community we would be heading for today. After breakfast we traveled to **Zapoapan de Cabañas**, a community on the opposite side of Lake Catemaco, across from where we had previously worked on Saturday. The day began with sun and we set up our work stations in a tall grassy field next door to the community's soccer field. Typically, we would arrive to a line of equids; however, today was an anomaly and there were no horses or burros waiting. We were informed that this morning the community held their monthly meeting and that most members would bring their equids after the meeting was over. We did not have to wait very long and Dr. Jose started the day with farrier work on a mule. A teachable moment came when a group of students ausculted the heart of a 9-year-old work horse on routine physical exam. A grade 5/6 holosytolic murmur was ausculted on the left side with a subtle thrill palpable

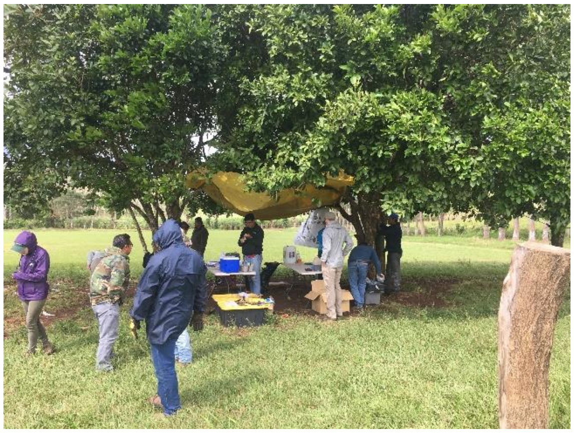
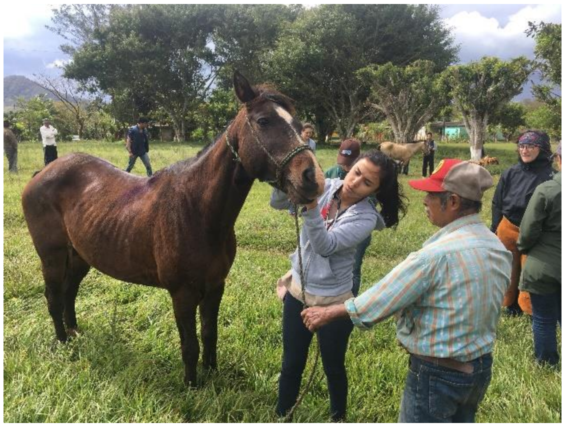

Although it was a chilly morning in Mexico, we were all glad to be far south of the snowstorms and bitter cold in Michigan that actually resulted in cancellation of MSU classes, except for students participating in international programs where the weather was fine. As usual, the day started with admitting, examining, vaccinating, and deworming horses, along with a dose of vitamins administered by Mariela, with Rachel in the farrier chaps looking on.

over the chest. Drs. Schott and Esser took the chance to question us about common causes of murmurs, locations to auscult, and better history questions to ask the owner. The horse’s resting heart rate was normal (suggesting adequate myocardial contractility and cardiac output) and when we asked about how the horse worked, we discovered that it did not have any exercise intolerance and was able to work well for his owner, good news for all. The murmur was likely associated with a leaky mitral valve that may have been a congenital or acquired problem.

As the day progressed clouds came over the hills, winds gusted, and the rain started again, much to our dismay. Overall, we examined and administered preventive care to 30 equids today, with two castrations and one sarcoid removal. The first castration was a left cryptorchid and Dr. Kinsley demonstrated the technique for a field cryptorchid surgery. We also removed a sarcoid from the inner thigh of a 2-year-old filly. Towards

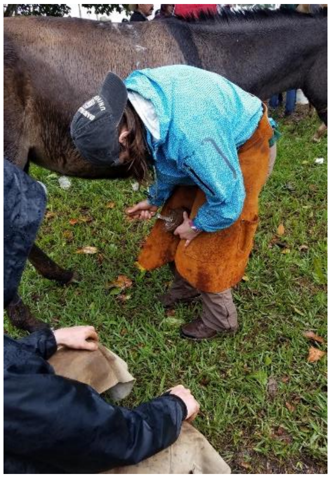
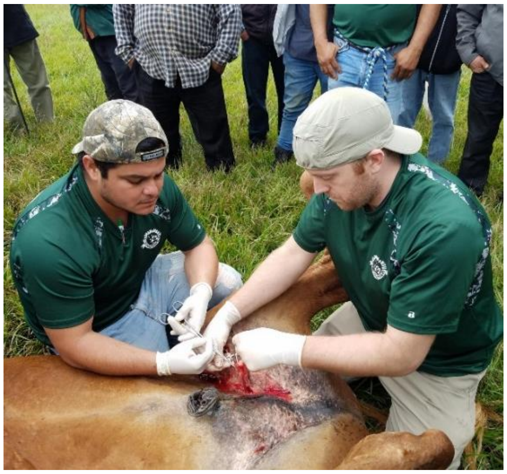
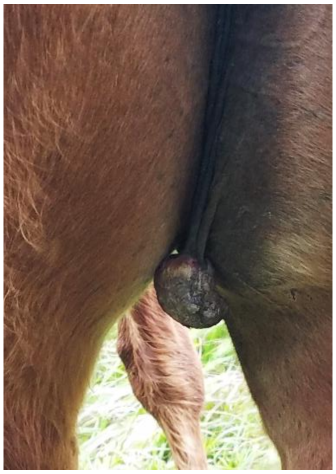

Although we saw fewer horses today, we stayed busy with the basics: Skylar trimming a foot (left); Sam performing a castration with Dr. Kinsley (center); and a sarcoid needing removal (right).

the end of the day as we began packing up the trucks we received a notice that there was an owner who wanted their 8-year-old horse castrated, he quickly brought him to our station. We unpacked the surgery supplies and proceeded with the surgery. At the end of our day we had a surprise visit from a food cart. He served up street corn and fried pork rinds with hot sauce and cheese. The prices were quite reasonable and the hot corn was well-received by students and faculty longing for something warm at the end of a windy, cold, rainy day.

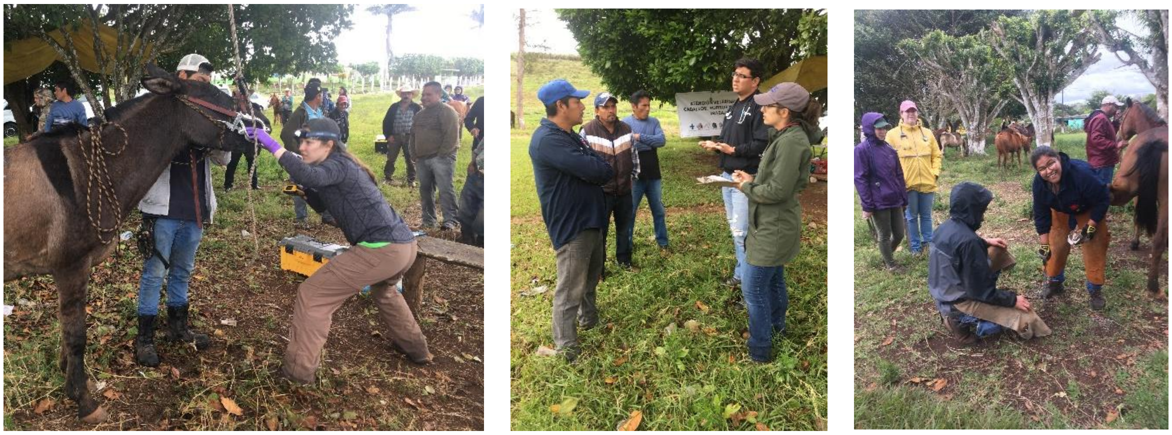

In addition to providing care to the working equids (Megan is floating teeth with Jorge assisting, left), we interviewed leaders in each rural community we visited (Rachel and Geraldo performing an interview, center) to determine how many equids were in the area and what jobs they performed. Yajaira, with Dr. Casilla's guidance, learned that one of the best ways to warm up on a chilly, rainy day was to trim feet (right).

Though we intermittently got soaked and tried to stay huddled together under our tarp canopy to stay dry and warm, we were able to provide care for many families' horses completing 9 dentals and 8 hoof trims/care. We learned that Zapoapan de Cabañas is a community of approximately 2,400 people. Their main economy comes from farming corn, rice, beans and peanuts. Within the community they estimate that there are 50 equids. With about 40% being horses, 40% donkeys and 10% mules. The community leaders reported that equid numbers had decreased over the last 5 years due to increased use of motorcycles. The equids they do have are used for herding cattle and transporting products such as milk. The community leaders also told us that there was no difference in use between the types of equids used (horses, burros, or mules), they are utilized equally and all carry approximately 50% of their weight in a load. It was important for us to provide veterinary care for their equids as there was no local veterinarian or person who provides medical care for the horses. There is a person who lives in the community that makes saddles and we noticed there were less saddle sores here compared to previous locations.

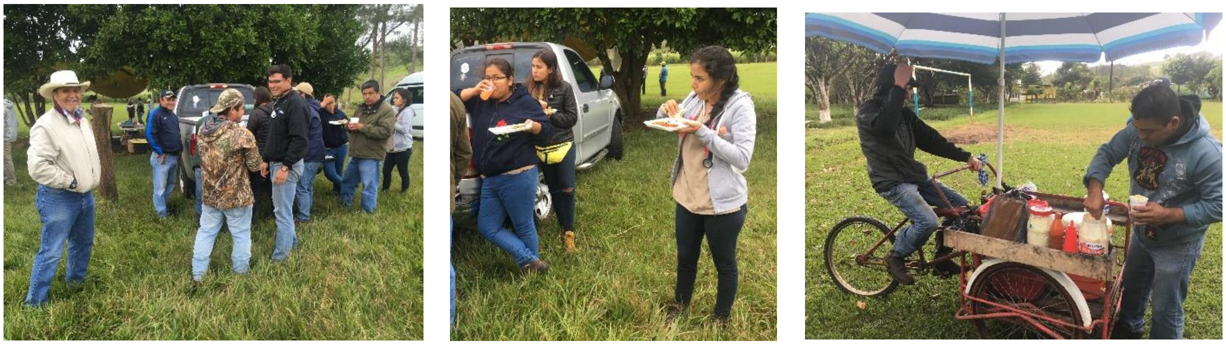

From our 2nd through 6th days of community work, we were happy when the Secretary of the Catemaco Ganaderia (Cattleman’s Association, gentleman with sombrero, left) arrived each day with a hot lunch and cold water and cervezas. At the end of this on and off again rainy and chilly day, we also warmly greeted a local food vendor that brought warm corn and other treats – Dr. Casilla’s temporarily considered driving off on the bicycle cart as a new career.

The major problems equids in this community faced were hoof problems, wounds, colic, and tetanus. The gentlemen we spoke with indicated there is no veterinarian to help if a horse develops colic. They asked for advice on how to handle colic cases and we discussed removing feed, hand-walking, and recommended picking up flunixin meglumine from their pharmacy when they next go into town. We are confident that our recommendations and care provided today helped the equids in the community, not only for today but for the future as well. As a highlight, our team and our work made the regional news channel that evening!

Day 10 - Monday 1/28/19, by Hal Schott

Today we said goodbye to Rosamond Coates, director of the UNAM Los Tuxtlas Biological Station, and her staff and headed back to Veracruz. It was a well-deserved day off from community work and the students were tired, after spending a late evening in the library dancing with their Mexican counterparts. We initially headed south to Catemaco where we refueled the vehicles and the more adventurous tried some

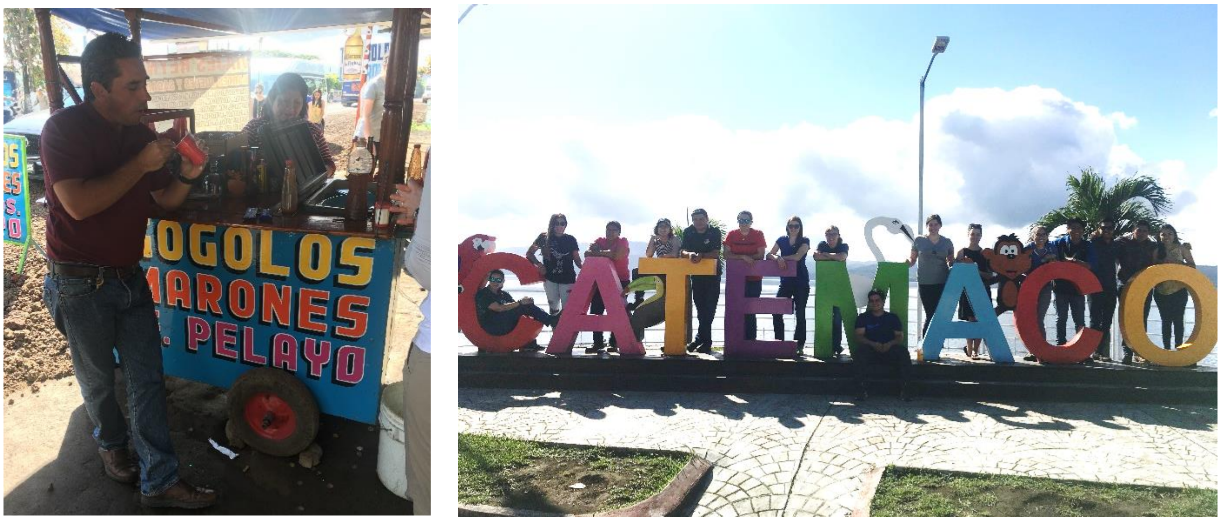

Our Universidad Veracruzana faculty leader Alex enjoying some tegogolos (left) and the students line up in front of Lake Catemaco (right), a popular tourist destination for Mexican nationals.

tegogolos, a freshwater snail indigenous to Lake Catemaco that is served raw in a tomato sauce and considered a delicacy for the region. A short while later we had a slight detour to visit El Salto de Eyipantla, the widest waterfall in Mexico. The view was certainly worth the 500 or so stairs that had to be climbed to reach the falls! Then it was back in the vans for our trip north to Veracruz. We arrived early afternoon and the students had a free afternoon to explore the Aquarium and Zocalo of Veracruz – they even found a Mexican pizza restaurant for dinner as they were starting to have a bit of a craving for something other than rice and tortillas.

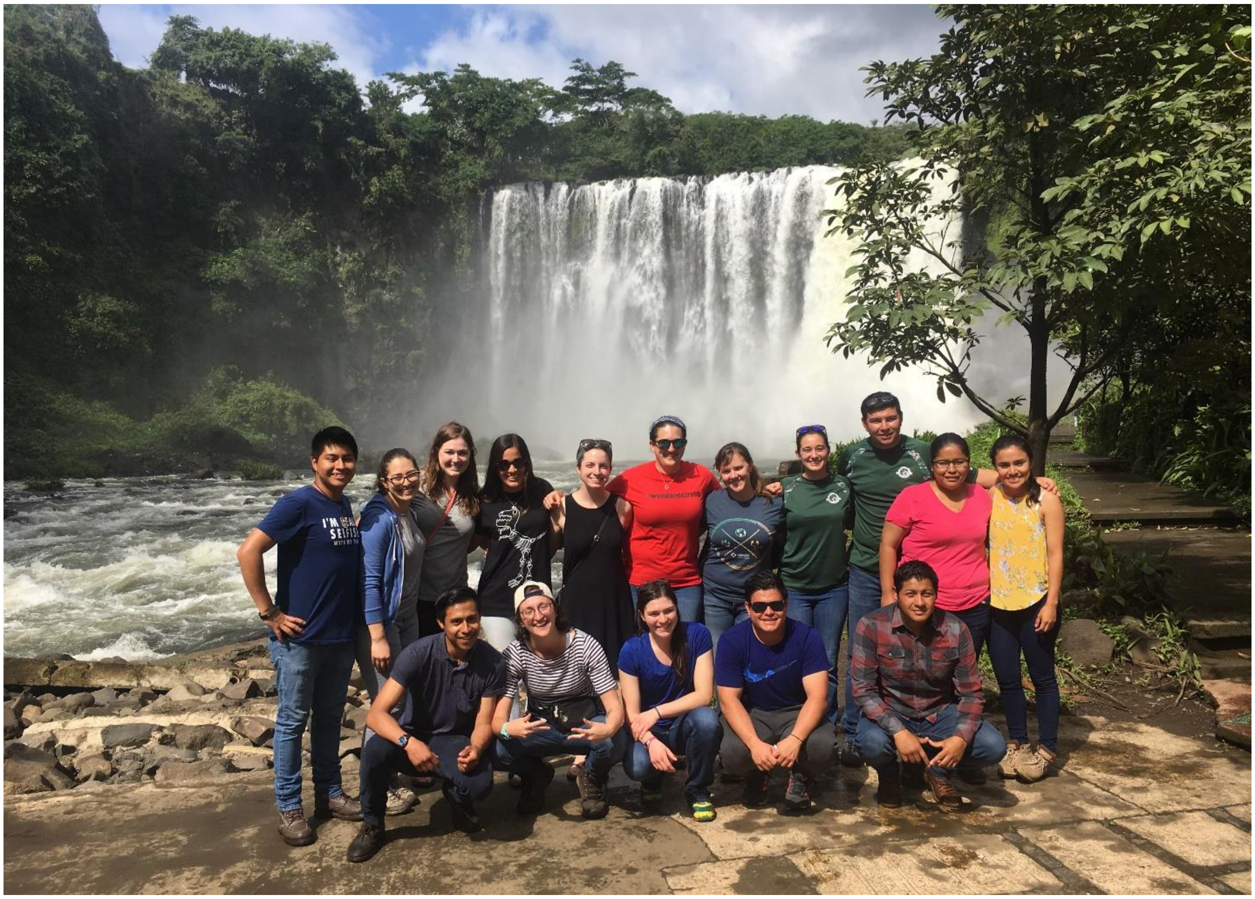

The US and Mexican students enjoy a stop at El Salto de Eyipantla, the widest waterfall in Mexico during our travels from the Los Tuxtlos Biological Station back to Veracruz.

Day 11 – Tuesday 1/29/19, by Kaytie Voirol and Luis Everardo Ponce Angeles

Today was our first day of working from our new home base out of Boca del Rio, Veracruz. We started the day off with a great breakfast from the hotel restaurant before packing up for the day and leaving by 8 am. We had about an hour drive before we arrived in **Camaron De Tejada**. This community had more donkeys which would end up being a great break-in for the days to follow. This was the seventh community that we had worked in and we felt ready to take on the higher numbers that we were expecting. We ended up seeing over 78 horses, donkeys, and mules. We completed 9 castrations, 19 hoof trims, and 17 dentistries during this long day. Among the many “typical” procedures, we also ended up working with a few unique cases during this visit.

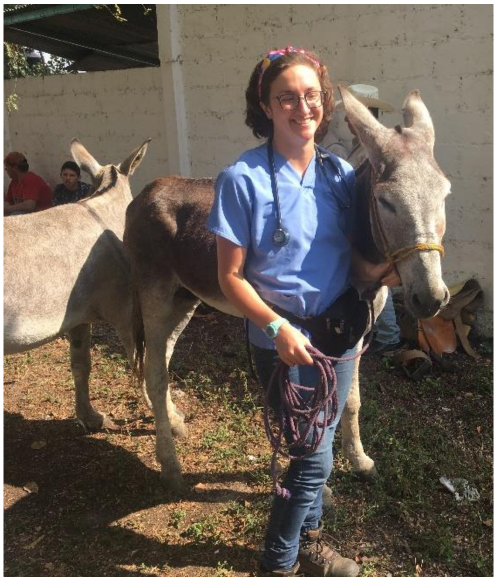
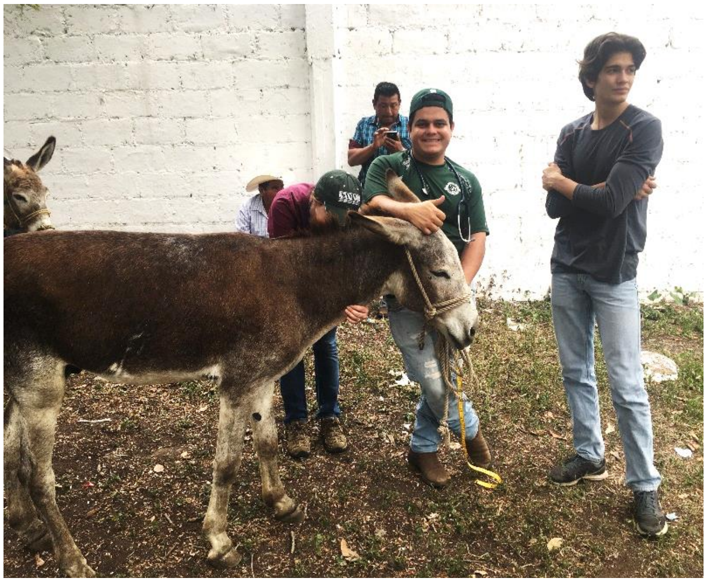

Our 7th community was Samuel's hometown where we saw more donkeys (Rachel has a new friend, left) and Sam was joined by his younger brother (right) who also wants to become a veterinarian.

Camaron de Tejada was home to one of our UNAM participants, Samuel who had graduated from at UV and was now pursuing an internship at UNAM. We had the pleasure of meeting a couple of his family members throughout the day, including his younger brother who also wants to become a veterinarian and his grandmother who kept us company all day long. It was fun to see how proud they were of the work that Sam was doing. Dr. Jeff Bunn from Lowell, MI also joined our team today bringing some new energy and enthusiasm to our Equine welfare in Practice team!

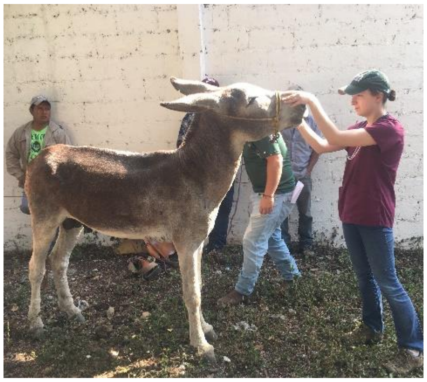
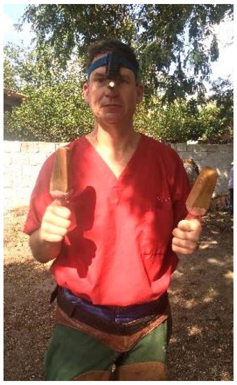
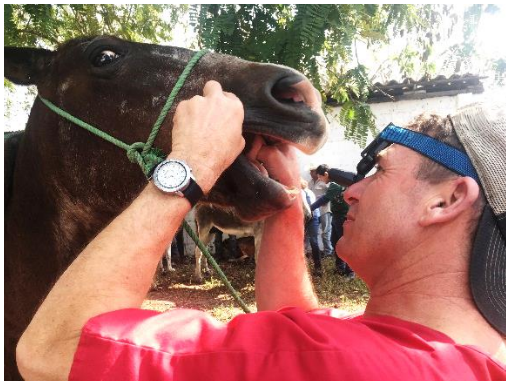

Our 7th community was Samuel's hometown of Camaron de Tejada where we saw more donkeys (Kathleen is examining a donkey's mouth, left). We were also joined by Dr. Jeff Bunn for the rest of our trip. Dr. Bunn has many entrepreneurial ideas and he brought along a couple of homemade wooden ramps (center), although the students initially were hopeful that he was offering popsicles, to place on each side of the mouth to examine the teeth (right).

We had a pretty great set up in the community's Cattleman's Association compound and pavilion, even though working space was a bit tight during the peak of the day. This was our first time not having an open field to work in, and the space constraints could be challenging when trying to get from one side of the compound to the other. This was

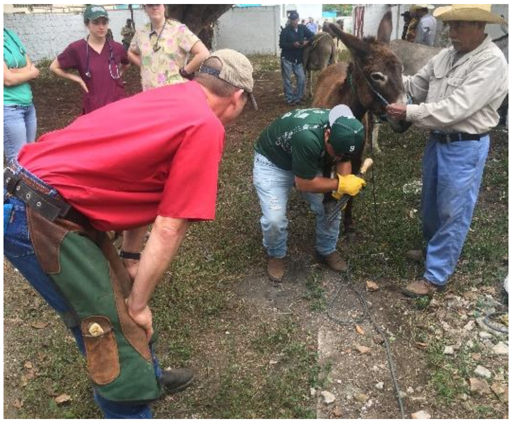 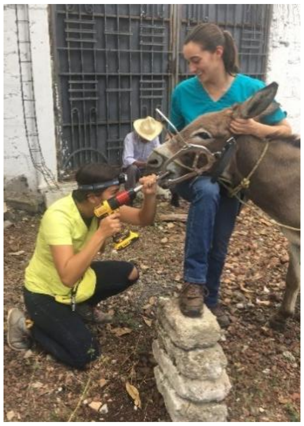 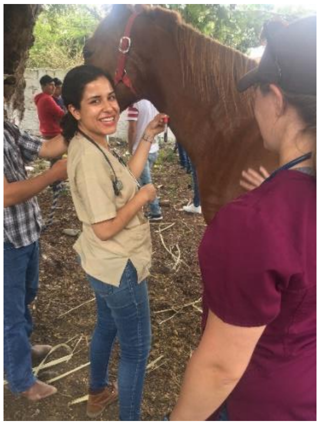

Once again, it was practice, practice, practice as Dr. Bunn helps Sam with farrier work (left), Megan holds a donkey for Stephanie to float its teeth (center), and Melissa collects blood for Alex and Hector's study of prevalence of antibodies against *Leptospira* spp. in the various communities where we are working.

the second year visiting this community and community members were very grateful for our return. Luis and I had the pleasure of interviewing Camaron's community leader, Luis Palarios Ortega. According to Sr. Ortega, the community has seen a decrease in their equid numbers over the last 5 years due to increased use of larger farm equipment and machines to do the work that the equids were needed to do, previously. Camaron de Tejada's main agriculture products are sugar cane, corn, and beans. Their population was larger than in our previous communities with 7,200 people, and about 1,200 equids. They still had a majority of horses, then donkeys, and fewer mules. The community leader informed us that most of the equines in this community were born and raised here. They do not tend to buy from outside stock. A majority of the work that these animals are doing occur during the summer months of May through November for about 3 hours a day and 5 days a week. He estimated that they work for a solid 10 years of their lives before they are retired, or pass away. Unlike most of the other communities that we visited, the community has a local veterinarian, but not many people use him - he would not state a reason why that was the case. The equids were primarily on pasture, but most were given forage, and concentrates, as well. We were also told that many of the owners give their animals ivermectin every 3 months, as well as an external anti-parasitic every 15 days. This was more care than what we were used to finding at this point in our trip.

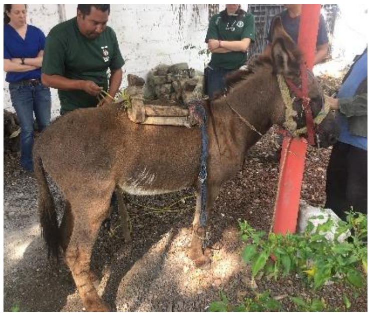 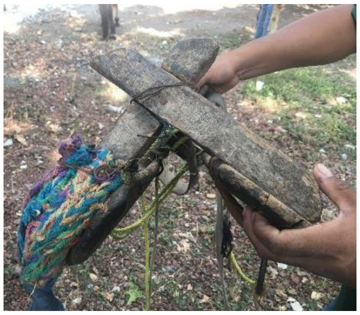 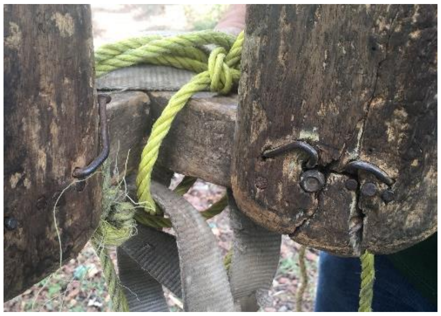

One of the challenges that community members face is having saddles and harness materials that are appropriately made to minimize the risk of injury. Here Mauro is showing us and the owner of a donkey how the design and repairs of this saddle lead to injury, especially with the protruding nails that can be seen on the photo on the right.

A good portion of my day here was spent on two more involved cases. Anne and I joined up early in the day when our Mexican partners were busy doing more specific jobs and there were so many horses that still needing to be seen. One of the first horses that we checked in together, was a 3-year-old mare that had over 10 sarcoids, that we could see during our examination. We consulted with Dr. Kinsley to see if she would make a good surgical candidate, and decided that she had at least five that really needed to be removed before they created problems for her ability to eat, or walk. Anne and I were fortunate enough to assist Dr's Kinsley and Casillas as we removed as many of the sarcoids as we could safely do and sutured up the sites. Most of this surgery was completed with head lamps and flashlights lighting up our field of vision. It was a great opportunity for Anne and I to practice our skin suturing skills, and to learn how to be more quick and efficient at our task. However, the group that had to complete a castration after our mare woke up had an even greater challenge. Now that it was completely dark, everyone that could was holding whatever light source they could find and lighting up the surgical field, and as much of the animal as possible for the anesthesia team. This was a very unique experience, and one we worked hard to not repeat on our future work days.

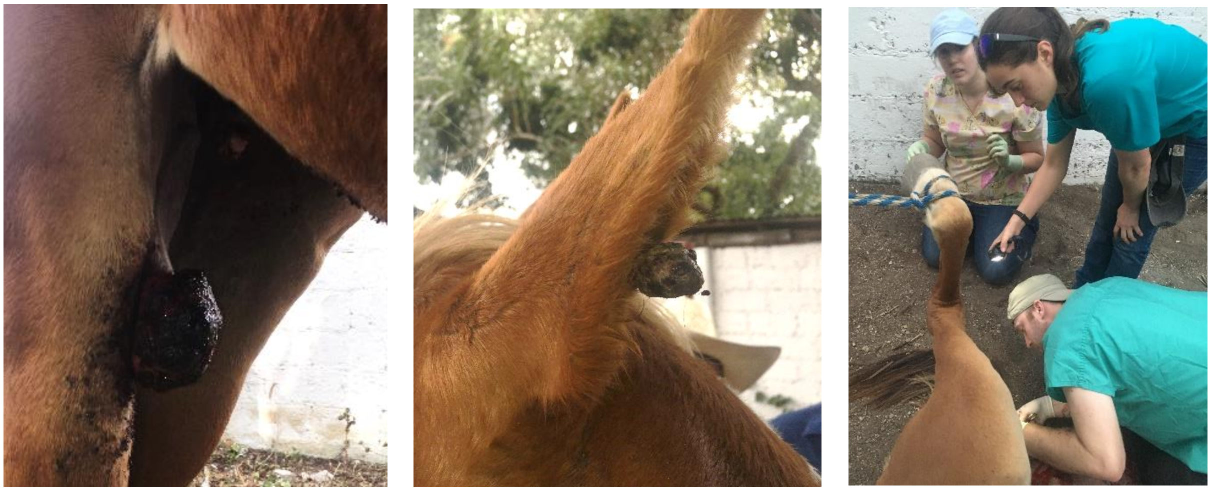

One of the more common skin problems seen in Mexico are sarcoids. This mare had at least 10 sarcoids and we waited until the end of the day to anesthetize the mare for removal of as many of the skin tumors as possible.

Anne and I, also, had the opportunity to work with a fairly critical patient throughout most of the afternoon. We had some challenging discussions with fellow students and our clinicians about what would be our ideal plan of action versus what was feasible in our current situation. We learned some important lessons about the difference between rural field medicine and our typical hospital learning environment. We were able to determine that this mare was severely dehydrated and convince the owner to leave her with us for the rest of the day. We administered four rounds of enteral fluids through a nasogastric tube that we passed every hour. The owner went and bought some pediatric electrolyte supplements from a local human pharmacy that we were able to administer with the fluids. Some students were able to practice passing a urinary catheter, and performing a transrectal palpation. This poor mare let us complete all of these tests without any sedation, which goes to show just how crummy she was feeling.

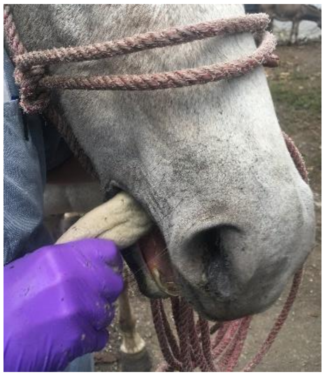 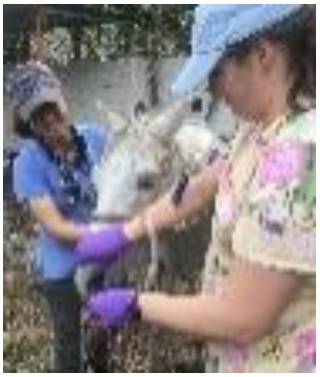 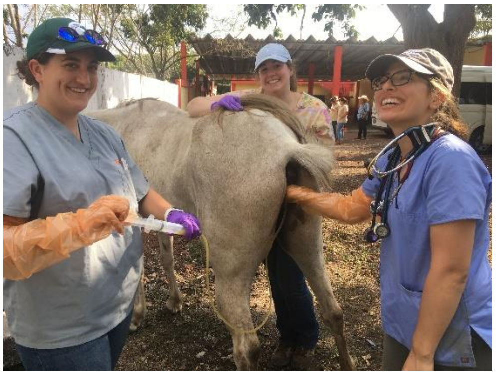

This gray mare arrived at the compound quite dull and had a heart rate of 72/min and absent intestinal sounds. She also passed a small amount of diarrhea. We were careful to remember to put on gloves to examine her as rabies had to be on the list of differential diagnoses. When we examined her mouth we found a foul smelling whitish tongue (left) but no primary problem of the oral cavity was presented. There was a plaque of swelling on the left side of the sternum and a “spider bite” was suspected by the owner. We gave her several doses of enteral fluids over the course of the day and also collected urine and finding concentrated urine told us that the kidneys were still working. By the following day she was reported to have improved and started to eat again – we were suspicious of a colitis but it was challenging to evaluate and treat this case without the luxuries of ultrasound and lab work.

As we went to pass the NG tube for the 3rd round of fluids, we found an irregular heart rate and rhythm, changing from the mid-50s to >120/min, and we suspected that our patient was going in and out of ventricular tachycardia. This led us to a more guarded prognosis and we explained to the owner that we were doing everything we could, but the situation was not looking great and that the mare could suddenly develop a fatal arrhythmia. The owner had to stay for one of his other horses to undergo surgery, so we we’re able to do a final round of fluids and continue to monitor her for a few more hours. By the time she left, she was continuing with multiple arrhythmias. I believe at one point we could hear both a sporadic tachyarrhythmia with concurrent heart block while listening for 1-2 minutes at a time. Miraculously, the mare was still alive and had started eating when we got word from the owner the next day.

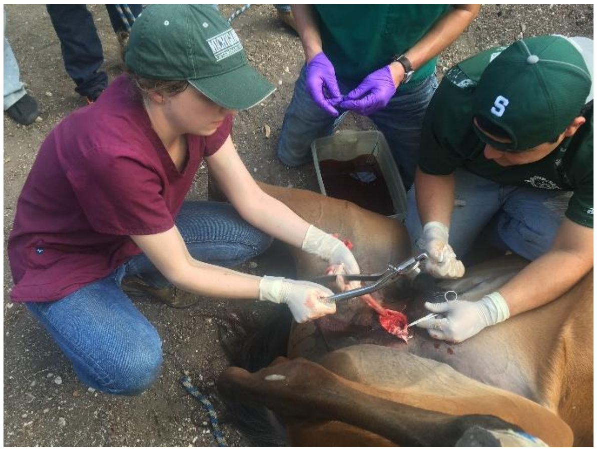 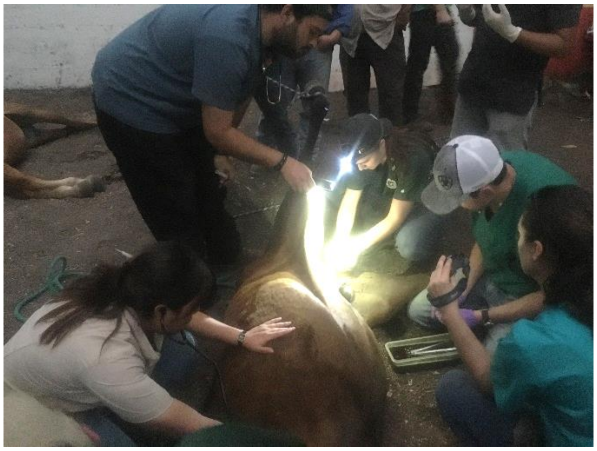

A busy day for castrations: Kathleen preparing to emasculate a testicle (left) and the final castration of the day was performed by Skylar and Dr. Casillas using headlamps and phone flashlights (right).

Another unique experience was that we saw so many animals that we ended up having to complete some of the patients surgeries after dark via flashlight and headlamps. Skylar and Jorge did an amazing job with their after-dark castration. Although, I think Dr. Kinsley, and Dr. Casillas aged a couple of years during the process.

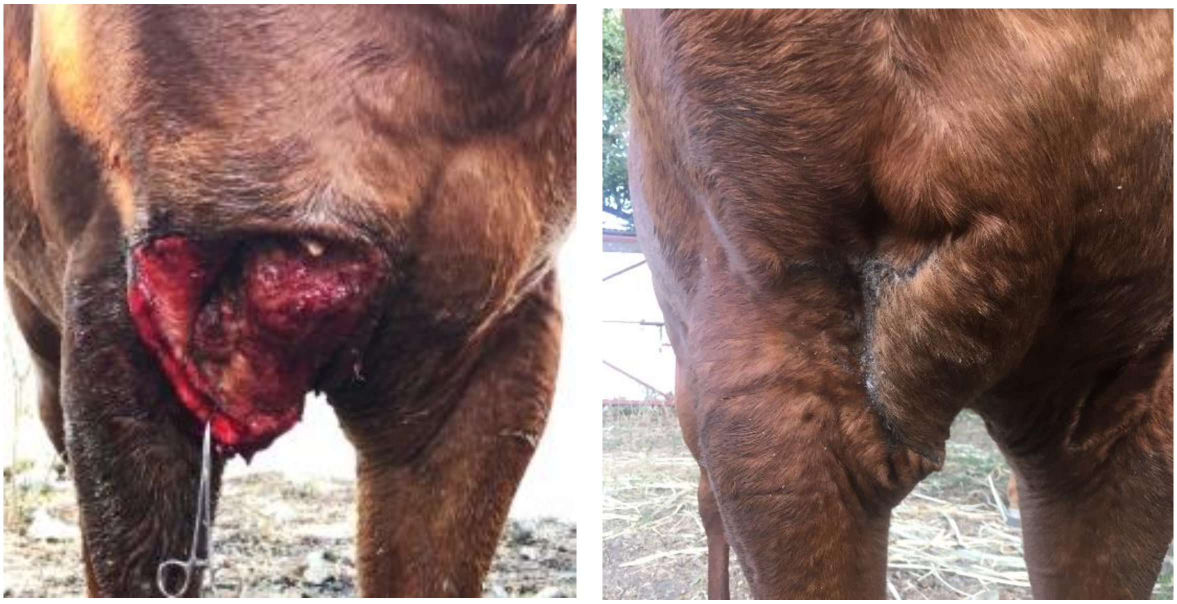

A repeat visitor allowed us to see how a wound that we debrided a year ago (left) had healed after our treatment 1 year later (right).

By the end of the day, the citizens of Camaron de Tejada were very grateful for our visit and brought us a late lunch/early dinner. They were also hopeful that our group may return next year. We even had a chance to see a return visit of a mare that had a large chest wound debrided by our team the previous year. Overall, Day 1 of working out of Boca del Rio, Veracruz was a huge success. Three more days of work to go and we had already learned more than we thought was possible in such short amount of time. I will forever be grateful for the opportunity to join this trip and help these animals.

Day 12 – Wednesday 1/30/19, by Rachel Baumgardner and Héctor Miguel Herrera Lara

“Sorry everyone, we have to leave the hotel by 6:30 tomorrow morning.” Dr. Schott’s kind but stern words were in our heads as we set our alarms for the earliest departure that we would have to venture out for on this rotation. I was not thrilled about the day ahead, for two reasons: 1) My body had JUST gotten used to the sleep schedule for the trip, and 2) The only caffeine guaranteed for us was brown-water “coffee” provided in the hotel rooms. But, I didn’t want to be left behind in Veracruz and miss the infamous burro community of **Santa Maria Tatetla**. So, I woke up on time, drank the brown water, and got into the van.

The 3 plus hour drive was fun, if your definition of fun is cliff side roads. More than once I got a little vertigo looking out the 12-passenger van window into the valley below. Santa Maria Tatetla is one community in the mountains surrounded by many others,

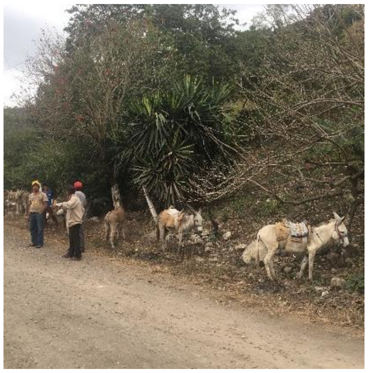 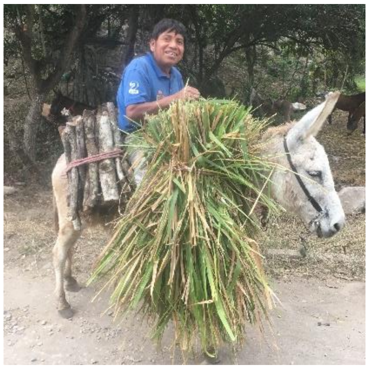 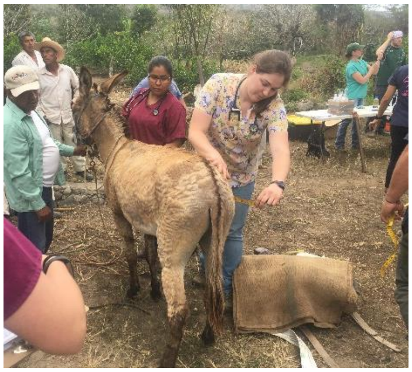

Donkeys were lined up and waiting for us as we arrived at Santa Maria Tatetla (left), although some burros were still working carrying firewood and forage (center). Despite an overwhelming number of equids presented for care, it was essential to continue the process of triage, include measuring abdominal circumference and body length (demonstrated by Anne and Yajaira, right) to accurately estimate body weight.

and was the only stop in the area we were going to make; our trip was going to be servicing a community of approximately 3000 people. Santa Maria Tatetla lived up to its reputation of being the busiest day of the trip. When we arrived, more than 40 donkeys were already waiting for us to vaccinate, deworm, and address other health problems. By the time we set up and started our intake process, the waiting list was almost 2 pages, front and back. Today became known as donkey day, or D-day for short.

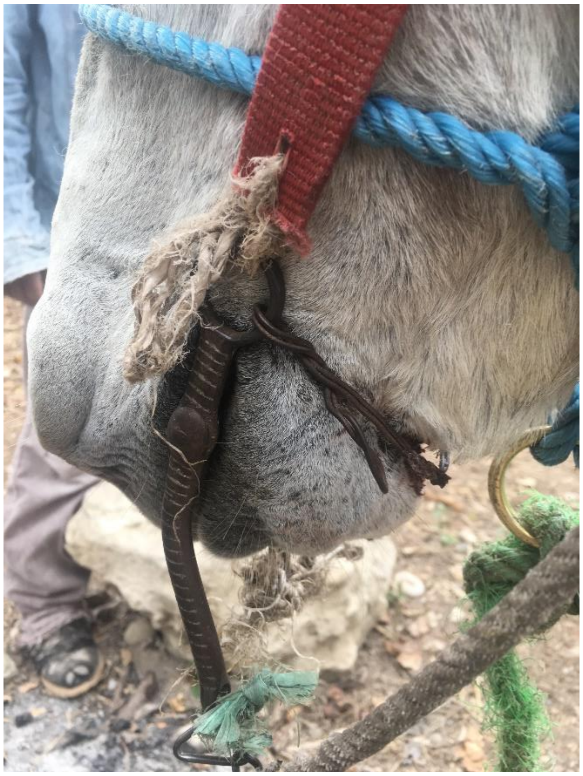 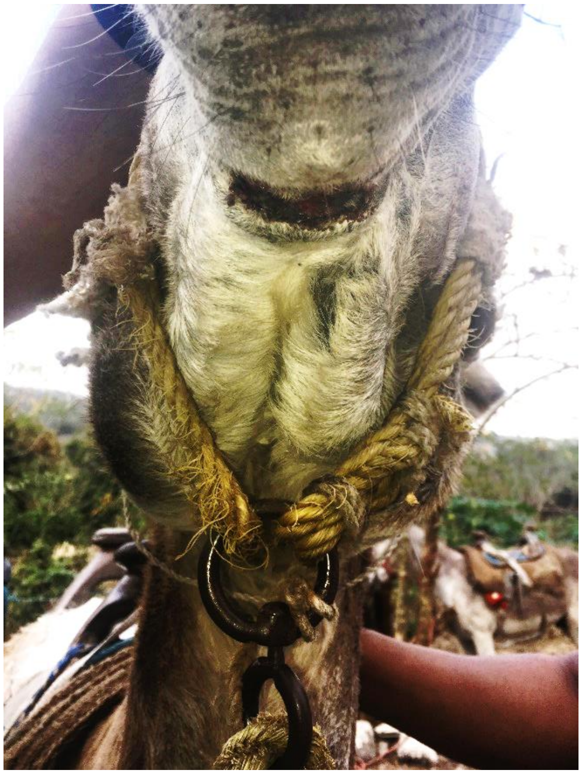

Wounds under the chin from wires used as curbs were seen in several donkeys today.

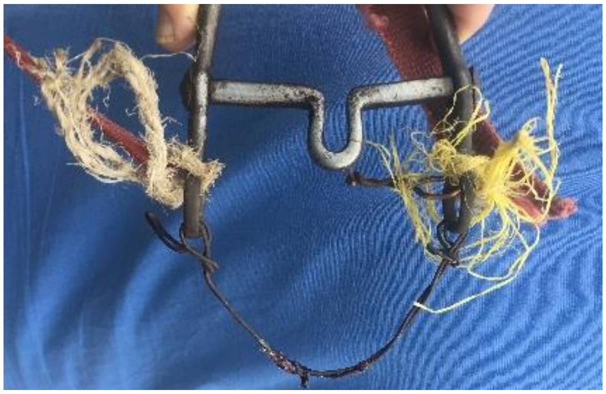 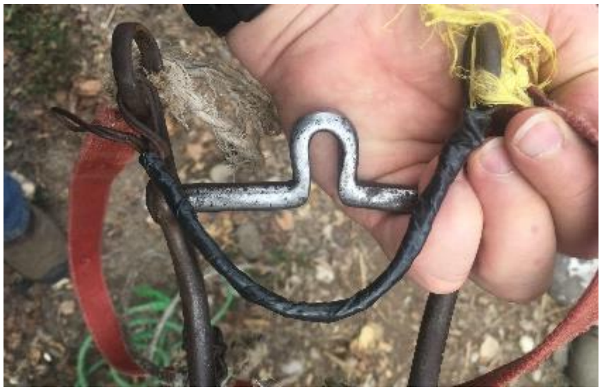

We were able to help this problem a bit by using electrical tape to cover the wire and to educate owners about changing from wire to broader leather or rope straps.

I hadn't worked with many donkeys before but by the end of the day in Santa Maria Tatetla we had provided care to 350 equids, including about 250 donkeys. It was a record-breaking day for the past 3 years of the rotation! The road was awash with donkeys, mules, and horses and the entire day it sounded like a symphony tuning up for a concert that never quite started – brays of varying duration, pitch, and volume filled the day and the quiet at the end of the day was almost palpable.

Abdominal circumferences and body lengths were measured, ivermectin and vitamins were dosed orally, and vaccinations were injected left and right. The wait for farrier work and dentals was at least an hour long. We worked tirelessly all day; we performed many surgeries, including castrations, sarcoid removals, and an abscess drainage. For me, I started to work with the mindset of a car mechanic. The questions in Spanish became automatic. My Spanish partner Hector and I worked like a well-oiled machine to get burros what they needed.

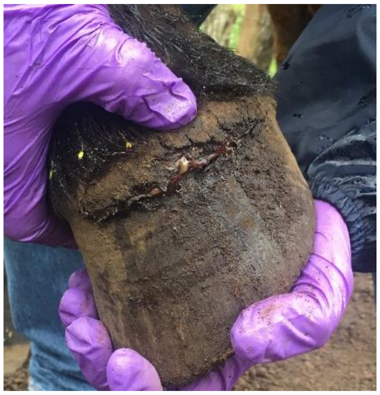 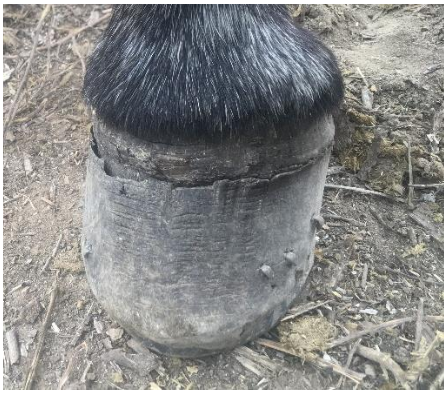 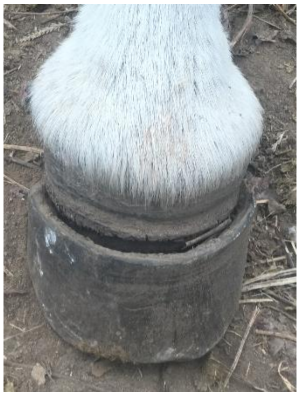

An interesting hoof malady that we saw at several communities was separation of the hoof capsule from the coronary band. This problem usually affected only one hoof and the front feet were more commonly affected than hind feet. Varying degrees of lameness were present but most affected equids could not work for the 9-12 months that was necessary for the hoof to regrow. The cause is unknown although Dr. Alex thought it may be due to Vesicular Stomatitis as there were sometimes oral lesions with acute cases. In the communities, the problem was attributed to spiders, specifically a spider urinating on the coronary band?? Here are pictures of an acutely affected hoof with purulent drainage at the area of separation (left) and two hooves (center and right) where the problem was resolving with no further drainage but lameness was present due to instability of the hoof capsule.

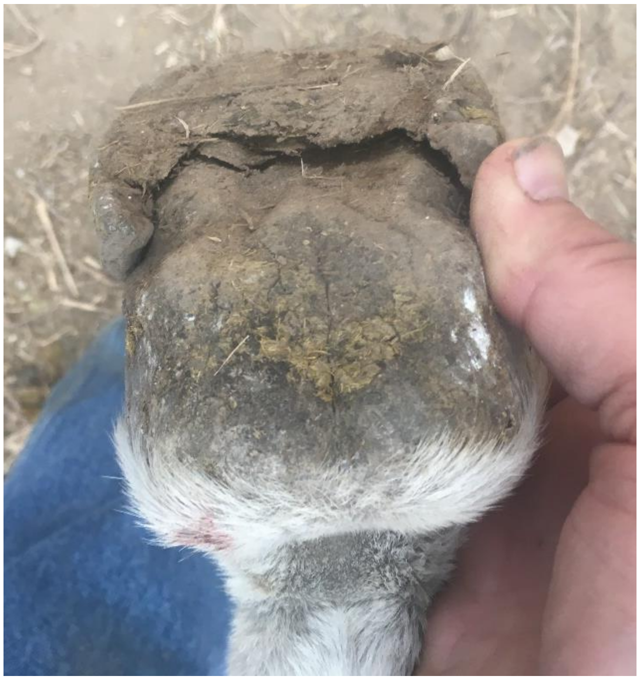 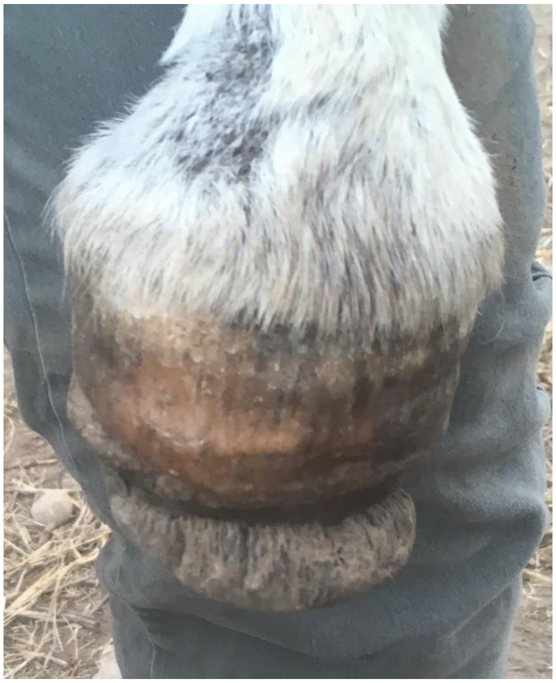

More advanced cases of the interesting hoof malady in which the remaining hoof capsule could nearly be pulled off the underlying tissue (left) and or had fallen off (right). There was little we could do for the chronic condition and we were reluctant to remove any remaining hoof capsule as it was likely the most effective support for the affected hoof. For cases with exposed soft tissue, it had largely cornified and we recommended applying any material available to simply provide a bit of protection to the soft exposed hoof tissue.

After 5 or so hours of working our way through the long list of burros, Hector and I got a chance to talk with some of the community leaders about what these working equids truly provide the community. We learned that the majority were burros, because of their hardiness in the mountainous terrain. These burros typically are used for transport of agricultural products (corn, peanuts, beans, sugar cane, and fruit) and people who work in the fields. These equids work all year long, and typically begin working around 2.5 years of age. We saw several donkeys from 20 to 25 years old during our day of veterinary care – they continue in work as long as they remain able.

Not surprisingly, due to the heavy workload of these equids, we saw a number of wounds from bridles and harnesses. We did our best to clean the lesions and asked Mauro to talk with many equid owners about how to improve their tack to minimize further wounds. We also saw a number of equids with vampire bat bites along the neck and shoulders. Interestingly, vampire bats inject a local anesthetic when they bite that allows them to hang on the neck/shoulder areas during the evenings and acquire their blood meal with little objection from the equids. We also saw a horse with a painful eye that Dr. Fischer suspected had a corneal stromal abscess, likely consequent to a perforation of the cornea. It is always challenging to have a limited ability to treat eye problems in these rural communities and the end result, unfortunately after a fair bit of pain, may be loss or shrinkage of an eye to the end state of a phthisis bulbi.

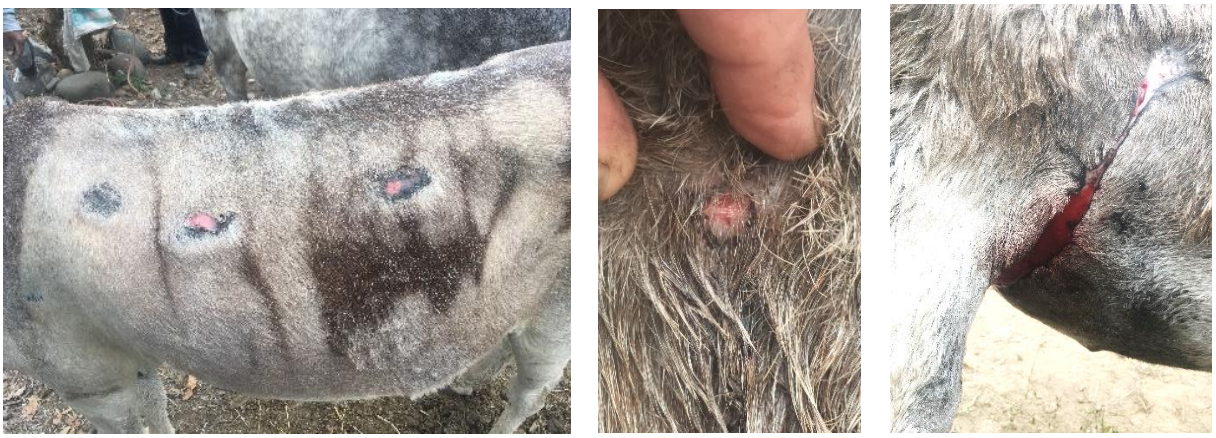

Unfortunately, with hard work comes saddle rubs and wounds (left) as well as challenges of living in a rural environment with vampire bats (center, vampire bat bite wound). The poor donkey on the right had healing wounds in both inguinal regions from getting caught over a wire fence – he apparently also amputated the end of the penis during this traumatic episode as his hindquarters must have been stuck over a wire fence for several hours or longer – it is amazing how the body heals with minimal care.

Although it's not an easy life, the working equids we saw in Santa Maria Tatetla were strong and lively. Veterinary care is not readily available in the remote areas up in the mountains. The people who work in the fields routinely shoe their own equids and try their best to maintain their health. They were all extremely grateful to have us, even if it was just for the day. In the end, one gentleman let us ride his sweet, gentle burro, and the community fed us a dinner in the community center.

Although it was hard work, and I got kicked at more times than I'd like to admit, I had an excellent experience. I met so many hardworking men and their burros, and got to see quite a few interesting medical cases. I was glad for my bed when we returned to the hotel sometime after 10 pm (with the restaurant closed), but I returned feeling fulfilled.

Day 13 – Thursday 1/31/19, by Megan Ahearne and Stephanie Ortiz Guitierez

At our second to last community, Stephanie and I had the pleasure of interviewing the three leaders of **Tenenexpan**, a widespread community outside of the city of Veracruz. Our team was hosted at the stud farm of Senor Victor Hugo Barrera Rios, an accountant in Veracruz. The stud farm was quite nice with a barn for the stallions, including Quarter Horses, Appaloosas, two Miniature Horses, and three donkeys. This setting was surprisingly different than our previous work sites and was surprising to me until I asked Stephanie how these very nice stallions were connected to the working animals that were our mission to serve. Her response was enlightening: Senor Rios allows the local community members to breed their mares to his studs for free, with the goal of increasing genetic diversity and strength of the local equid population. Most of the working horses were in good condition: perhaps a combined result of improved breeding and the pride owners have in their animals?

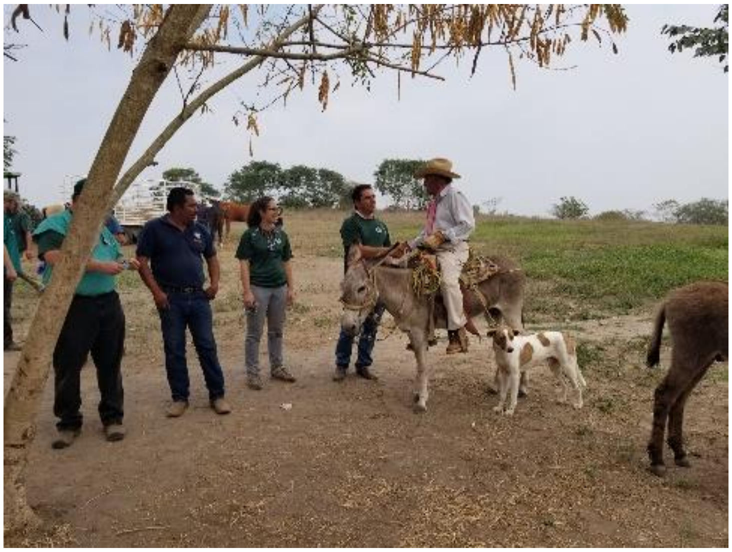
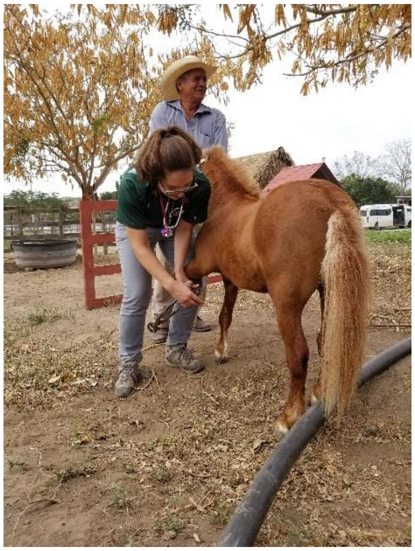

The more arid terrain made it was clear that we were no longer the rainforest, although we were actually pleased with the overcast skies that prevented the day from getting too hot. This community had about half horses and half donkeys and the 90-year-old gentleman that rode in (left) was still using his donkey every day for transportation. Roya (right) enjoyed trying to trim a barely broke Miniature Horse stallion's hooves and learned about the benefits of a good dose or oral detomidine that calmed the rambunctious younger down to become more manageable to work with.

Our day began as usual with unloading the trucks, setting up the tables, and identifying areas for workstations including surgery, farrier work, and dentistry (usually under trees to use a rope to support the heads). By now we could practically do our set up in our sleep! We had a slower start, anything would now be considered slow compared to D day. Despite the sluggish start, we still serviced over 80 equids. We performed two surgeries (a castration and a sarcoid removal), six floats (including a very elderly donkey), and 12 hoof trims (many of which were on semi-feral [i.e., wild] youngsters). It was a good day for learning about proper handling of young horses, as well as a great opportunity to learn about handling stallions (eight were housed at the ranch).

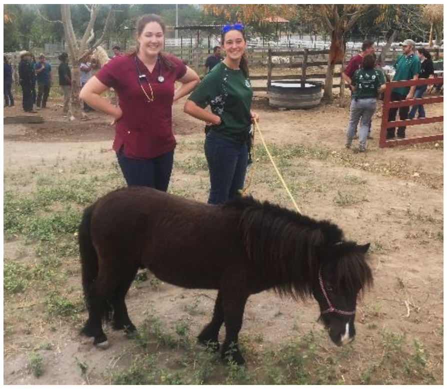
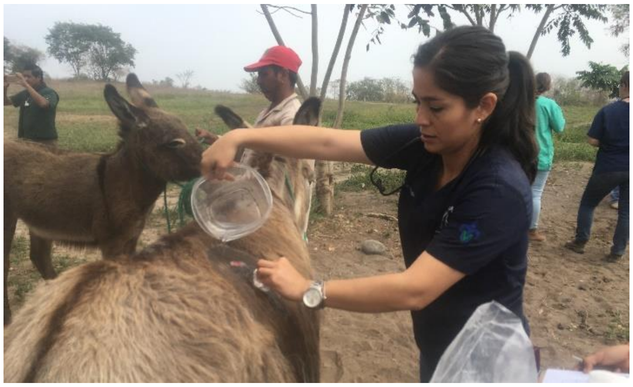

Anne and Megan are admiring one of the ranches Miniature Horse stallions (left) while Dr. Miriam is busy cleaning a donkey's saddle wound (right).

The more spread-out community of Tenenexpan consists of over 4500 individuals and is home to an estimated 500 equids, with an almost even split of horses and donkeys. Much like other communities, the job of transport is fast being taken over by motorized vehicles, especially as the city quickly encroaches on the farm and ranch land previously held by these individuals. Thankfully, the ways of the past are still being upheld by elders in the community, who are working to pass the traditions down to the younger generation, including traditional Spanish horse dancing (demonstrated by Dr. Casillas on one of the Andalusian stallions on the farm).

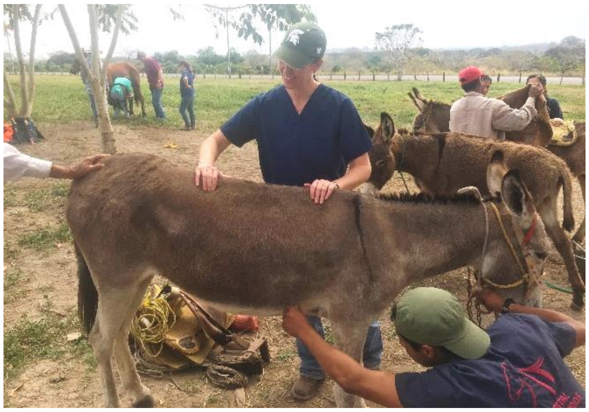

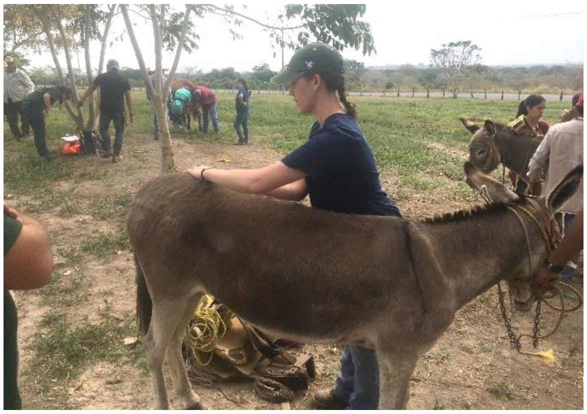

A donkey with a sore back is examined by Dr. Esser

While we provided much needed services to the working people of Tenenexpan, they do have a local veterinarian. At first, we worried about taking away work, but after listening to the stories of the owners we realized that they often don't utilize the veterinarian's services due to expense. They also have a local farrier and almost 100% of working horses are shod, while only about 5% of the burros had shoes. One of the other major features of our day at Tenenexpan was client education. A major concern for both horses and burros was malnutrition. Unlike the communities in the rain forest we serviced in the first week, where there was no shortage of various types of natural forage, pasture was in shorter supply in this more arid climate and the daily cost of feeding horses had risen to more than 100 pesos per day (about $5).

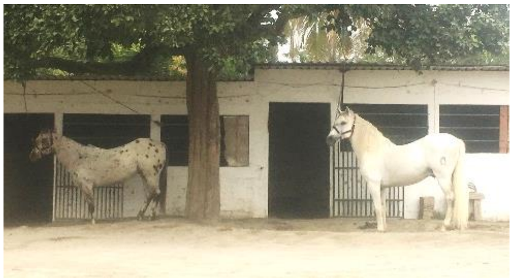

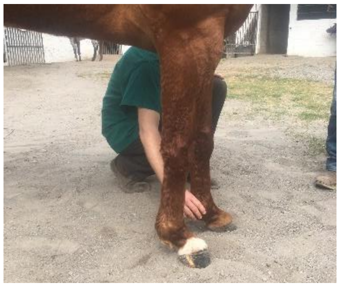

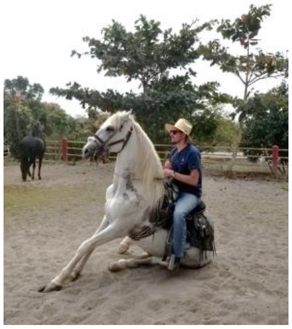

The stud farm of Senor Victor Hugo Barrera Rios was our work site for the day. There was a nice stallion barn (left) that housed both healthy stallions but also a match racehorse that had been retired due to a shoulder injury but now had developed a club foot and a dropped fetlock (center). Some of the horses had been trained for "dancing" and trick riding as demonstrated by Dr. Casillas (right).

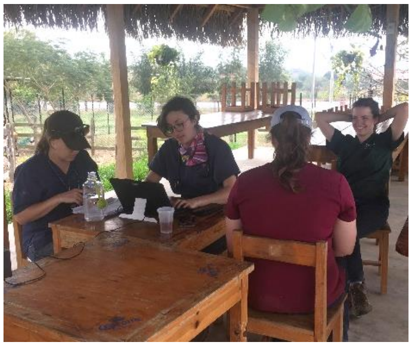
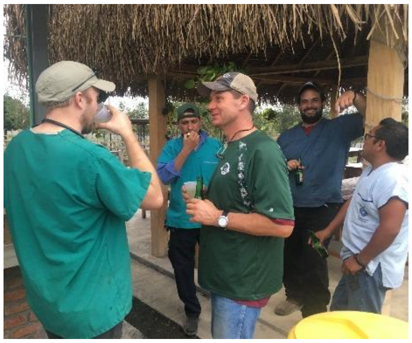
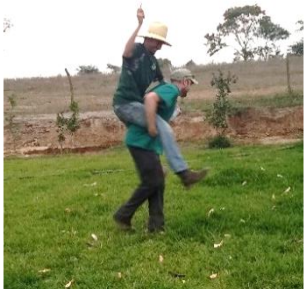

The ranch had a nice covered barbeque area and a palapa for celebrations. The area was quite useful for entering data from medical records into the computer (Katelynn and Rachel, left) during the work day but was even more enjoyable for a warm meal cerveza, and tequila at the end of the day (Drs. Kinsley and Bunn, center). As the tequila did its job, Dr. Kinsley shows that he can work as hard as a burro as he gives Dr. Casillas a"burro race" ride!

At the end of the day we celebrated with a meal of traditionally roasted cow head (tongues and brains included). A new experience for many of us, but an authentic one nonetheless. After a hearty meal some of us participated in "burro races". Overall, it was another successful day making an impact in Mexico. Bittersweet, knowing our trip was coming to an end soon!

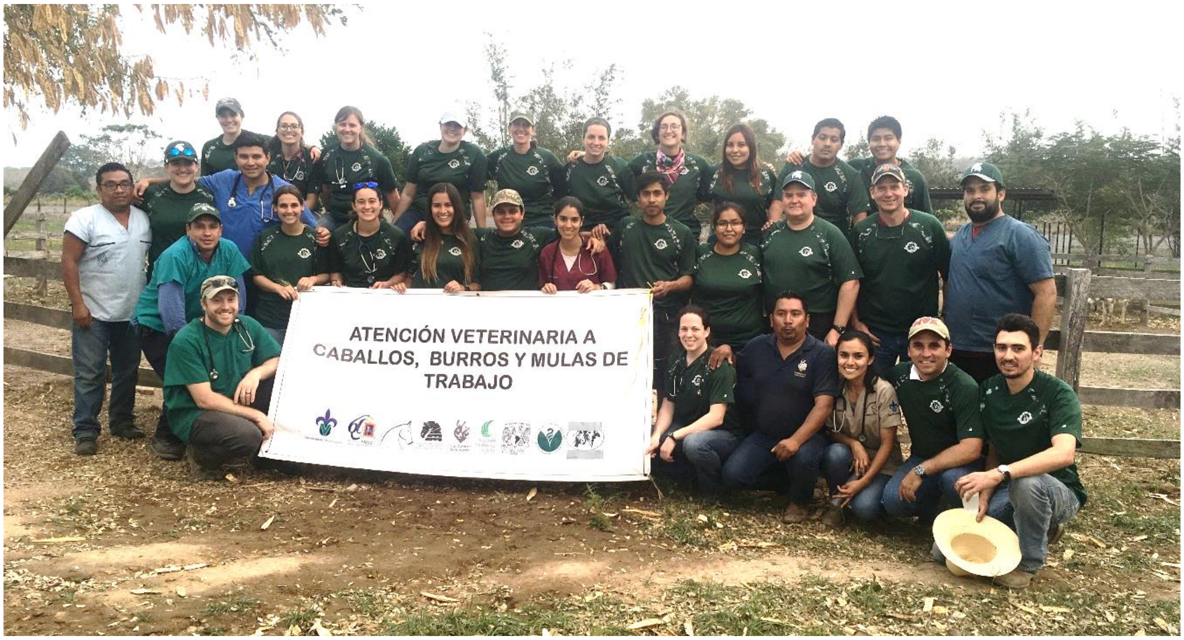

Another successful day for the Equine Welfare in Practice team, now a well-oiled machine!

Day 14 – Friday 2/1/19, by Katelynn Youatt

Following breakfast we piled all of our equipment into the truck and vans one last time and set out for our final day of community work day in **Zempoala**. Unlike many of our other set-ups, Zempoala had a community center that had been designated for us to work at. There was a field in front of a large walled structure that was used for many

community celebrations. Painted at regular intervals along the inside of the structure were black stylistic local animals against the contrast of white painted walls – each animal had the Spanish and Indian names next to it. Almost immediately after our arrival, horses began to flow in by both truck and trailer and by bring ridden.

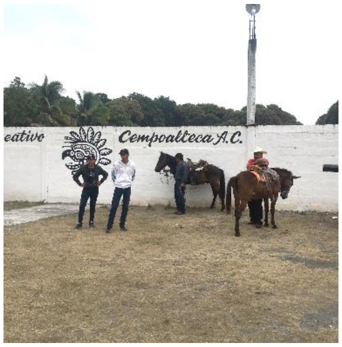

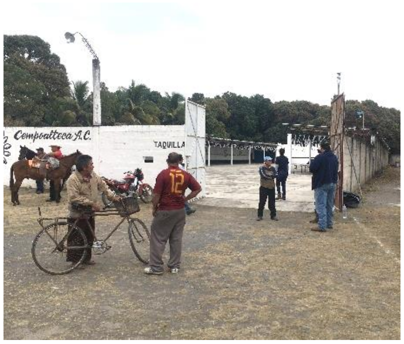

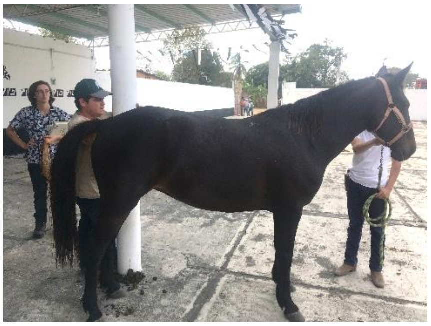

The working site in Zempoala was a large community center with the outside parking area good for triage and the inner are proved useful as a site for mare palpation (Sam and Rachel are examining a mare, right).

Zempoala was the largest (and wealthiest) community we visited with an estimated 10,000 residents. The community visit was actually a last minute add on because the community leader, a friend of Dr. Alex, had heard about what we were doing and asked if we could come for a day. This community had many horses that were utilized as “mascota,” or pets. Horses were used by their owners for transport riding, pleasure, and even in local match horse races. There were also some donkeys in the community used for transporting both milk and fruit products, although numbers had been decreasing dur to availability of trucks and reasonably good roads. Even with equids playing a more similar role as they might in the United States, there was no regular veterinarian living and working in Zempoala. Even the closest tack manufacturer was over 50 miles from the community. This meant that veterinary care was largely provided by the equid owners and that is why they wanted our team to visit the community. Although many equid owners had learned better, we still heard about some interesting treatments such as putting hot ash and used diesel oil on animals to treat ectoparasites.

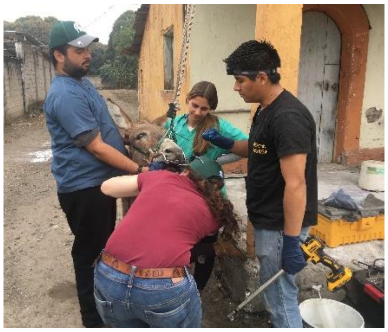
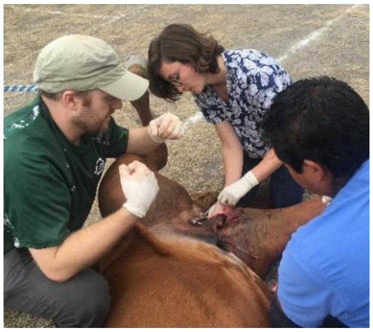
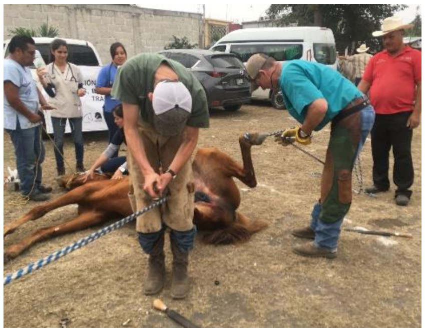

Dentistry (Bernie, Jorge, and Zelia working with Dr. Fischer, left), castration (Rachel and Gerardo working with Dr. Kinsley, center) and farrier work (Drs. Casillas and Bunn took advantage of this rambunctious 2-year-old being under anesthesia to trim its feet, right).

As mentioned, racehorses were more common in this community. One of the first cases we saw was also one of the most memorable. A young, 2-3 year old colt was having gait problems and the owner was concerned that the horse may have had some injury before she bought it as a prospective racehorse. After our physical exam and evaluation, we found the colt to have hind limb ataxia and a mild hypermetria of the front limbs. This spurred a more thorough neurologic examination. Ultimately, our young colt showed signs of compressive myelopathy in the cervical (neck) region. The common cause of this is a condition called Wobblers. It was so amazing to get to work up a whole neurologic case in the field, and satisfying to feel like we had a real answer and some recommendations for this owner moving forward. She was so grateful for the information that she graciously brought the young horse back later in the day for the group to have grand rounds with!

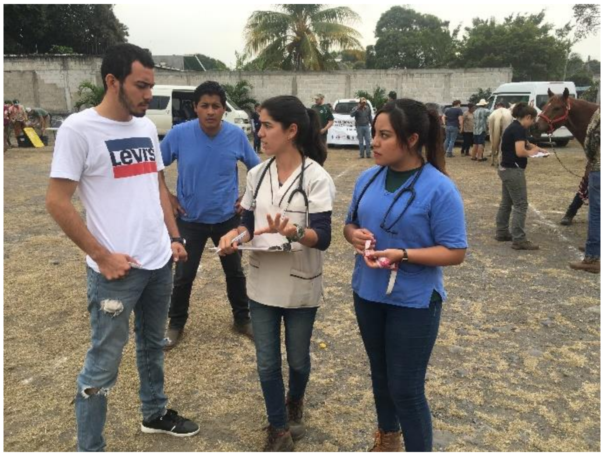

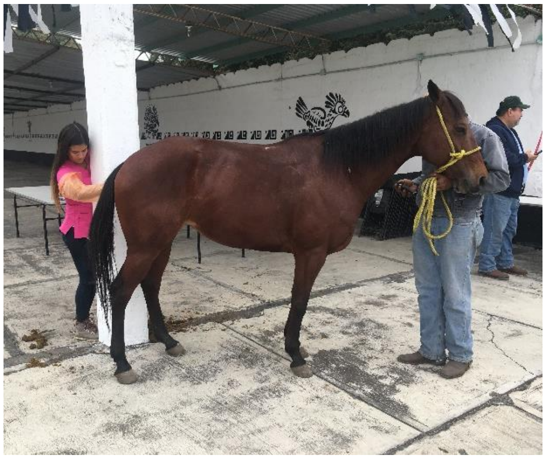

Owner education was an important art of the teams' work as Celina and Karen answer questions from an owner with Hector looking on (left) and Stephanie uses the large pole in the pavilion for protection while palpating a mare (right).

In addition to the unusual cases we saw, there was plenty of farrier and dentistry work to do in Zempoala. By this point in the clerkship, we were able to do assessments on hooves and teeth independently and begin treatment with supervision. It is hard to describe how exciting and fulfilling it was to be in the field like this and experience all of your studying and hard work translating into actual hands-on skills. I know we were all grateful to have such amazing instructors with us who let us grow and develop our knowledge and skills. One of my favorite parts of this last day was getting to do an entire dental float with hand tools (something I certainly would not have been able to do on day one)!

As the day began to wind down, I couldn't help but look around and soak up the last opportunity to work with the amazing colleagues that have been on this trip – both American and Mexican. Pictures and hugs were exchanged, and we all agreed to meet up for dinner in Veracruz to celebrate our many accomplishments – one of the biggest being providing care to over 900 working equids! Reflecting on the entire work from day one, it's hard to be able to say enough about this experience. It is truly something that

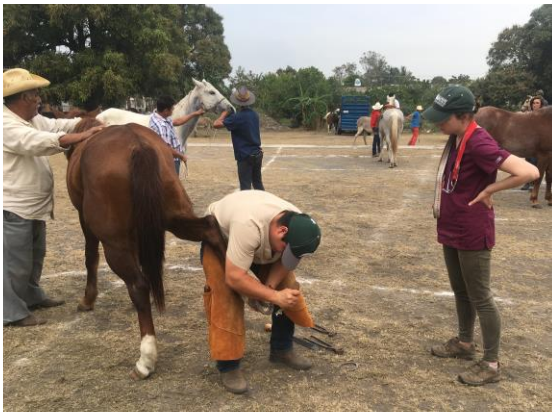

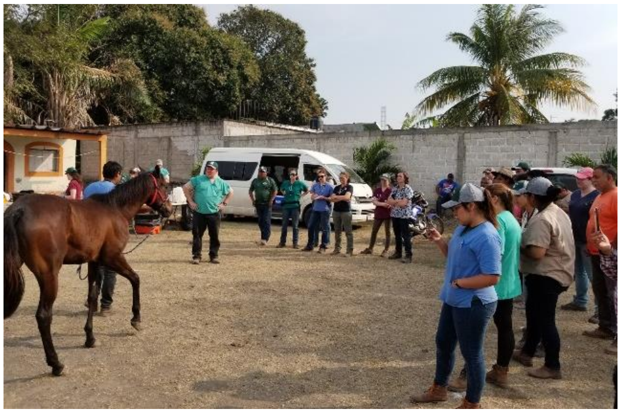

Sam trimming a horse independently with Kathleen looking on (left) and Dr. Schott leading a final grand rounds on neurologic examination with the suspect Wobblers colt at the center of the demonstration.

was life-changing. I know that for all of us, it will stay with us for many years to come as we go out into the world as full-fledged veterinarians.

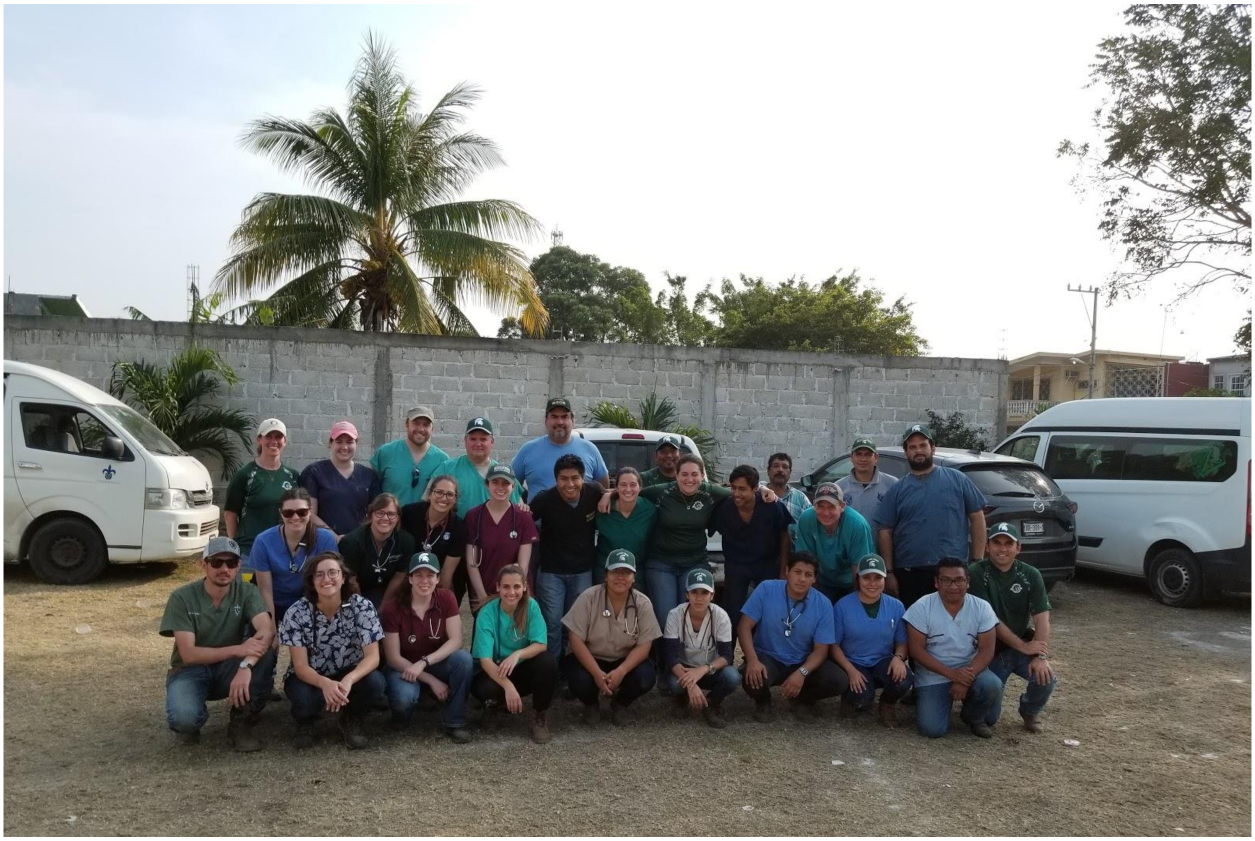

Last working day with an incredible team of teachers, students, and hard working professionals!

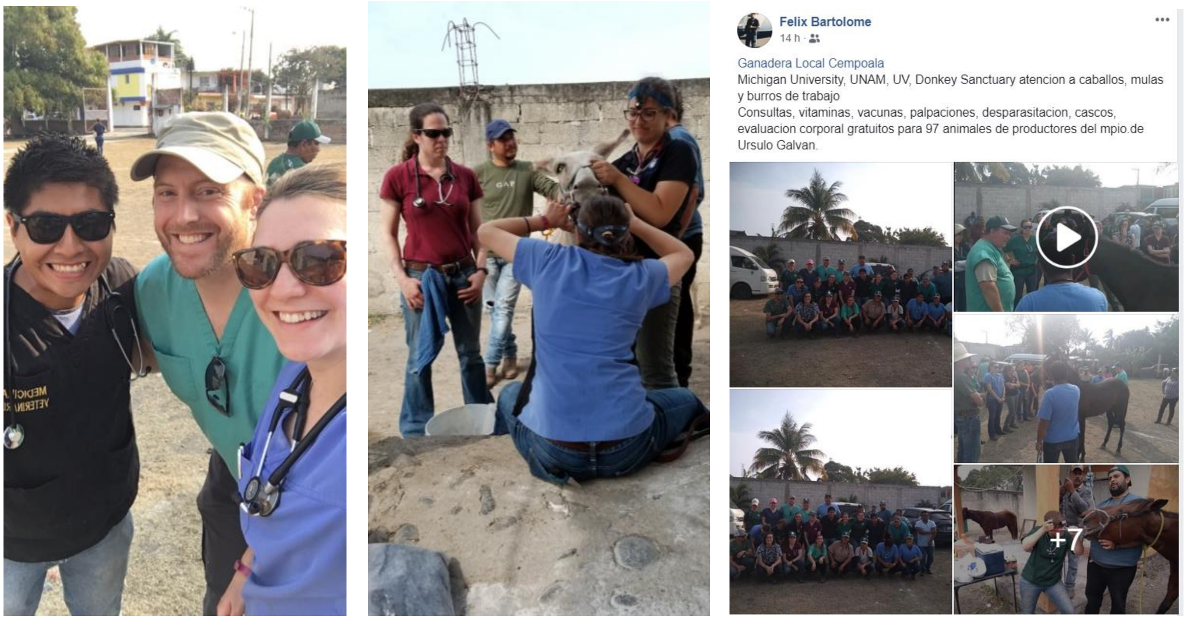

Memories and friendships to last a lifetime: Jorge and Skylar with Dr. Kinsley (left), Roya and Skylar with Dr. Fischer (center), and our second time making the local news (Facebook post from Zempoala, right)!

Days 15, 16 – Mexico City: The Final Days by Dr. Lauren Fischer

After 2 exhausting yet rewarding weeks in the communities surrounding Los Tuxtlas and Veracruz, it was time to begin our journey home. On Saturday morning we loaded into the vehicles for one final road trip – Mexico City bound! For the next 6 hours we watched the landscape change from thick, hilly forests to dry, rocky desert to high, looming mountains. When we arrived in the dense metropolis of the capital city, it was hard to believe that we had climbed over 7000 feet in elevation and that a city this large could be nestled so high in the mountains. The city traffic was crazy, but our drivers delivered us safely to our hotel. We took a couple hours to settle in and grab a bite to eat before Dr. Mariano Hernández-Gil met up with us to give us a tour of Universidad Nacional Autónoma de México (UNAM).

Dr. Mariano led us through a thorough tour of the main campus landmarks as well as the vet school, the only AVMA accredited veterinary school in Mexico. As we walked through the commons, he gave us brief history of the University and the large role that UNAM students have played in national politics. We stopped to admire the main library, its walls covered in an intricate mosaic by Diego Rivera that portrayed Mexico's history, before making a quick stop at the University Olympic Stadium - host of the 1968 Summer Olympics. From there, we took a stroll through the campus grounds to the vet school. Our group admired the open-air hallways and plentiful green space of the vet school, knowing that a polar vortex was engulfing MSU back home. The campus was quiet in the evening light as we passed the buildings for cows, wildlife, pigs, small animals, and birds before we arrived at the equine ward. With its two surgical suites, a

work-up area with stocks, and handful of stalls for in-patients the equine hospital was reminiscent of MSU.

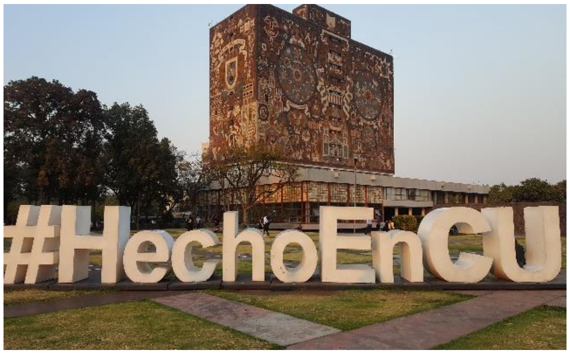

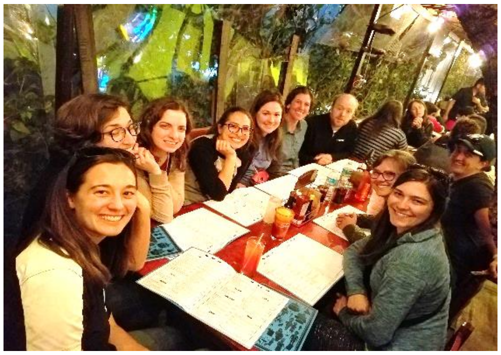

UNAM library with its stone mosaic facade and the common “Made in University City” hash tag (left). Group dinner at Centanaria 107 in Coyoacan (right).

Following the tour, we headed to the Coyoacan neighborhood for dinner at Centanario 107. The restaurant theme and décor made us feel like we were back in the rainforest. Stephanie and Sam, joined us for dinner as a farewell. We reminisced about our experiences and new friendships before calling it a night and heading back to the hotel.

On Sunday morning we headed to el Zócalo, the main square of downtown Mexico City, for some sightseeing. Jorge and Luis joined in to spend our last day with us and one of the UNAM residents, Dr. Lizandra Najera-Casasola, kindly acted as our tour guide for the day. She took us through the Cathedral, a sprawling and ornate church built by the Spanish following the conquest. As we exited, we passed a troupe of dancers in traditional indigenous garb performing blessings to counter bad luck – a stark contrast to the solemn Sunday mass being said just yards away.

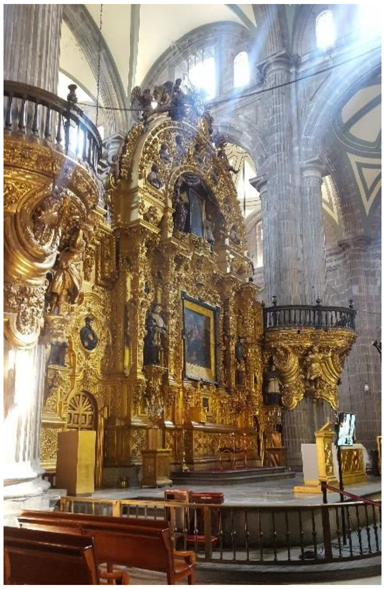
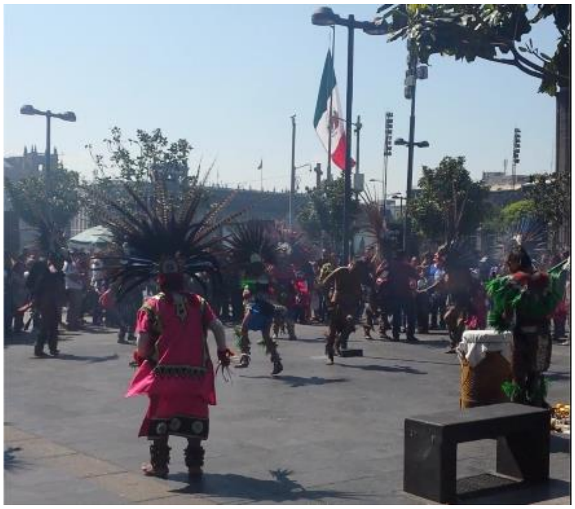

Gilded alter of the Cathedral (left) and traditional indigenous dance performance outside of the cathedral (right)

For a better look at the pre-colonial Aztec culture, we took a tour of the ruins of the Templo Mayor, the main temple of Tenochtitlan, the ancient city on which Mexico City was subsequently built. This ancient dual temple to the god of war and the god of rain was rebuilt six times, the new temple build around the old to make it larger and grander. Upon the Spanish conquest of the city, the great temple was leveled to the ground – it's stones used to build the monuments to Spain, including the Cathedral. The great temple was buried, built over, and forgotten until the 1970's when an electric company discovered the ruins by chance during a construction project. We enjoyed hearing the history of the ruins and examining the religious relics found there.

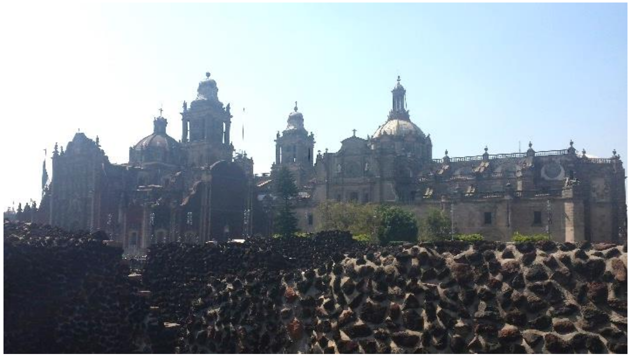

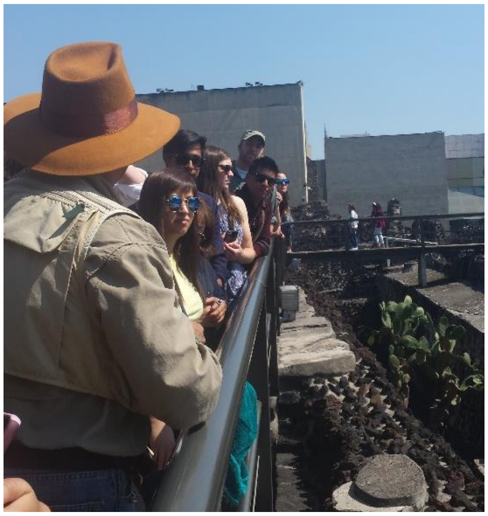

A view of the Cathedral over the wall of the Templo Mayor ruins (above, left).

Our instructional guide explaining the layering of a new temple upon the older temple (right).

For the next stop of the tour, Dr. Lizandra led the group to the open-air Mercado de La Cuidadela for some shopping. The tightly packed, colorful rows of stalls sold handmade art, shirts, and souvenir trinkets. Then we made a quick stop at the Palacio de Bellas Artes (Palace of Fine Arts), which hosts a wide variety of art from music, dance, and opera to painting, sculpture, and photography. We hopped the metro back to the Zócalo – it was quite the feat getting the whole group on and off at the right times. We wandered around the square for a bit before catching the bus back to the hotel. Luckily the hotel was putting on a Super Bowl party, serving a special menu of hotdogs and pizza. At least the company and atmosphere kept us entertained – even if the game didn't. Following the game it was off to bed because in a few short hours, we'd be heading to the airport and to catch our flights home.

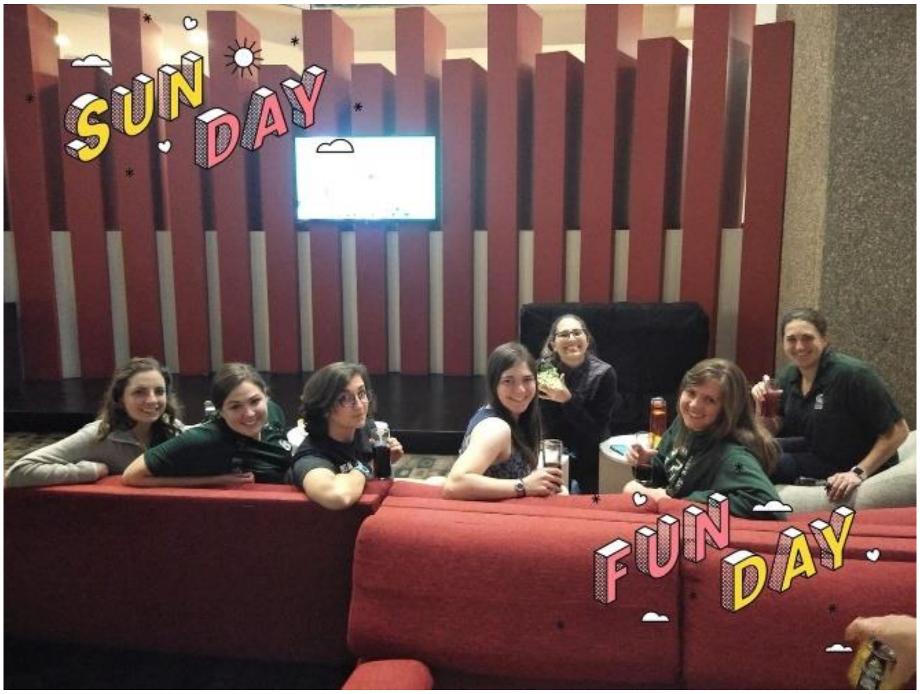

The MSU crew enjoying the Super Bowl

I’d like to close with a few reflections of this whole experience. This clerkship has provided a truly unique experience for all of the students, veterinarians, owners, and animals involved. Some ask, “What can we really accomplish with one visit to these communities?” I would argue quite a bit. The over 900 horses we saw received the first veterinary evaluation of their lives. Those that needed hoof care or dental care received it. The 43 castrations were performed to a high standard of care. Perhaps more importantly, the owners we interacted with were exposed to the benefits of veterinary care. We were able to highlight practical management changes that could improve their animals’ welfare. Mauro, especially, spent a lot of time educating owners on less aggressive handling and training methods. And perhaps most important of all was the training that 20 future veterinarians, from three different countries, received. By the end of this clerkship their clinic skills had improved exponentially in every aspect of routine medicine: handling fractious animals, taking physical exams, giving oral and injectable medications, controlling sedation and anesthesia, performing oral exams and dental floats, trimming hooves, palpating mares, and castrating. As a result of this experience, these students will graduate with a much greater proficiency in general equine medicine. I believe I speak for all of the veterinarians involved – we are very proud of the work they have done on this clerkship.

THE END
